# Synthesis and Structure of Novel Hybrid Compounds Containing Phthalazin-1(2*H*)-imine and 4,5-Dihydro-1*H*-imidazole Cores and Their Sulfonyl Derivatives with Potential Biological Activities

**DOI:** 10.3390/ijms252111495

**Published:** 2024-10-26

**Authors:** Łukasz Balewski, Maria Gdaniec, Anna Hering, Christophe Furman, Alina Ghinet, Jakub Kokoszka, Anna Ordyszewska, Anita Kornicka

**Affiliations:** 1 Department of Chemical Technology of Drugs, Faculty of Pharmacy, Medical University of Gdansk, Gen. J. Hallera 107, 80-416 Gdańsk, Poland; jakub.kokoszka@gumed.edu.pl; 2 Faculty of Chemistry, Adam Mickiewicz University, 61-614 Poznań, Poland; maria.gdaniec@amu.edu.pl; 3 Department of Biology and Pharmaceutical Botany, Faculty of Pharmacy, Medical University of Gdansk, Gen. J. Hallera 107, 80-416 Gdańsk, Poland; anna.hering@gumed.edu.pl; 4 University of Lille, Inserm, CHU Lille, Institut Pasteur de Lille, UMR 1167—RID-AGE—Risk Factors and Molecular Determinants of Aging-Related Diseases, F-59000 Lille, France; christophe.furman@univ-lille2.fr (C.F.); alina.ghinet@junia.com (A.G.); 5 Junia, Health and Environment, Laboratory of Sustainable Chemistry and Health, F-59000 Lille, France; 6 Department of Inorganic Chemistry, Faculty of Chemistry and Advanced Materials Centers, Gdańsk University of Technology, Narutowicza 11/12, 80-233 Gdansk, Poland; anna.ordyszewska@pg.edu.pl

**Keywords:** phthalazin-1(2*H*)-imine, 4,5-dihydro-1*H*-imidazole, imidazoline, hydrazonomethylbenzonitrile derivatives, synthesis, structural studies, cytotoxic evaluation, antioxidant properties, RAGE, hybrid compounds

## Abstract

A novel hybrid compound—2-(4,5-dihydro-1*H*-imidazol-2-yl)phthalazin-1(2*H*)-imine (**5**) was synthesized and converted into di-substituted sulfonamide derivatives **6a**–**o** and phthalazine ring opening products—hydrazonomethylbenzonitriles **7a**–**m**. The newly prepared compounds were characterized using elemental analyses, IR and NMR spectroscopy, as well as mass spectrometry. Single crystal X-ray diffraction data were collected for the representative compounds **5**, **6c**, **6e**, **7g**, and **7k**. The antiproliferative activity of compound **5**, sulfonyl derivatives **6a**–**o** and benzonitriles **7a**–**m** was evaluated on approximately sixty cell lines within nine tumor-type subpanels, including leukemia, lung, colon, CNS, melanoma, ovarian, renal, prostate, and breast. None of the tested compounds showed any activity against the cancer cell lines used. The antioxidant properties of all compounds were assessed using the DPPH, ABTS, and FRAP radical scavenging methods, as well as the β-carotene bleaching test. Antiradical tests revealed that among the investigated compounds, a moderate ABTS antiradical effect was observed for sulfonamide **6j** (IC_50_ = 52.77 µg/mL). Benzonitrile **7i** bearing two chlorine atoms on a phenyl ring system showed activity in a β-carotene bleaching test (IC_50_ = 86.21 µg/mL). Finally, the interaction AGE/RAGE in the presence of the selected phthalazinimines **6a**, **6b**, **6g**, **6m**, and hydrazonomethylbenzonitriles **7a**, **7c**–**g**, and **7i**–**k** was determined by ELISA assay. A moderate inhibitory potency toward RAGE was found for hydrazonomethylbenzonitriles—**7d** with an electron-donating methoxy group (R = 3-CH_3_O-C_6_H_4_) and **7f**, **7k** with an electron-withdrawing substituent (**7f**, R = 2-Cl-C_6_H_4_; **7k**, R = 4-NO_2_-C_6_H_4_).

## 1. Introduction

Privileged structures are molecular scaffolds widely used as templates in medicinal chemistry for the design and synthesis of compounds for a range of different biological targets. Phthalazine (2,3-benzodiazine or benzo[*d*]pyridazine) is a common simple core found in many compounds possessing a rich diversity of biological properties [1,2,3].

**Figure 1:**
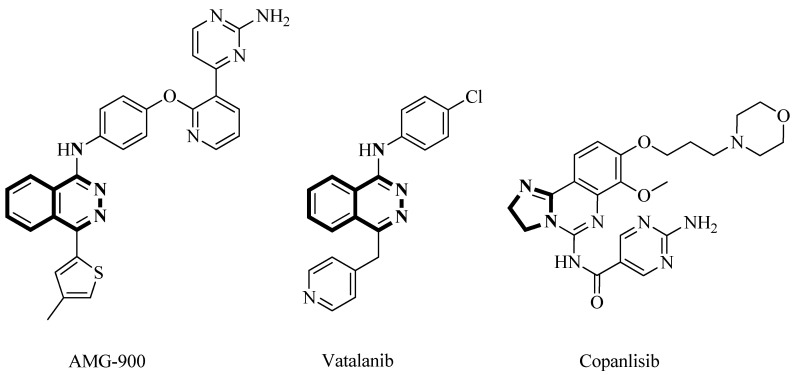
Structures of clinically approved phthalazines (AMG-900, Vatalanib) and imidazoline-fused derivative (Copanlisib) with anti-cancer activities.

In recent years, there has been a great interest in phthalazines due to their anticancer potential and activity against biological targets involved in the progression of cancer. There is substantial evidence that anti-cancer therapy can be based on the serine/threonine protein kinase (Aurora kinase) family, which plays an important role in the progression of mitosis. It was found that phthalazine derivatives may target Aurora kinase as potent and selective inhibitors [4]. An example of a potent and highly selective pan-Aurora kinase inhibitor is the clinically approved phthalazine derivative—AMG 900 (Figure 1), which inhibits the enzyme activity of all three Aurora kinase family members [5]. In addition, phthalazine derivatives have shown a strong ability to inhibit the growth of several cancer cell lines by affecting vascular endothelial growth factor receptors-2 (VEGFR-2) [6,7,8]. For example, Vatalanib—*N*-(4-chlorophenyl)-4-[(pyridin-4-yl)methyl]phthalazin-1-amine—is a novel phthalazine-based oral antiangiogenic agent that inhibits all vascular endothelial growth factor receptors. Currently, Vatalanib is being studied as a treatment for solid tumors (Figure 1) [9]. Furthermore, it has been noted that phthalazine derivatives have anticancer activity as a result of their ability to affect epidermal growth factor receptors (EGFR) [10] or the Hedgehog pathway [11]. Additionally, it has been shown that phthalazine derivatives with cytotoxic properties towards cancer cells also exhibit antioxidant activity [12].

The phthalazine ring has also proven to be an excellent scaffold for the design of readily available melatonin MT_1_ and MT_2_ ligands as bioisosteric analogs of agomelatine [13]. They have been reported to display anticonvulsant properties as non-competitive antagonists of AMPA receptors [14]. Finally, it has been suggested that phthalazines may act as aldose reductase (AR) inhibitors—an enzyme that is involved in the pathogenesis of several chronic complications associated with diabetes mellitus, such as nephropathy, neuropathy, retinopathy, and cataracts [15].

There is growing evidence that compounds based on the 4,5-dihydro-1*H*-imidazole (imidazoline) scaffold are present in a variety of synthetic agents exhibiting a wide range of biological activities [16,17,18,19]. Special attention has been paid to imidazolines with antiproliferative properties, which may serve as potent anticancer agents [20]. For example, intensive research led to the discovery of novel imidazoline-based carbonic anhydrase inhibitors containing a sulfonamide moiety with pronounced antitumor activity [21,22]. Moreover, the imidazoline skeleton constitutes a structural part of the selective pan-Class I lipid phosphoinositide 3-kinase (PI3K) inhibitor—Copanlisib (Figure 1) that activates downstream signaling pathways involved in cell survival and growth. In September 2017, the FDA granted accelerated approval to Copanlisib for the treatment of follicular lymphoma [23].

Additionally, 4,5-dihydro-1*H*-imidazole derivatives have been investigated for their favorable effects on cardiovascular and metabolic disorders [24] as well as neuroprotective [25,26] anti-inflammatory [27] and analgesic properties [28]. Compounds with an imidazoline core were claimed to display antiparasitic activity [29,30]. Furthermore, it was observed that imidazoline derivatives acting on the central nervous system may be useful in treating depression [31].

In rational drug design, molecular hybridization refers to the process of combining two or more pharmacophoric units into a single molecule. This approach has been successfully used, resulting in the development of novel hybrid entities. It has shown a lot of promise as it relates to a variety of multifactorial disorders, such as cancer, neurodegenerative disorders, hypertension, and inflammation [32,33,34].

**Figure 2:**
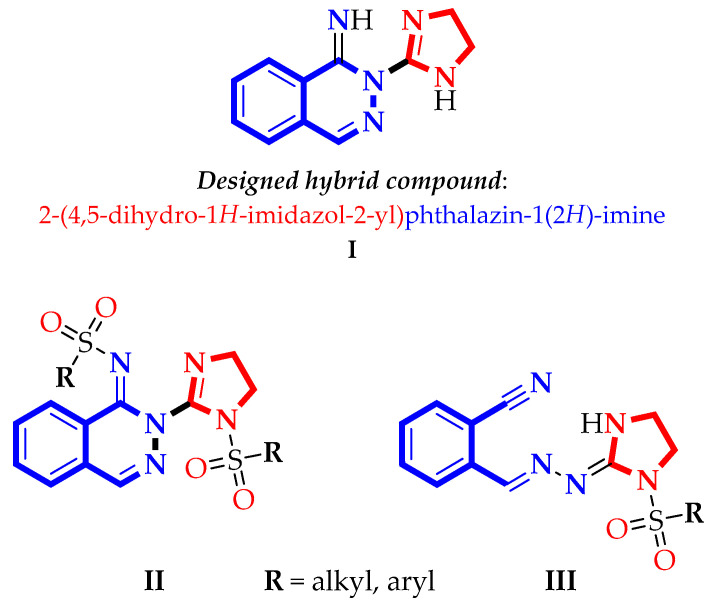
General structure of designed hybrid compound: 2-(4,5-dihydro-1*H*-imidazol-2-yl)phthalazin-1(2*H*)-imine (**I**) and its derivatives: *N*-(2-(1-(aryl(alkyl)sulfonyl)-4,5-dihydro-1*H*-imidazol-2-yl)phthalazin-1(2*H*)-ylidene)aryl(alkyl)sulfonamides (**II**) and 2-(((1-(aryl(alkyl)sulfonyl)imidazolidin-2-ylidene)hydrazono)methyl)benzonitriles (**III**).

As a continuation of our research on hybrid compounds [35,36,37,38,39] with anticancer properties in this work, we designed and synthesized a novel hybrid compound: **2-(4,5-dihydro-1*H*-imidazol-2-yl)phthalazin-1(2*H*)-imine** (**I**) composed of two privileged pharmacophore groups—**phthalazine** and **4,5-dihydro-1*H*-imidazole** and its di-substituted derivatives—*N*-(2-(1-(aryl(alkyl)sulfonyl)-4,5-dihydro-1*H*-imidazol-2-yl)phthalazin-1(2*H*)-ylidene)aryl(alkyl)sulfonamides (**II**). In turn, sulfonamides **II** were transformed spontaneously into phthalazine ring opening products—hydrazonomethylbenzonitriles **III** (Figure 2).

Given the broad spectrum of biological activities exhibited by phthalazine and imidazoline derivatives, we conducted a preliminary evaluation of the antiproliferative and antioxidant potential of newly synthesized hybrid molecules **II** and **III** (Figure 2) to identify novel lead compounds with such a biological profile. Moreover, we decided to expand our research by exploring new compounds as potential ligands for RAGE. Receptor for advanced glycation end-products (RAGE) is an immunoglobulin-like, multiligand cell-surface receptor involved in various pathological processes, including inflammation, neurodegenerative diseases, and cancer, making it a highly attractive drug target [40].

## 2. Results and Discussion

### 2.1. Chemistry

#### 2.1.1. Synthesis of 2-(4,5-dihydro-1*H*-imidazol-2-yl)phthalazin-1(2*H*)-imine (**5**)

The starting phthalazine (**1**) in the reaction with 2-chloro-4,5-dihydro-1*H*-imidazole hydrogen sulfate (**2**) gave rise to the formation of pseudobase: 2-(4,5-dihydro-1*H*-imidazol-2-yl)-1,2-dihydrophthalazin-1-ol (**3**) [41].

**Scheme 1:**
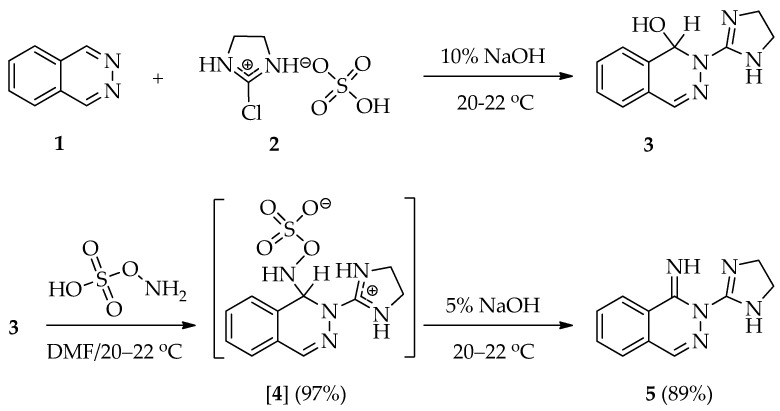
Synthesis of 2-(4,5-dihydro-1*H*-imidazol-2-yl)phthalazin-1(2*H*)-imine (**5**) (isolated yields in paretheses).

Then, compound **3** in reaction with a 3-fold excess of (aminooxy)sulfonic acid (HOSA) in anhydrous dimethylformamide yielded 2-(4,5-dihydro-1*H*-imidazol-3-ium-2-yl)-1,2-dihydrophthalazin-1-ylamino sulfate (betaine) (**4**). The obtained intermediate **4** was used for the next step without further purification. Upon treatment of compound **4** with a 5% solution of sodium hydroxide at room temperature, the desired 2-(4,5-dihydro-1*H*-imidazol-2-yl)phthalazin-1(2*H*)-imine (**5**) was obtained (Scheme 1).

The structure of 2-(4,5-dihydro-1*H*-imidazol-2-yl)phthalazin-1(2*H*)-imine (**5**) was confirmed by IR, ^1^H and ^13^C, 2D-NMR spectroscopy, mass spectrometry, elemental analysis and X-ray crystallographic studies (see Section 2.2 and Supplementary Materials, Figures S1–S7 and S20).

In the IR spectrum of 2-(4,5-dihydro-1*H*-imidazol-2-yl)phthalazin-1(2*H*)-imine (**5**), N-H stretching vibrations of the imine group (C=N-H) phthalazin-1(2*H*)-imine ring and the secondary amine group (N-H) of the imidazoline scaffold are observed in a range of 3310 to 3157 cm^−1^ (see Supplementary Materials, Figure S1).

In turn, the ^1^H-NMR spectrum revealed a characteristic singlet attributable to the proton C4-H of the phthalazin-1(2*H*)-imine at 8.11 ppm and the presence of two distinct multiplets at *δ* 3.33 ppm and *δ* 3.89 ppm integrating four protons attributable to the CH_2_-CH_2_ bridge of imidazoline ring. Two protons of C=N-H and N-H groups appear in the spectrum as broad singlets at 6.94 ppm and 10.87 ppm, respectively (see Supplementary Materials, Figure S2).

The ^13^C-NMR spectrum of compound 5 recorded in DMSO-*d*_6_ solution at 20–22 °C revealed two carbon signals at 42.9 ppm and 54.2 ppm attributable to the C4′ and C5′ of imidazoline ring. Eight carbon atoms of phthalazin-1(2*H*)-imine ring were observed at C8a—125.2 ppm (quaternary), C8—125.8 ppm, C5—127.3 ppm, C4a—129.9 ppm (quaternary), C6 and C7—132.5 ppm and 132.7 ppm, C4—138.3 ppm, and C1 carbon atom of C=NH moiety, 147.9 ppm. Quaternary carbon C2′ of 4,5-dihydro-1*H*-imidazole moiety appeared at 161.7 ppm (see Supplementary Materials, Figure S3). 

For structural analysis of 2-(4,5-dihydro-1*H*-imidazol-2-yl)phthalazin-1(2*H*)-imine (**5**), chemical shift values of hydrogen atoms and their associated carbon atoms were determined based on heteronuclear single quantum coherence (HSQC, see Supplementary Materials) and heteronuclear multiple bond correlation (HMBC) spectra. Analysis of the range *δ*C 115 ppm—165 ppm HSQC spectrum revealed four correlations of carbon atoms: C4, C5, C6/7, and C8. Signals of quaternary carbon atoms: C1 of C=NH group of phthalazin-1(2*H*)-imine ring and C2′ of 4,5-dihydro-1*H*-imidazole did not correlate to any proton resonances (see Supplementary Materials, Figure S4).

**Figure 3:**
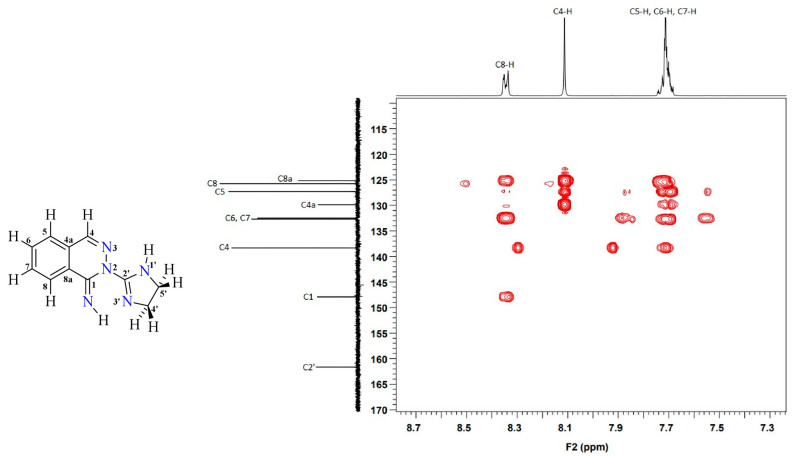
^1^H-^13^C HMBC spectrum of compound **5**.

In the HMBC spectrum of compound 5, long-range correlations have been observed between the following protons and carbon atoms: H4 [δ 8.11 (s)] and C4a [δ 129.9 ppm], C5 [δ 127.3 ppm], C8a [δ 125.2 ppm]; H5 [7.69–7.73 (m)] and C4 [δ 138.3 ppm], C4a [δ 129.9 ppm], C6 [δ 132.5 ppm], C8a [δ 125.2 ppm]; H6 [δ 7.69–7.73 (m)] and C4 [δ 138.3 ppm], C4a [δ 129.9 ppm], C5 [δ 127.3 ppm], C7 [δ 132.7 ppm], C8a [δ 125.2 ppm]; H7 [7.69–7.73 (m)] and C5 [δ 127.3 ppm], C6 [δ 132.5 ppm], C8a [δ 125.2 ppm]; H8 [δ 8.34–8.36 (m)] and C1 [δ 147.9 ppm], C6 [δ 132.5 ppm], C7 [δ 132.7 ppm], and C8a [δ 125.2 ppm] (Figure 3, see Supplementary Materials, Figure S5).

**Figure 4:**
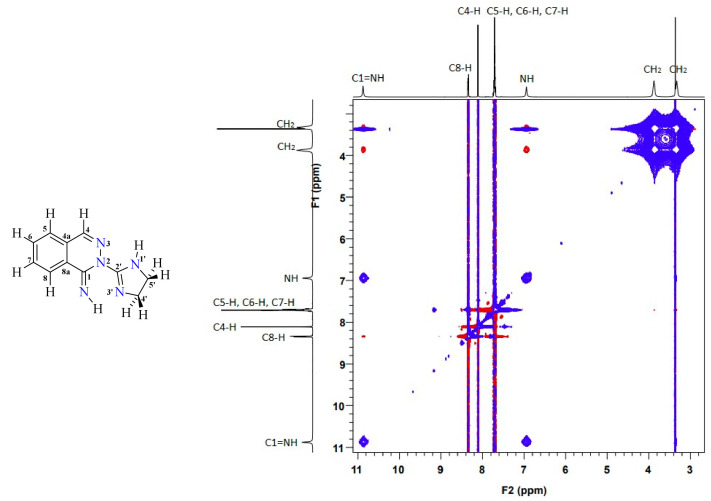
^1^H-^1^H ROESY spectrum of compound **5**.

In order to give better insight into the structural determination of compound **5**, the Rotating-Frame Overhauser Enhancement Spectroscopy (ROESY) experiment was performed. As shown in Figure 4, the ROESY spectrum revealed a strong NOE effect between two N-H groups of phthalazin-1(2*H*)-imine and imidazoline rings. Moreover, intense NOE cross-peaks were observed for N-H and CH_2_-CH_2_ groupings of imidazoline (see Supplementary Materials, Figure S6).

#### 2.1.2. Synthesis of *N*-(2-(1-(aryl(alkyl)sulfonyl)-4,5-dihydro-1*H*-imidazol-2-yl)phthalazin-1(2*H*)-ylidene)aryl(alkyl)sulfonamides **6a**–**o** and 2-(((1-(aryl(alkyl)sulfonyl)imidazolidin-2-ylidene)hydrazono)methyl)benzonitriles **7a**–**m**

Our synthetic interest has been focused on the transformations of 2-(4,5-dihydro-1*H*-imidazol-2-yl)phthalazin-1(2*H*)-imine (**5**). It was found that both the imine group (C=N-H) and secondary amine group (N-H) were sufficiently nucleophilic to displace halogen atoms from acyl or sulfonyl chlorides forming corresponding *N*-(2-(1-(aryl(alkyl)sulfonyl)-4,5-dihydro-1*H*-imidazol-2-yl)phthalazin-1(2*H*)-ylidene)aryl(alkyl)sulfonamides **6a**–**o**. The reactions were carried out in anhydrous aprotic solvent (dichloroethane, DCE) at 90 °C. The progress of reactions was controlled by thin-layer chromatography (see Supplementary Materials, spectra of representative compound **6a**: Figures S8–S11).

Attempts to obtain mono-substituted sulfonyl products failed. We observed only the formation of di-substituted derivatives when the stoichiometric ratio of compound **5** and sulfonyl chloride was 1:1, even at low or ambient temperatures. During the preparation and purification by preparative thin-layer chromatography (chromatotron^TM^) of the crude sulfonamide derivatives **6a**–**o**, we unexpectedly observed the formation of benzonitrile derivatives **7a**–**m** in poor yields (3–22%) or satisfactory yields (34–44%), as side-products of phthalazin-1(2*H*)-imine ring opening (see Supplementary Materials, spectra of representative compound **7a**: Figures S12–S15). 

This was confirmed by the spectroscopic characterization of compounds **7a**–**m**, in which we noted the presence of a nitrile group in the range from 2219 cm^−1^ and 2233 cm^−1^ and bands in the ranges from 3448 cm^−1^ to 3351 cm^−1^ attributed to N-H stretching frequencies (see Supplementary Materials, spectra of compounds **7a** and **7k**: Figures S12 and S16). 

**Scheme 2:**
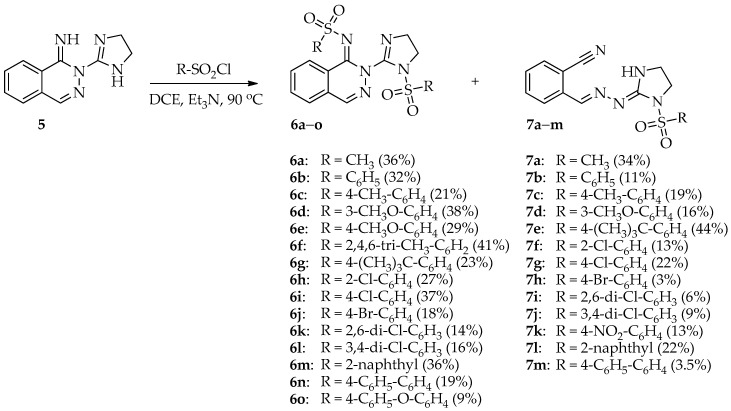
Synthesis of *N*-(2-(1-(aryl(alkyl)sulfonyl)-4,5-dihydro-1*H*-imidazol-2-yl)phthalazin-1(2*H*)-ylidene)aryl(alkyl)sulfonamides **6a**–**o** and 2-(((1-(aryl(alkyl)sulfonyl)imidazolidin-2-ylidene)hydrazono)methyl)benzonitriles **7a**–**m** (isolated yields in parentheses).

Moreover, two characteristic singlet signals, 7.58–7.75 ppm and 8.01–8.43 ppm from N-H and CH=N groups, respectively, were observed in the ^1^H-NMR spectra. In the case of 4-nitrobenzene-1-sulfonyl chloride with a strong electron-withdrawing group (R = -C_6_H_4_-NO_2_-*p*), it was found that the only product formed in high yield was 2-(((1-((4-nitrophenyl)sulfonyl)imidazolidin-2-ylidene)hydrazono)methyl)benzonitrile **7k** (Scheme 2, see Supplementary Materials, Figures S16–S19).

In contrast, the reaction with 4-methoxybenzene-1-sulfonyl chloride containing electron-donating group promotes the formation 4-methoxy-*N*-(2-(1-((4-methoxyphenyl)sulfonyl)-4,5-dihydro-1*H*-imidazol-2-yl)phthalazin-1(2*H*)-ylidene)benzenesulfonamide (**6e**) as the main product with a satisfactory yield (ca. 29%). 

Compounds **6** and **7** absorb strongly in the range, which may be attributed to the secondary sulfonamide group (-SO_2_-NR). The asymmetric and symmetric S=O stretching frequency ranges for disubstituted sulfonamides **6a**–**o** are as follows: 1392–1333 cm^−1^ and 1126–1183 cm^−1^, whereas compounds **7a**–**m** show strong absorptions in the 1372–1339 cm^−1^ and 1182–1167 cm^−1^ regions. In the IR spectra of compounds **6a**–**o*,*** the presence of strong bands in the 1650–1669 cm^−1^ region are attributed to the presence of stretching bonds (C=N) of phthalazine and imidazoline moieties (see Supplementary Materials: **6a**–Figure S8, **7a**–Figure S12, **7k**–Figure S16).

**Scheme 3:**
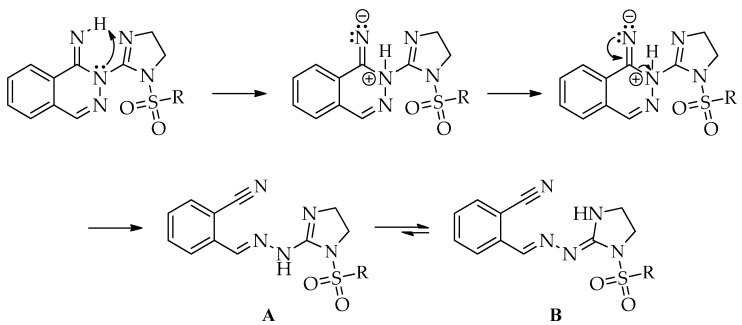
Proposed mechanism for the formation of benzonitriles **7**.

The proposed mechanism of the formation of benzonitriles **7a**–**m** may be explained as follows. The proton shift from the imine group to the nitrogen atom at position 2 of phthalazin-1(2*H*)-imine gives rise to the formation of an unstable monosubstituted sulfonyl intermediate with subsequent opening of the heterocyclic ring (Scheme 3). It was documented that different heterocyclic rings can be opened under mild conditions to form benzonitrile derivatives [42,43].

### 2.2. X-Ray Crystal Structure Determination of ***5***, ***6c***, ***6e***, ***7g***, and ***7k***

Diffraction experiments were carried out at room temperature with an Oxford Diffraction Xcalibur E diffractometer using Mo Kα radiation for **6c**, **6e**, **7g**, and **7k** and with an Oxford Diffraction SuperNova diffractometer using Cu Kα radiation for **5**. Diffraction data were processed with CrysAlisPro software version 1.1.4 [44]. In the case of **7k**, the structure was determined from a specimen twinned by nonmerohedry (twin operation: 180° rotation around [0.00 0.00 1.00] in the reciprocal space). The structures were solved with the program SHELXT [45] and refined by the full-matrix least-squares method on *F*^2^ with SHELXL-2019 [46] within the Olex2 software version 1.5 [47]. Hydrogen atoms were placed in calculated positions and refined as riding on their carriers, except those of the N-H groups in **5** and **7g,** which were freely refined. Crystallographic data and details of structure refinement are collected in Table 1. CCDC 2361137–2361141 contains the supplementary crystallographic data for this paper. These data can be obtained free of charge via http://www.ccdc.cam.ac.uk/ accessed on 27 September 2024 (or from the CCDC, 12 Union Road, Cambridge CB2 1EZ, UK; Fax: +44-1223-336033; E-mail: deposit@ccdc.cam.ac.uk). In Supplementary Materials Figures S20–S24 contain CheckCIF/PLATON reports for compounds: **5**, **6c**, **6e**, **7g**, and **7k**. Figures S25–S27 show crystal packing in **6e**, **6c**, **7g** and **7k** and centrosymmetric dimer via π-π stacking interactions in **6c**.

**Figure 5:**
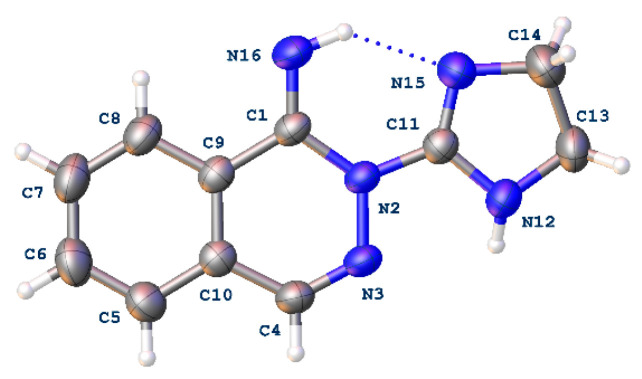
Molecular structure of **5**. Displacement ellipsoids are shown at the 50% probability level.

**Figure 6:**
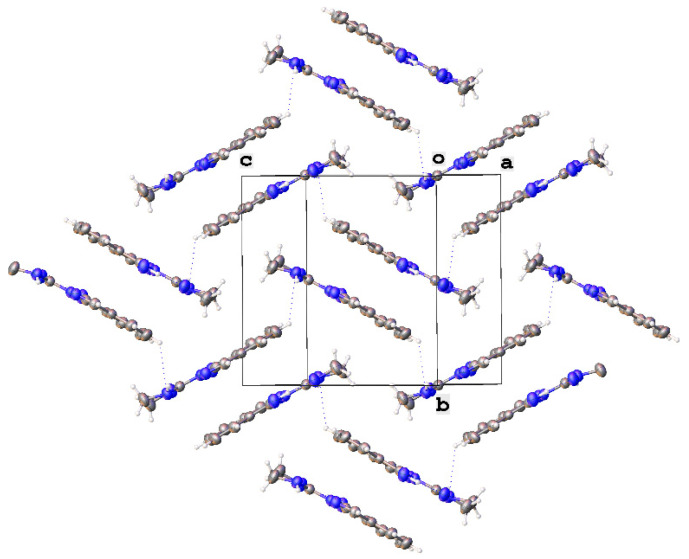
Arrangement of molecules via π–π stacking and C-H···π interactions in **5**.

In crystals, the molecule of **5** adopts a nearly flat structure (Figure 5) as a result of the intramolecular resonance assisted N-H···N hydrogen bond (N16···N15 2.7584(15) Å, H16···N15 1.99(2) Å, <N16-H16···N15 138.9(13)°) and, to some extent, also due to the conjugation between the 1-phthalazinimine group and the amidic part of the imidazoline fragment, what is reflected in the length of N2-C11 bond of 1.4035(13) Å. The five-membered ring of the imidazolidine fragment adopts an envelope form with the C13 atom as a flap. The crystal packing (Figure 6) is mostly determined by π–π stacking interactions between a pair of inversion-related molecules (interplanar distance 3.443 Å) and C-H···π interactions organizing these pairs into layers parallel to the (101) lattice planes. The molecules from the adjacent layers, which are related by the unit translation along (100) form weak N12-H12···N16 hydrogen bonds (N12···N16^i^ 3.1032(14) Å, H12···N16^i^ 2.34(2) Å, <N12-H12···N16^i^ 145.2(12)°; symmetry code i: 1 + x, +y, +z).

**Figure 7:**
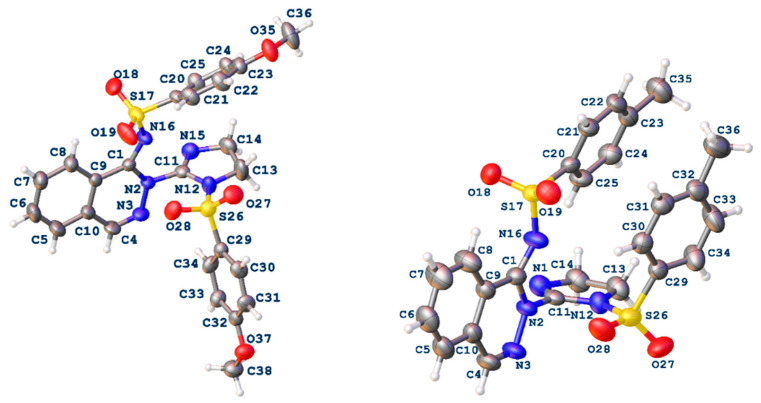
Molecular structure of **6e** (**left**) and **6c** (**right**). Displacement ellipsoids are shown at the 50% probability level.

The attachment of bulky phenylsulfonyl groups to the imino N16 and amino N12 nitrogen atoms results in a significant twist between 1-phthalazinimine and imidazolyl fragments around the N2-C11 bond. As shown by the crystal structures of **6e** and **6c**, these fragments are oriented nearly perpendicular (dihedral angles 82.8 and 90.0°, respectively) with the N2-C11 bond elongated, as compared to **5**, to 1.421(3) Å and 1.425(3) Å in **6e** and **6c**, respectively. In both strongly overcrowded molecules configuration at the C1=N16 bond is *E,* and there is a short intramolecular contact between the phthalazine N2 and sulfonyl O28 atoms (2.726(3) Å in **6e** and 2.766(3) Å in **6c**) that is *c.a.* 0.2 Å shorter than the sum of van der Waals radii. Nevertheless, the overall shape of the molecules differs significantly due to differences in the conformation around S-N and S-C bonds (Figure 7). In **6c**, the molecular conformation is stabilized by stacking interactions between the two benzene rings of the phenylsulfonyl fragments (dihedral angle 20.2°, centroid-to-centroid distance 3.941 Å), whereas in **6e**, these rings are far apart and form a dihedral angle of 79.7°.

**Figure 8:**
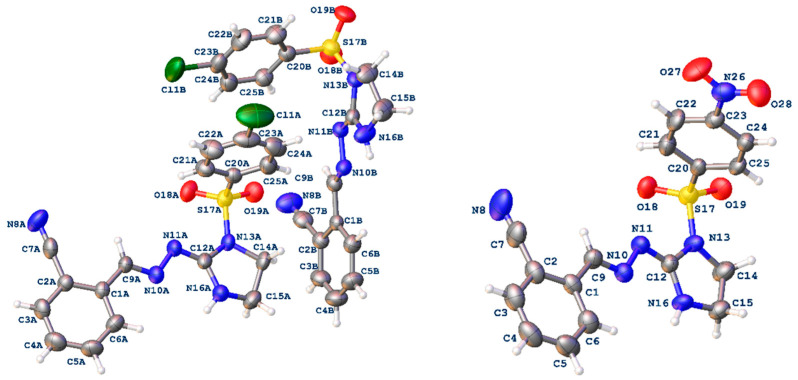
Molecular structure of **7g** (**left**) and **7k** (**right**). Displacement ellipsoids are shown at the 50% probability level.

A series of 2-(benzylidenehydrazinylidene)imidazolidine derivatives is represented by **7g**, which crystallizes with two molecules in the asymmetric unit, and **7k** (Figure 8). Their chemical formulas differ in a substituent at the para position of the phenylsulfonyl group (Cl vs. NO_2_) that is attached to the imidazolidine fragment. The C9=N10 and N11=C12 double bonds show an *E* configuration. In crystals, these three molecules show a similar conformation with a relatively flat 2-(benzylidenehydrazinylidene)imidazolidine part and the benzene ring of the phenylsulfonyl group oriented approximately perpendicular to it (dihedral angles between these planes—80.4, 88.6° for **7g** and 89.9° for **7k**).

### 2.3. Molecular Modeling of Compound ***7k***

Molecules containing heteroatoms display prototropic tautomerism. This common phenomenon is associated with biologically active molecules. According to estimates, part of the drugs sold on the market form at least three tautomers. An understanding of the tautomeric mixture is crucial to determining a drug’s mechanism of action [48,49]. It should be assumed that the dipole moment and hydrophobicity often vary between the tautomers of a given molecule. Therefore, it is crucial to determine all the possible tautomeric forms, as well as their relative stability. 

**Figure 9:**
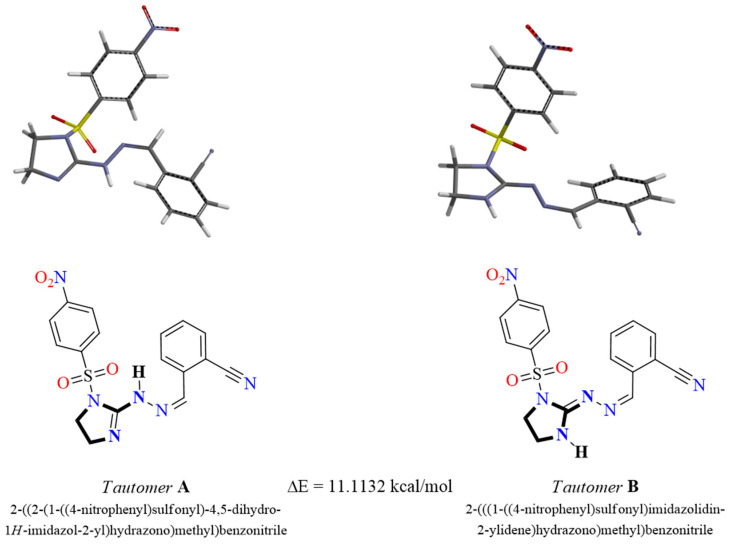
Structures of two possible tautomers (**A**) and (**B**) of compound **7k** and relative energy (ΔE, kcal/mol) calculated in vacuum at B3LYP/6.31G** level of theory.

The tautomeric equilibrium of compound **7k** (R = -C_6_H_4_-NO_2_-*p*) was theoretically evaluated at the ab initio level using the density functional (B3LYP) method with the 6-31G** basis set as implemented into the computer program (Spartan ’14 version 1.1.4) [50]. It could be shown by means of in silico calculations that the tautomeric equilibrium in the obtained benzonitrile 7k lies quantitatively on the side of the iminoimidazolidine form B (Figure 9). The obtained data revealed that tautomer B possessing the torsion angle of hydrazono-methyl fragment (-C_2′(iminoimidazoline)_=N-N=CH-) about *Φ* = 176° was calculated to be slightly lower in energy (E = −1,056,497.8417 kcal/mol) than conformer A (E = −1,056,486.7285 kcal/mol) (ΔE = 11.1132 kcal/mol). The calculated dipole moment of the conformer B (μ = 5.51 debye) would be predicted to exist in a polar solvent such as water. 

**Figure 10:**
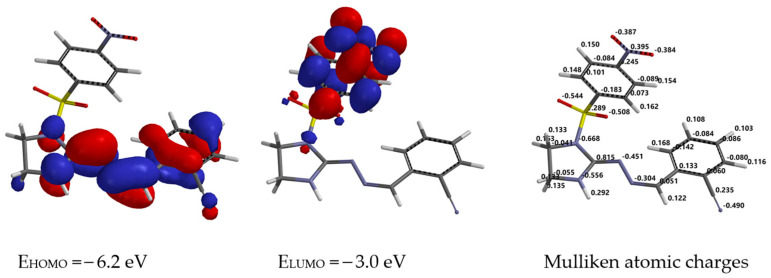
Orbital diagrams of HOMO and LUMO, and Mulliken atomic charge distribution (right) for the optimized structure of tautomer B of compound **7k**.

In order to identify the reactive sites of benzonitriles 7, the electronic structure of representative compound **7k** was studied using ab initio 6-31G** calculations. Figure 10 shows the localization and shape of the highest occupied molecular orbital (HOMO), the lowest unoccupied molecular orbital (LUMO), and Mulliken atomic charge distribution over the atoms of optimized compound **7k** using the DFT method at the B3LYP/6-31G** basis set in the gas phase. 

Frontier molecular orbital HOMO (E_HOMO_ = −6.2 eV) is confined to a carbon atom (C_2′_) and two nitrogen atoms (N_1′_ and N_3′_) of the imidazoline system, nitrogen atoms and a carbon atom forming a methyl-hydrazone motif (=N-N=CH-), and four aromatic carbon atoms. The frontier orbital LUMO (E_LUMO_ = −3.0 eV) is located mostly on the 4-nitrophenyl scaffold. 

The net of atomic charges shown by a Mulliken population analysis has a significant influence on molecule properties. In the compound, the most shielded by an electron cloud are the oxygen atoms of the sulfonyl group, having negative charges of −0.544 and −0.508, and two nitrogen atoms attached to the electrophilic carbon C_2′_ of imidazoline skeleton with calculated negative charges of −0.668 and −0.556, respectively (Figure 10). 

### 2.4. Biological Evaluation

#### 2.4.1. In Vitro Anticancer Evaluation

As part of the known in vitro disease-oriented antitumor screening program, an in vitro cytotoxic activity evaluation was performed against a panel of approximately 60 human cancer cell lines at the National Cancer Institute (NCI, Bethesda, MD, USA). These cell lines were derived from nine types of cancer, such as leukemia, lung, colon, CNS, melanoma, ovarian, renal, prostate, and breast. [51,52]. In the first step, compound 5, sulfonyl derivatives **6a**–**o** and benzonitriles **7a**–**m** were pre-screened at a concentration of 10 μM in approximately sixty cell lines within nine tumor-type subpanels. The data were reported as a mean graph of the percent growth of the treated cells relative to the no-drug control. All of the compounds tested showed no cytotoxic activity, and none were passed to the secondary screening process.

#### 2.4.2. Determination of Antioxidant Activity

It is well known that many pathologies, such as cancer, diabetes, and cardiovascular and neurodegenerative diseases, are caused by the imbalance between reactive oxygen species (ROS) and the antioxidant system [53]. For example, antioxidants are being studied in order to develop more effective treatments for neurodegeneration [54], chronic hepatitis C, and steatohepatitis [55].

The free radical scavenging ability of synthesized compounds **6a**–**o** and **7a**–**m** was analyzed with colorimetric methods: ABTS (2,2′-azinobis(3-ethylbenzothiazoline-6-sulphonic acid) and DPPH (2,2′-diphenyl-1-picrylhydrazyl). Moreover, the reduction ability test (FRAP, Ferric ion reducing antioxidant power test) and β˗carotene bleaching assay—assessment of lipid peroxidation—were used for in vitro biological potency of compounds **6a**–**o** and **7a**–**m**. These tests are simple colorimetric methods that are capable of providing repeatable results. The data are expressed as sufficient concentration to obtain 50% of the maximum scavenging activity (IC_50_). The IC_50_ values are shown in Table 2. Ascorbic acid was used as a reference compound.

From our in vitro results, most of the compounds showed no or low activity in applied assays. In most cases, they have not reached IC_50_ values despite increasing concentrations until 2 mg/mL—the higher concentrations resulted in precipitation. Only four compounds exhibited moderate to very low scavenging ability in the β˗carotene bleaching test compared with ascorbic acid (IC_50_ = 86.21–97.41 µg/mL vs. IC_50_ = 49.76 µg/mL). It should be pointed out that scavenging activity on the β˗carotene bleaching assay was exhibited by sulfonamides **6n** and **6o** that possess bulky and electron-rich substituents: biphenyl and phenoxyphenyl moieties. 

The highest ABTS antiradical activity was found for sulfonamide **6j** containing bromophenyl substituent (R = 4-Br-C_6_H_5_, IC_50_ = 52.77 µg/mL). This compound was observed to display slightly weaker activity (IC_50_ = 95.24 µg/mL) in the β˗carotene bleaching assay. On the other hand, benzonitrile **7i** bearing two chlorine atoms on a phenyl ring system showed only activity in the β˗carotene bleaching test (IC_50_ = 86.21 µg/mL) and a significant decrease in potency in ABTS assay (IC_50_ = 391.24 µg/mL). A further decrease in radical scavenging capability was observed for compounds containing electron-donating substituents on the phenyl ring—**6c**, **6d**, **6e**, **6f**, **6g**, **7e**, e.g., -CH_3_, -CH_3_O, -C(CH_3_)_3_ (IC_50_ = 338.63–682.71 µg/mL). This observation suggests that the presence of electron-withdrawing groups (e.g., bromine or chlorine atoms) may facilitate potency and leads to improved anti-radical ability.

On the other hand, in the DDPH and FRAP assays, most tested compounds displayed weak or no antioxidant capacity with IC_50_ values in the range from 219.92 µg/mL to 891.22 µg/mL (see Table 2).

#### 2.4.3. In Silico Evaluation of Free Radical Scavenging Capacity

Considering the results of antioxidant studies of the tested compounds, a theoretical study on antioxidant activity for derivatives that exhibited antioxidant potency (**6j**, **6k**, **6n**, **6o**, **7h**, and **7i**) has also been performed using Spartan ’14 version 1.1.4 to obtain the energies of the highest occupied molecular orbital (HOMO), a level of energy below the highest occupied molecular orbital (HOMO{−1}), lowest unoccupied molecular orbital (LUMO) and a level of energy above the lowest unoccupied molecular orbital (LUMO{+1}).

As a fundamental concept in chemistry, molecular orbital (MO) theory is extensively used to describe the behavior of molecules. This theory is useful not only for explaining chemical behavior, such as reactivity, but also for providing a conceptual construct needed to describe phenomena involving molecular electronic structures. In this term, frontier orbitals offer information about the energy of the Highest Occupied Molecular Orbital (HOMO) and the Lowest Unoccupied Molecular Orbital (LUMO). The higher HOMO energy level means the greater the ability to realize nucleophilic attacks or donate electrons. On the other hand, the lower LUMO energy level equals the higher ability to accept electrons during an electrophilic attack. As a consequence, these crucial parameters describe antioxidant ability because electron transfer reactions can be influenced by them. It is assumed that molecules with large HOMO–LUMO gaps (or higher GAP values) may be regarded as more stable and inert, while those with small gaps (or lower GAP values) are generally reactive. In general, compounds with large gaps are thermodynamically stable, whereas compounds with small gaps are likely to undergo an easier electronic transition. Therefore, a small gap indicates good free scavenging activity of the molecule [56,57]. These different energy gaps may be calculated for the above-mentioned four orbitals by the following equations: GAP^1^ = (LUMO) − (HOMO); GAP^2^ = (LUMO{+1}) − (HOMO); GAP^3^ = (LUMO) − (HOMO{−1}); GAP^4^ = (LUMO{+1}) − (HOMO{−1}). Compounds that display a potent antioxidant activity donate electrons easily and become stable in this process. Such molecules rearrange electrons after donation to prevent them from remaining in reactive radical forms [58,59]

Table 3 summarizes the data obtained for the computed energy levels of molecular orbitals and GAP values (GAP^1^, GAP^2^, GAP^3^, and GAP^4^), which correspond to energy variation between frontier orbitals and between all the energy states. 

The calculated HOMO values and the high GAP values of compounds **6j**, **6k**, **6n**, **6o**, **7h**, and **7i** that range from 10.0 eV to 11.6 eV show their low reactivity and consequently higher stability. This is in agreement with the in vitro tests and may confirm their low ROS scavenging activity.

#### 2.4.4. In Vitro Inhibition of Receptor for Advanced Glycation End-Products (RAGE)

The formation of advanced glycation end-products (AGEs) is caused by nonenzymatic reactions between reducing sugars and amino groups in amino acids, proteins, and nucleic acids, followed by oxidative degradation. In spite of the fact that AGEs are multifactorial in nature, recent research has suggested that they trigger a cascade of signaling events, which results in the formation of pro-inflammatory cytokines that trigger additional oxidative stress and the accumulation of advanced glycation end-products being a key factor in their pathogenesis [60].

In 1992, Neeper et al. described RAGE (receptor for advanced glycation end-products), also known as AGER [61]. RAGE is able to bind advanced glycation end-products (AGEs), which largely consist of glycoproteins that have been modified non-enzymatically through the Maillard reaction [62]. It is assumed that numerous chronic diseases may be associated with RAGE activation. During pathogenesis, the binding of RAGE agonist ligands leads to the activation of nuclear factor kappa B (NF-κB) signaling pathways, which controls several genes involved in inflammation [63].

Recent attention has been paid to the interaction between the RAGE and AGE pathways when it comes to the development of therapeutics to treat AGE-related diseases such as diabetes, atherosclerosis, cancers, and neurodegenerative diseases or obesity. On the other hand, despite extensive research into drugs targeting RAGE, their development has met with limited success [64,65,66,67].

The impact of the selected phthalazine derivatives **6a**, **6b**, **6g**, and **6m**, and hydrazonomethylbenzonitriles **7a**, **7c**–**g**, and **7i**–**k** on the AGE2-BSA/sRAGE (soluble RAGE) interaction was determined by ELISA assay (Table 4). In the series of phthalazine-sulfonamides **6a**, **6b**, **6g**, and **6m**, only compound **6b** displayed a weak inhibition of the AGE2-BSA/sRAGE interaction. In turn, among hydrazonomethylbenzonitrile derivatives, moderate inhibitory properties were detected for compound **7d** with an electron-donating methoxy group at position 3 of the phenyl ring (R = 3-CH_3_O-C_6_H_4_) or an electron-withdrawing chlorine atom at position 2 (compound **7f**, R = 2-Cl-C_6_H_4_) or nitro substituent at position 4 of the phenyl ring (compound **7k**, R = 4-NO_2_-C_6_H_4_) (inhibition in the range of 31.9–36.7%). Interestingly, compounds with different substitution patterns—that possess a methyl (compound **7c**) or a bulky *tert*-butyl group (compound **7e**) as well as a chlorine atom (compound **7g**) at position 4 or two chlorine atoms at positions 2 and 6 or 3, and 4 of the aryl moiety (compounds **7i** and **7j**)—were found to be inactive. The obtained results indicate that the electronic or steric effects of the type of substituent may affect the biological properties of the tested compounds.

## 3. Materials and Methods

### 3.1. Chemistry

#### 3.1.1. General Methods and Physical Measurements

All reagents and solvents were purchased from commercial sources and used without further purification.

The melting points were determined with BÜCHI Melting point B-545 (BÜCHI Labortechnik, Switzerland) and Boëtius apparatus (Boetius micromelting point apparatus, VEB Wägetechnik, Germany) and are uncorrected.

The infrared spectra were recorded on a Nicolet 380 FT-IR spectrophotometer (Thermo Fisher Scientific Inc., Waltham, MA, USA).

The elemental analyses of carbon, hydrogen, nitrogen, and sulfur determined for compounds were within ±0.4% of the theoretical values.

The ^1^H-NMR and ^13^C-NMR spectra of compounds were registered at 20–22 °C on a Varian Inova 500 MHz spectrometer (^1^H = 500 MHz, ^13^C = 125 MHz) and Bruker Advance III HD 400 MHz spectrometer (^1^H = 400 MHz, ^13^C = 100 MHz) with a BBFO ^31^P-^15^N probe and a TXI ^1^H/^13^C/^31^P Mercury-VX three-channel probe in deuterated dimethyl sulfoxide (DMSO-*d*_6_) or dimethylformamide (DMF-*d*_7_). Solvent signals were the internal standard. The values of chemical shifts are given in *ppm and* coupling constants (*J*) are expressed in hertz (*Hz*).

Mass spectra were recorded on an LCMS 2010 spectrometer (Shimadzu, Tokyo, Japan). The compounds were identified based on their molecular ions obtained through electrospray ionization.

Compounds were purified by the use of preparative chromatography or crystallization. Thin-layer chromatography was performed on silica gel plates with fluorescence detection (Merck Silica Gel 254, Merck KGaA, Darmstadt, Germany). After drying, spots were detected under UV light (λ = 254 nm). 

The measured C, H, and N elemental analyses were within 0.4% of calculated values. 

Stability studies of copper(II) complexes were performed by means of UV–Vis spectrophotometer Perkin-Elmer Lambda UV/VIS (Evolution 300 UV–Vis, Thermo Electron Scientific Instruments Llc, Madison, WI, USA) connected to a personal computer running the VISION pro software vision version 4.4.1., math version 24.00).

Molecular modeling studies were performed at ab initio level using the density functional method (B3LYP) with the 6-31G** basis set as implemented into Spartan ’14 version 1.1.4 [50].

#### 3.1.2. Synthesis of (2-(4,5-dihydro-1*H*-imidazol-1-ium-2-yl)-1,2-dihydrophthalazin-1-yl)amino Sulfate (**4**) and 2-(4,5-dihydro-1*H*-imidazol-2-yl)phthalazin-1(2*H*)-imine (**5**)

Step 1

To a suspension of compound **3** (2.162 g, 10 mmol) [41] in 3 mL of anhydrous dimethylformamide, hydroxylamine-*O*-sulfonic acid (4.524 g, 40 mmol) was added gradually over 15 min. at room temperature (20–22 °C). After 1 h, a solid precipitated out, and the stirring continued for 24 h. Then, 10–20 g of crushed ice was added, and the suspension was filtered off and washed with water. The obtained intermediate **4** was used for the next step without further purification. Thus, crude product **4** was dried, 3.03 g, yield 97%, white powder, m.p. 185–189 °C, IR (KBr) *v* [cm^−1^]: 3297, 3219, 3083, 3043, 2919, 1629, 1595, 1569, 1417, 1266, 1231, 1055, 1068, 838, 775, 757, 674, 606, 573, 549.

Step 2

To a suspension of crude betaine **4** (3.113 g; 10 mmol) in 10 mL of water, 10 mL of 10% NaOH was added at room temperature (20–22 °C). A precipitate was formed after 1 min. of stirring. The mixture was stirred for 1 h, cooled, filtered off, washed with cold water and white solid was dried, 1.9 g, yield 89%, crystallized from ethanol, white powder, m.p. 171–175 °C; IR (KBr) *v* [cm^−1^]: 3310, 3157, 2957, 2851, 1639, 1606, 1570, 1489, 1459, 1447, 1390, 1332, 1312, 1281, 1115, 997, 911, 761, 589; ^1^H-NMR (DMSO-*d*_6_, 500 MHz) *δ* [ppm]: 3.33 (m, 2H, CH_2_), 3.89 (m, 2H, CH_2_), 6.94 (br. s, 1H, NH), 7.69–7.73 (m, 3H, 3xCH-Ar), 8.11 (s, 1H, CH-Ar), 8.34–8.36 (m, 1H, CH-Ar), 10.87 (br. s, 1H, NH); ^13^C-NMR (DMSO-*d*_6_, 125 MHz) *δ* [ppm]: 42.9, 54.2, 125.2, 125.8, 127.3, 129.9, 132.5, 132.7, 138.3, 147.9, 161.7; MS (ESI, CH_3_OH:CH_3_CN containing 0.1% CH_3_COOH, 1:1, *v*/*v*): *m*/*z* = 214 [M+H]^+^. Anal. calcd for C_11_H_11_N_5_ (213.24 g/mol): C, 61.96; H, 5.20; N, 32.84. Found: C, 61.88; H, 5.17; N, 32.88%.

#### 3.1.3. Synthesis of *N*-(2-(1-(aryl(alkyl)sulfonyl)-4,5-dihydro-1*H*-imidazol-2-yl)phthalazin-1(2*H*)-ylidene)aryl(alkyl)sulfonamides **6a**–**o** and 2-(((1-(aryl(alkyl)sulfonyl)imidazolidin-2-ylidene)hydrazono)methyl)benzonitriles **7a**–**n** (General Procedure)

To the solution of 2-(4,5-dihydro-1*H*-imidazol-2-yl)phthalazin-1(2*H*)-imine (**5**) (0.213 g, 1 mmol) in anhydrous dichloroethane (5 mL), triethylamine (0.405 g, 0.556 mL, d = 0.726 g/mL, 4 mmol) and the appropriate sulfonic acid chloride were added (2 mmol). The mixture was heated at 90 °C for 8–12 h. The course of the reaction was monitored by thin-layer chromatography (eluent: chloroform:ethyl acetate, 4:1, *v*/*v*). Then, the mixture was evaporated under reduced pressure, and the oily residue was dissolved in 10–15 mL of chloroform, dried over anhydrous MgSO_4_, and filtered off. Products **6a**–**o** and **7a**–**n** were isolated by preparative thin-layer chromatography (chromatotron), using as eluent: dichloromethane or mixtures of dichloromethane and ethyl acetate (95:5, 9:1 or 4:1, *v*/*v*). According to the above procedure, the following compounds were obtained:

*N*-(2-(1-(methylsulfonyl)-4,5-dihydro-1*H*-imidazol-2-yl)phthalazin-1(2*H*)-ylidene)methanesulfonamide (**6a**)

Starting from 0.229 g of methanesulfonyl chloride (0.155 mL, d = 1.48 g/mL), yield 0.13 g (36%), eluent: CH_3_COOC_2_H_5_:CH_2_Cl_2_:CH_3_OH, 8:1:1, *v*/*v*/*v*, white powder, white powder, m.p. 201–202 °C; IR (KBr) *v* [cm^−1^]: 3126, 3008, 2990, 2937, 2917, 1669, 1611, 1564, 1359, 1274, 1160, 1126, 986, 932, 832, 598, 533; ^1^H-NMR (DMSO-*d*_6_, 400 MHz) *δ* [ppm]: 3.17 (s, 3H, CH_3_), 3.35 (s, 3H, CH_3_), 4.04–4.09 (m, 2H, CH_2_), 4.15–4.20 (m, 2H, CH_2_), 8.10–8.12 (m, 1H, CH-Ar), 8.16–8.17 (m, 2H, 2xCH-Ar), 8.84 (s, 1H, CH-Ar), 8.99 (dd, *J*_1_ = 0.9 Hz, *J*_2_ = 8.3 Hz, 1H, CH-Ar); ^13^C-NMR (DMSO-*d*_6_, 100 MHz) *δ* [ppm]: 39.2, 44.9, 48.6, 51.2, 123.8, 128.9, 129.2, 130.7, 133.6, 136.4, 142.7, 149.3, 151.8; MS (ESI, CH_3_OH:CH_3_CN containing 0.1% CH_3_COOH, 1:1, *v*/*v*): *m*/*z* = 392 [M+Na]^+^ and *m*/*z* = 433 [M+Na+CH_3_CN]^+^. Anal. calcd for C_13_H_15_N_5_O_4_S_2_ (369.42 g/mol): C, 42.27; H, 4.09; N, 18.96; S, 17.36. Found: C, 42.21; H, 3.89; N, 18.78; S, 17.22%.

*N*-(2-(1-(phenylsulfonyl)-4,5-dihydro-1*H*-imidazol-2-yl)phthalazin-1(2*H*)-ylidene)benzenesulfonamide (**6b**)

Starting from 0.353 g of benzene-1-sulfonyl chloride (0.255 mL, d = 1.384 g/mL), yield 0.16 g (32%), crystallized from methanol, white powder, m.p. 202–204 °C; IR (KBr) *v* [cm^−1^]: 3117, 3088, 3066, 2933, 2882, 1658, 1608, 1547, 1446, 1371, 1296, 1144, 1085, 726, 619, 568; ^1^H-NMR (DMSO-*d*_6_, 400 MHz) *δ* [ppm]: 3.77 (br.s, 4H, CH_2_-CH_2_), 7.55–7.62 (m, 5H, 5xCH-Ar), 7.71–7.73 (m, 1H, CH-Ar), 7.82–7.85 (m, 4H, 4xCH-Ar), 8.21–8.27 (m, 3H, 3xCH-Ar), 8.97 (s, 1H, CH-Ar), 9.18–9.21 (m, 1H, CH-Ar); ^13^C-NMR (DMSO-*d*_6_, 100 MHz) *δ* [ppm]: 48.3, 51.1, 123.9, 125.8 (two overlapping signals), 127.9 (two overlapping signals), 129.2, 129.2 (two overlapping signals), 129.3, 130.1 (two overlapping signals), 130.6, 132.3, 134.0, 134.8, 136.8, 137.1, 143.3, 144.4, 149.1, 152.4. MS (ESI, CH_3_OH:CH_3_CN containing 0.1% CH_3_COOH, 1:1, *v*/*v*): *m*/*z* = 516 [M+Na]^+^. Anal. calcd for C_23_H_19_N_5_O_4_S_2_ (493.56 g/mol): C, 55.97; H, 3.88; N, 14.19; S, 12.99. Found: C, 55.88; H, 3.63; N, 14.32; S, 13.12%.

4-methyl-*N*-(2-(1-tosyl-4,5-dihydro-1*H*-imidazol-2-yl)phthalazin-1(2*H*)-ylidene)benzenesulfonamide (**6c**)

Starting from 0.381 g of 4-methylbenzene-1-sulfonyl chloride, yield 0.108 g (21%), crystallized from methanol, white powder, m.p. 207–210 °C; IR (KBr) *v* [cm^−1^]: 3131, 3054, 2921, 1655, 1609, 1545, 1373, 1276, 1170, 1139, 1086, 829, 668, 603, 565; ^1^H-NMR (DMSO-*d_6_*, 400 MHz) *δ* [ppm]: 2.37 (s, 3H, CH_3_), 2.38 (s, 3H, CH_3_), 3.81 (br.s, 4H, CH_2_-CH_2_), 7.33–7.38 (m, 4H, 4xCH-Ar), 7.69–7.71 (m, 4H, 4xCH-Ar), 8.24–8.25 (m, 3H, 3xCH-Ar), 8.95 (s, 1H, CH-Ar), 9.19–9.22 (m, 1H, CH-Ar); ^13^C-NMR (DMSO-*d*_6_, 100 MHz) *δ* [ppm]: 21.4, 21.5, 48.3, 51.1, 123.9, 125.8 (two overlapping signals), 128.0 (two overlapping signals), 129.2, 129.3, 129.5 (two overlapping signals); 130.5 (two overlapping signals), 130.7, 133.9, 134.1, 136.7, 141.8, 142.4, 143.1, 145.4, 149.3, 152.2; MS (ESI, CH_3_OH:CH_3_CN + 0.1% CH_3_COOH, 1:1, *v*/*v*): *m*/*z* = 544 [M+Na]^+^. Anal. calcd for C_25_H_23_N_5_O_4_S_2_ (521.61 g/mol): C, 57.57; H, 4.44; N, 13.43; S, 12.29. Found: C, 57.62; H, 4.51; N, 13.31; S, 12.13%.

3-methoxy-*N*-(2-(1-((3-methoxyphenyl)sulfonyl)-4,5-dihydro-1*H*-imidazol-2-yl)phthalazin-1(2*H*)-ylidene)benzenesulfonamide (**6d**)

Starting from 0.381 g of 3-methoxybenzene-1-sulfonyl chloride, yield 0.21 g (38%), eluent: CH_2_Cl_2_:CH_3_COOC_2_H_5_, 95:5, *v*/*v*, crystallized from methanol, white powder, m.p. 183–185 °C; IR (KBr) *v* [cm^−1^]: 3107, 3075, 3001, 2922, 2854, 1654, 1598, 1542, 1364, 1293, 1250, 1168, 1143, 1092, 1031, 820, 705, 624, 594; ^1^H-NMR (DMSO-*d*_6_, 400 MHz) *δ* [ppm]: 3.84 (br.s, 10H, 2xOCH_3_+CH_2_-CH_2_), 7.19–7.21 (m, 1H, CH-Ar), 7.29–7.31 (m, 2H, 2xCH-Ar), 7.34–7.35 (m, 1H, CH-Ar), 7.41–7.45 (m, 2H, 2xCH-Ar), 7.51 (t, 2H, 2xCH-Ar), 8.19–8.27 (m, 3H, 3xCH-Ar), 8.99 (s, 1H, CH-Ar), 9.15–9.17 (m, 1H, CH-Ar); ^13^C-NMR (DMSO-*d*_6_, 100 MHz) *δ* [ppm]: 48.4, 51.1, 56.1, 56.2, 111.5, 112.3, 117.7, 117.9, 120.1, 121.0, 123.9, 129.2, 129.3, 130.5, 130.5, 131.3, 134.0, 136.8, 138.2, 143.3, 145.6, 149.2, 152.3, 159.6, 160.0; MS (ESI, CH_3_OH:CH_3_CN containing 0.1% CH_3_COOH, 1:1, *v*/*v*): *m*/*z* = 576 [M+Na]^+^ and *m*/*z* = 576 [M+Na+CH_3_CN]^+^. Anal. calcd for. C_25_H_23_N_5_O_6_S_2_ (553.61 g/mol): C, 54.24; H, 4.19; N, 12.65; S, 11.58. Found: C, 54.34; H, 4.08; N, 12.85; S, 11.47%.

4-methoxy-*N*-(2-(1-((4-methoxyphenyl)sulfonyl)-4,5-dihydro-1*H*-imidazol-2-yl)phthalazin-1(2*H*)-ylidene)benzenesulfonamide (**6e**)

Starting from 0.413 g of 4-methoxybenzene-1-sulfonyl chloride, yield 0.16 g (29%), eluent: CHCl_3_:CH_3_COOC_2_H_5_:(CH_3_)_2_CO, 8:1:1, *v*/*v*/*v*, crystallized from methanol, white powder, m.p. 200–202 °C; IR (KBr) *v* [cm^−1^]: 3136, 3059, 2972, 2938, 2895, 2839, 1650, 1595, 1541, 1499, 1376, 1277, 1254, 1165, 1136, 1084, 1030, 830, 699, 604, 574; ^1^H-NMR (DMSO-*d*_6_, 400 MHz) *δ* [ppm]: 3.84 (br.s, 10H, 2xOCH_3_+CH_2_-CH_2_), 7.03 (d, *J* = 8.9 Hz, 2H, CH-Ar), 7.09 (d, *J* = 8.9 Hz, 2H, CH-Ar), 7.75 (d, *J* = 8.9 Hz, 2H, CH-Ar), 7.78 (d, *J* = 8.9 Hz, 2H, CH-Ar), 8.20–8.24 (m, 3H, CH-Ar), 8.93 (s, 1H, CH-Ar), 9.21 (dd, *J*_1_ = 1.1 Hz, *J*_2_ = 8.1 Hz, 1H, CH-Ar); ^13^C-NMR (DMSO-*d*_6_, 100 MHz) *δ* [ppm]: 48.2, 51.1, 56.1, 56.3, 114.2 (two overlapping signals), 115.2 (two overlapping signals), 123.9, 127.9 (two overlapping signals), 128.3, 129.1, 129.3, 130.4, 130.7 (two overlapping signals), 133.9, 136.5, 136.6, 143.0, 149.4, 152.1, 162.1, 163.9; MS (ESI, CH_3_OH:CH_3_CN containing 0.1% CH_3_COOH, 1:1, *v*/*v*): *m*/*z* = 576 [M+Na]^+^ and *m*/*z* = 617 [M+Na+MeCN]^+^. Anal. calcd for C_25_H_23_N_5_O_6_S_2_ (553.61 g/mol): C, 54.24; H, 4.19; N, 12.65; S, 11.58. Found: C, 54.18; H, 4.23; N, 12.71; S, 11.47%.

*N*-(2-(1-(mesitylsulfonyl)-4,5-dihydro-1*H*-imidazol-2-yl)phthalazin-1(2*H*)-ylidene)-2,4,6-trimethylbenzenesulfonamide (**6f**)

Starting from 0.436 g of 2,4,6-trimethylbenzene-1-sulfonyl chloride, yield 0.237 g (41%), white crystals, m.p. 123–126 °C; IR (KBr) *v* [cm^−1^]: 3129, 3025, 2974, 2938, 2873, 1662, 1606, 1556, 1333, 1291, 1162, 1138, 819, 692, 665, 583; ^1^H-NMR (DMSO-*d*_6_, 400 MHz) *δ* [ppm]: 2.13 (s, 3H, CH_3_), 2.28 (s, 3H, CH_3_), 2.33 (s, 6H, 2xCH_3_), 2.58 (s, 6H, 2xCH_3_), 3.52–3.66 (m, 4H, CH_2_-CH_2_), 6.83 (s, 2H, 2xCH-Ar), 7.02 (s, 2H, 2xCH-Ar), 8.06–8.10 (m, 2H, 2xCH-Ar), 8.14–8.16 (m, 1H, CH-Ar), 8.63 (s, 1H, CH-Ar), 8.97–8.99 (m, 1H, CH-Ar); ^13^C-NMR (DMSO-*d*_6_, 100 MHz) *δ* [ppm]: 20.9 (two overlapping signals), 22.4 (two overlapping signals), 22.6 (two overlapping signals), 48.0, 51.2, 123.7, 128.5, 129.0, 130.9, 131.4 (two overlapping signals), 131.5, 132.4 (two overlapping signals), 133.1, 136.3, 137.9 (two overlapping signals), 138.5, 140.1 (two overlapping signals), 141.0, 142.2, 144.2, 149.9, 151.3; MS (ESI, CH_3_OH:CH_3_CN containing 0.1% CH_3_COOH, 1:1, *v*/*v*): *m*/*z* = 578 [M+H]^+^ and *m*/*z* = 600 [M+Na]^+^. Anal. calcd for C_29_H_31_N_5_O_4_S_2_ (577.72 g/mol): C, 60.29; H, 5.41; N, 12.12; S, 11.10. Found: C, 60.32; H, 5.38; N, 12.14; S, 11.06%.

4-(tert-butyl)-*N*-(2-(1-((4-(tert-butyl)phenyl)sulfonyl)-4,5-dihydro-1*H*-imidazol-2-yl)phthalazin-1(2*H*)-ylidene)benzenesulfonamide (**6g**)

Starting from 0.466 g of 4-(*tert*-butyl)benzene-1-sulfonyl chloride, yield 0.14 g (23%), eluent: CH_2_Cl_2_ and CH_2_Cl_2_:CH_3_COOC_2_H_5_, 20:5, *v*/*v*, white crystals, m.p. 219–222 °C; IR (KBr) *v* [cm^−1^]: 3126, 3066, 2964, 2904, 2871, 1656, 1612, 1555, 1371, 1285, 1173, 1152, 1106, 1086, 933, 828, 640, 621, 596; ^1^H-NMR (DMSO-*d_6_*, 400 MHz) *δ* [ppm]: 1.26 (s, 9H, C(CH_3_)_3_), 1.30 (s, 9H, C(CH_3_)_3_), 3.71 (br.s, 2H, CH_2_), 3.89 (br.s, 2H, CH_2_), 7.54–7.59 (m, 4H, 4xCH-Ar), 7.71–7.77 (m, 4H, 4xCH-Ar), 8.21–8.26 (m, 3H, 3xCH-Ar), 8.95 (s, 1H, CH-Ar), 9.20 (dd, *J*_1_ = 1.1 Hz, *J*_2_ = 7.9 Hz, 1H, CH-Ar); ^13^C-NMR (DMSO-*d*_6_, 100 MHz) *δ* [ppm]: 31.1 (three overlapping signals), 31.3 (three overlapping signals), 35.2, 35.5, 48.2, 51.1, 123.9, 125.6 (two overlapping signals), 125.9 (two overlapping signals), 126.9 (two overlapping signals), 128.0 (two overlapping signals), 129.2, 129.3, 130.7, 133.9, 134.2, 136.7, 141.7, 143.1, 149.2, 152.2, 155.3, 157,8. MS (ESI, CH_3_OH:CH_3_CN containing 0.1% CH_3_COOH, 1:1, *v*/*v*): *m*/*z* = 628 [M+Na]^+^ and *m*/*z* = 669 [M+Na+CH_3_CN]^+^. Anal. calcd for C_31_H_35_N_5_O_4_S_2_ (605.77 g/mol): C, 61.46; H, 5.82; N, 11.56; S, 10.59. Found: C, 61.39; H, 5.92; N, 11.51; S, 10.39%.

2-chloro-*N*-(2-(1-((2-chlorophenyl)sulfonyl)-4,5-dihydro-1*H*-imidazol-2-yl)phthalazin-1(2*H*)-ylidene)benzenesulfonamide (**6h**)

Starting from 0.422 g of 2-chlorobenzene-1-sulfonyl chloride, yield 0.15 g (27%), white/beige powder, m.p. 239–242 °C; IR (KBr) *v* [cm^−1^]: 3098, 3060, 2987, 2928, 2879, 2851, 1668, 1608, 1545, 1454, 1352, 1302, 1172, 1149, 1108, 1043, 756, 597; ^1^H-NMR (DMSO-*d*_6_, 400 MHz) *δ* [ppm]: 3.92 (br.s, 4H, CH_2_-CH_2_), 7.29–7.33 (m, 1H, CH-Ar), 7.57–7.60 (m, 2H, CH-Ar+NH), 7.63–7.72 (m, 4H, 4xCH-Ar), 8.08 (dd, *J*_1_ = 1.6 Hz, *J*_2_ = 7.8 Hz, 1H, CH-Ar), 8.13–8.23 (m, 3H, 3xCH-Ar), 8.71 (s, 1H, CH-Ar), 8.94–8.97 (m, 1H, CH-Ar); ^13^C-NMR (DMSO-*d*_6_, 100 MHz) *δ* [ppm]: 49.5, 51.3, 123.9, 127.6, 128.2, 129.0 (two overlapping signals), 129.3, 130.4, 130.9, 131.3, 131.7, 132.0, 132.9, 133.9, 133.9, 135.9, 136.0, 136.8, 141.0, 142.9, 148.6, 152.0; MS (ESI, CH_3_OH:CH_3_CN containing 0.1% CH_3_COOH, 1:1, *v*/*v*): *m*/*z* = 584, 586, and 588 [M+Na]^+^. Anal. calcd for C_23_H_17_Cl_2_N_5_O_4_S_2_ (562.45 g/mol): C, 49.11; H, 3.05; N, 12.45; S, 11.40. Found: C, 49.08; H, 3.12; N, 12.56; S, 11.46%.

4-chloro-*N*-(2-(1-((4-chlorophenyl)sulfonyl)-4,5-dihydro-1*H*-imidazol-2-yl)phthalazin-1(2*H*)-ylidene)benzenesulfonamide (**6i**)

Starting from 0.422 g of 4-chlorobenzene-1-sulfonyl chloride, yield 0.206 g (37%), white powder, m.p. 238–242 °C; IR (KBr) *v* [cm^−1^]: 3136, 3084, 2965, 2925, 2854, 1662, 1605, 1536, 1376, 1272, 1181, 1088, 831, 756, 638; ^1^H-NMR (DMSO-*d*_6_, 400 MHz) *δ* [ppm]: 3.90 (br.s, 4H, CH_2_-CH_2_), 7.59–7.62 (m, 2H, 2xCH-Ar), 7.65–7.68 (m, 2H, 2xCH-Ar), 7.77–7.81 (m, 4H, 4xCH-Ar), 8.25–8.28 (m, 3H, 3xCH-Ar), 8.99 (s, 1H, CH-Ar), 9.14–9.16 (m, 1H, CH-Ar); ^13^C-NMR (DMSO-*d*_6_, 100 MHz) *δ* [ppm]: 48.5, 51.2, 123.8, 127.7 (two overlapping signals), 129.3 (two overlapping signals), 129.4 (two overlapping signals), 129.8 (two overlapping signals), 130.19 (two overlapping signals), 130.5, 134.2, 135.8, 137.0, 137.1, 139.8, 143.2, 143.6, 148.8, 152.5; MS (ESI, CH_3_OH:CH_3_CN containing 0.1% CH_3_COOH, 1:1, *v*/*v*): *m/z* = 584, 586, and 588 [M+Na]^+^ and *m*/*z* = 625, 627, and 629 [M+Na+CH_3_CN]^+^. Anal. calcd for C_23_H_17_Cl_2_N_5_O_4_S_2_ (562.45 g/mol): C, 49.11; H, 3.05; N, 12.45; S, 11.40. Found: C, 49.07; H, 3.16; N, 12.49; S, 11.56%.

4-bromo-*N*-(2-(1-((4-bromophenyl)sulfonyl)-4,5-dihydro-1*H*-imidazol-2-yl)phthalazin-1(2*H*)-ylidene)benzenesulfonamide (**6j**)

Starting from 0.511 g of 4-bromobenzene-1-sulfonyl chloride, yield 0.12 g (18%), eluent: CHCl_3_:CH_3_COOC_2_H_5_:(CH_3_)_2_CO, 8:1:1, *v*/*v*/*v*, crystallized from methanol, white/beige powder, m.p. 239–244 °C; IR (KBr) *v* [cm^−1^]: 3135, 3084, 3058, 2956, 2893, 1668, 1605, 1573, 1539, 1377, 1270, 1255, 1182, 1139, 1083, 829, 744, 631; ^1^H-NMR (DMSO-*d*_6_, 400 MHz) *δ* [ppm]: 3.90 (m, 4H, CH_2_-CH_2_), 7.69–7.75 (m, 6H, 6xCH-Ar), 7.80–7.82 (m, 2H, 2xCH-Ar), 8.22–8.28 (m, 3H, 3xCH-Ar), 8.99 (s, 1H, CH-Ar), 9.14–9.16 (m, 1H, CH-Ar); ^13^C-NMR (DMSO-*d*_6_, 100 MHz) *δ* [ppm]: 48.5, 51.2, 123.8, 126.0, 127.8 (three overlapping signals), 128.9, 129.3, 129.8 (two overlapping signals), 130.5, 132.3 (three overlapping signals), 133.1 (two overlapping signals), 134.2, 136.2, 137.0, 143.6, 148.8, 152.5; MS (ESI, CH_3_OH:CH_3_CN containing 0.1% CH_3_COOH, 1:1, *v*/*v*): *m*/*z* = 672, 674, and 676 [M+Na]^+^. Anal. calcd for C_23_H_17_Br_2_N_5_O_4_S_2_ (651.35 g/mol): C, 42.41; H, 2.63; N, 10.75; S, 9.85. Found: C, 42.36; H, 2.83; N, 10.81; S, 9.77%.

2,6-dichloro-*N*-(2-(1-((2,6-dichlorophenyl)sulfonyl)-4,5-dihydro-1*H*-imidazol-2-yl)phthalazin-1(2*H*)-ylidene)benzenesulfonamide (**6k**)

Starting from 0.491 g of 2,6-dichlorobenzene-1-sulfonyl chloride, yield 0.09 g (14%), eluent: CH_2_Cl_2_:CH_3_COOC_2_H_5_, 95:5, *v*:*v*, crystalized from methanol, white/beige powder, m.p. 232–234 °C; IR (KBr) *v* [cm^−1^]: 3133, 3080, 2955, 2922, 2873, 1665, 1610, 1541, 1426, 1375, 1309, 1195, 1152, 1130, 931, 828, 783, 620, 603, 571; ^1^H-NMR (DMSO-*d*_6_, 400 MHz) *δ* [ppm]: 3.78 (t, 2H, CH2), 4.01 (t, 2H, CH_2_), 7.53–7.65 (m, 6H, 6xCH-Ar), 8.13–8.16 (m, 2H, 2xCH-Ar), 8.19–8.21 (m, 1H, CH-Ar), 8.68 (s, 1H, CH-Ar), 8.75 (d, *J* = 8.1 Hz, 1H, CH-Ar); ^13^C-NMR (DMSO-*d*_6_, 100 MHz) *δ* [ppm]: 50.0, 51.4, 124.1, 128.8, 129.0, 130.4, 131.8 (two overlapping signals), 132.6 (two overlapping signals), 133.1, 133.8, 134.0 (two overlapping signals), 134.2, 134.7 (two overlapping signals), 135.2, 136.9, 138.3, 143.2, 148.1, 152.2; MS (ESI, CH_3_OH:CH_3_CN containing 0.1% CH_3_COOH, 1:1, *v*/*v*): *m*/*z* = 652, 654, 656, and 658 [M+Na]^+^. Anal. calcd for C_23_H_15_Cl_4_N_5_O_4_S_2_ (631.34 g/mol): C, 43.76; H, 2.39; N, 11.09; S, 10.16. Found: C, 43.81; H, 2.45; N, 11.21; S, 10.02%.

3,4-dichloro-*N*-(2-(1-((3,4-dichlorophenyl)sulfonyl)-4,5-dihydro-1*H*-imidazol-2-yl)phthalazin-1(2*H*)-ylidene)benzenesulfonamide (**6l**)

Starting from 0.491 g of 3,4-dichlorobenzene-1-sulfonyl chloride, yield 0.1 g (16%), eluent: CH_2_Cl_2_:CH_3_COOC_2_H_5_, 95:5, *v*/*v* and then CHCl_3_:CH_3_COOC_2_H_5_:CH_3_OH, 80:20:2, *v*/*v*/*v*, white powder, m.p. 243–246 °C; IR (KBr) *v* [cm^−1^]: 3092, 3066, 2961, 2925, 2851, 1662, 1531, 1456, 1392, 1376, 1311, 1183, 1151, 1140, 1092, 835, 645, 595; ^1^H-NMR (DMSO-*d*_6_, 400 MHz) *δ* [ppm]: 3.97 (br.s, 4H, CH_2_-CH_2_), 7.73–7.80 (m, 3H, 3xCH-Ar), 7.85 (d, *J* = 2.2 Hz, 1H, CH-Ar), 7.89 (d, *J* = 8.5 Hz, 1H, CH-Ar), 7.94 (d, *J* = 2.2 Hz, 1H, CH-Ar), 8.22–8.30 (m, 3H, 3xCH-Ar), 9.03 (s, 1H, CH-Ar), 9.09–9.11 (m, 1H, CH-Ar); MS (ESI, CH_3_OH:CH_3_CN containing 0.1% CH_3_COOH, 1:1, *v*/*v*): *m*/*z* = 652, 654, 656, and 658 [M+Na]^+^ and *m*/*z* = 693, 695, 697, and 699 [M+Na+CH_3_CN]^+^. Anal. calcd for C_23_H_15_Cl_4_N_5_O_4_S_2_ (631.34 g/mol): C, 43.76; H, 2.39; N, 11.09; S, 10.16. Found: C, 43.68; H, 2.31; N, 11.32; S, 10.22%.

*N*-(2-(1-(naphthalen-2-ylsulfonyl)-4,5-dihydro-1*H*-imidazol-2-yl)phthalazin-1(2*H*)-ylidene)naphthalene-2-sulfonamide (**6m**)

Starting from 0.453 g naphthalene-2-sulfonyl chloride, yield 0.212 g (36%), white powder, m.p. 194–196 °C; IR (KBr) *v* [cm^−1^]: 3126, 3056, 2925, 2876, 2851, 1658, 1612, 1549, 1504, 1364, 1288, 1166, 1146, 1124, 1107, 1073, 930, 823, 698, 664; ^1^H-NMR (DMSO-*d*_6_, 400 MHz) *δ* [ppm]: 3.65–3.85 (m, 4H, CH_2_-CH_2_), 7.65–7.73 (m, 4H, 4xCH-Ar), 7.81–7.87 (m, 2H, 2xCH-Ar), 7.97–8.00 (m, 2H, 2xCH-Ar), 8.03–8.07 (m, 3H, 3xCH-Ar), 8.15 (d, *J* = 8.1 Hz, 1H, CH-Ar), 8.25–8.29 (m, 3H, 3xCH-Ar), 8.44 (m, 1H, CH-Ar), 8.49–8.50 (m, 1H, CH-Ar), 9.02 (s, 1H, CH-Ar), 9.26–9.28 (m, 1H, CH-Ar); ^13^C-NMR (DMSO-*d*_6_, 100 MHz) *δ* [ppm]: 48.4, 51.1, 122.4 (two overlapping signals), 124.0, 125.8, 127.9, 128.2, 128.3, 128.3, 128.9, 129.1, 129.3, 129.4, 129.5, 130.0 (two overlapping signals), 130.1, 130.3, 130.7, 131.9, 131.9, 134.0, 134.1, 134.3, 135.2, 136.8, 141.4, 143.4, 149.2, 152.5; MS (ESI, CH_3_OH:CH_3_CN containing 0.1% CH_3_COOH, 1:1, *v*/*v*): *m*/*z* = 616 [M+Na]^+^. Anal. calcd for C_31_H_23_N_5_O_4_S_2_ (593.68 g/mol): C, 62.72; H, 3.90; N, 11.80; S, 10.80. Found: C, 62.69; H, 3.92; N, 11.74; S, 10.58%.

*N*-(2-(1-([1,1′-biphenyl]-4-ylsulfonyl)-4,5-dihydro-1*H*-imidazol-2-yl)phthalazin-1(2*H*)-ylidene)-[1,1′-biphenyl]-4-sulfonamide (**6n**)

Starting from 0.505 g [1,1′-biphenyl]-4-sulfonyl chloride, yield 0.12 g (19%), white powder, m.p. 218–222 °C; IR (KBr) *v* [cm^−1^]: 3123, 3058, 3031, 2921, 2930, 1662, 1612, 1537, 1481, 1368, 1293, 1251, 1168, 1144, 1087, 993, 933, 828, 763, 696, 676, 595; ^1^H-NMR (DMSO-*d_6_*, 400 MHz) *δ* [ppm]: 3.90–3.95 (m, 4H, CH_2_-CH_2_), 7.43–7.51 (m, 6H, 6xCH-Ar), 7.65–7.68 (m, 4H, 4xCH-Ar), 7.78–7.83 (m, 4H, 4xCH-Ar), 7.86–7.91 (m, 4H, 4xCH-Ar), 8.25–8.28 (m, 3H, 3xCH-Ar), 9.00 (s, 1H, CH-Ar), 9.25 (dd, *J*_1_ = 1.4 Hz, *J*_2_ = 7.5 Hz, 1H, CH-Ar); ^13^C-NMR (DMSO-*d*_6_, 100 MHz) *δ* [ppm]: 48.5, 51.2, 123.9, 126.4 (two overlapping signals), 127.4 (two overlapping signals), 127.5 (two overlapping signals), 127.6 (two overlapping signals), 128.1 (two overlapping signals), 128.7 (two overlapping signals), 128.8, 129.3, 129.3, 129.4, 129.5 (two overlapping signals), 129.6 (two overlapping signals), 130.7, 134.1, 135.6, 136.8, 138.4, 139.1, 143.2, 143.3, 143.8, 146.0, 149.2, 152.4; MS (ESI, CH_3_OH:CH_3_CN containing 0.1% CH_3_COOH, 1:1, *v*/*v*): *m*/*z* = 646 [M+H]^+^ and *m*/*z* = 668 [M+Na]^+^. Anal. calcd for C_35_H_27_N_5_O_4_S_2_ (645.75 g/mol): C, 65.10; H, 4.21; N, 10.85; S, 9.93. Found: C, 65.18; H, 4.14; N, 10.69; S, 9.74%.

4-phenoxy-*N*-(2-(1-((4-phenoxyphenyl)sulfonyl)-4,5-dihydro-1*H*-imidazol-2-yl)phthalazin-1(2*H*)-ylidene)benzenesulfonamide (**6o**)

Starting from 0.537 g 4-phenoxybenzene-1-sulfonyl chloride, yield 0.06 g (9%), white powder, m.p. 217–218 °C; IR (KBr) *v* [cm^−1^]: 3115; 3061; 2936; 2884; 1656; 1611; 1582; 1547; 1487; 1374; 1280; 1232; 1166; 1140; 1089; 933; 873; 829; 695; 678; 599; 577; 560; ^1^H-NMR (DMSO-*d*_6_, 400 MHz) *δ* [ppm]: 3.88 (m, 4H, CH_2_-CH_2_), 7.02–7.04 (m, 2H, 2xCH-Ar), 7.10–7.16 (m, 6H, 6xCH-Ar), 7.23–7.27 (m, 1H, CH-Ar), 7.28–7.32 (m, 1H, CH-Ar), 7.45–7.49 (m, 4H, 4xCH-Ar), 7.80–7.83 (m, 4H, 4xCH-Ar), 8.22–8.25 (m, 3H, 3xCH-Ar), 8.95 (s, 1H, CH-Ar), 9.16–9.19 (m, 1H, CH-Ar); ^13^C-NMR (DMSO-*d*_6_, 100 MHz) *δ* [ppm]: 48.3, 51.1, 117.8 (two overlapping signals), 118.2, 119.9 (two overlapping signals), 120.9 (two overlapping signals), 123.9, 125.0, 125.9, 128.2 (two overlapping signals), 129.2, 129.3, 130.5, 130.6, 130.8, 130.8 (two overlapping signals), 130.8 (two overlapping signals), 131.0 (two overlapping signals), 134.0, 136.8, 139.0, 143.2, 149.2, 152.3, 155.0, 155.9, 160.0, 162.4; MS (ESI, CH_3_OH:CH_3_CN containing 0.1% CH_3_COOH, 1:1, *v*/*v*): *m*/*z* = 678 [M+H]^+^, *m*/*z* = 700 [M+Na]^+^ and *m*/*z* = 741 [M+Na+CH_3_CN]^+^. Anal. calcd for C_35_H_27_N_5_O_6_S_2_ (677.75 g/mol): C, 62.03; H, 4.02; N, 10.33; S, 9.46. Found: C, 61.89; H, 3.95; N, 10.47; S, 9.53%.

2-(((1-(methylsulfonyl)imidazolidin-2-ylidene)hydrazono)methyl)benzonitrile (**7a**)

Starting from 0.229 g of methanesulfonyl chloride (0.155 mL, d = 1.48 g/mL), yield 0.1 g (34%), eluent: CH_2_Cl_2_: CH_3_COOC_2_H_5_, 95:5, *v*/*v*, white powder, m.p. 245–248 °C; IR (KBr) *v* [cm^−1^]: 3351 (NH), 3022, 2990, 2965, 2918, 2233 (CN), 1629, 1601, 1575, 1488, 1425, 1350, 1339, 1279, 1160, 1169, 1077, 1062, 1005, 783, 554; ^1^H-NMR (DMSO-*d*_6_, 400 MHz) *δ* [ppm]: 3.43 (s, 3H, CH_3_), 3.50–3.54 (m, 2H, CH_2_), 3.92–3.96 (m, 2H, CH_2_), 7.55 (td, 1H, CH-Ar), 7.74–7.76 (m, 2H, CH-Ar+NH), 7.86–7.88 (m, 1H, CH-Ar), 8.25–8.27 (m, 1H, CH-Ar), 8.42 (s, 1H, CH=N); ^13^C-NMR (DMSO-*d_6_*, 100 MHz) *δ* [ppm]: 39.7, 40.5, 46.6, 110.3, 118.2, 127.3, 130.0, 133.6, 133.8, 138.4, 147.0, 159.1; MS (ESI, MeOH:MeCN + 0.1% AcOH, 1:1, *v*/*v*): *m*/*z* = 292 [M+H]^+^, *m*/*z* = 314 [M+Na]^+^ and *m*/*z* = 355 [M+Na+CH_3_CN]^+^. Anal. calcd for C_12_H_13_N_5_O_2_S (291.33 g/mol): C, 49.47; H, 4.50; N, 24.04; S, 11.01. Found: C, 49.38; H, 4.59; N, 24.14; S, 11.12%.

2-(((1-(phenylsulfonyl)imidazolidin-2-ylidene)hydrazono)methyl)benzonitrile (**7b**)

Starting from 0.353 g of benzenesulfonyl chloride (0.255 mL, d = 1.384 g/mL), yield 0.038 g (11%), white powder, m.p. 167–173 °C; IR (KBr) *v* [cm^−1^]: 3446, 3395 (NH), 3065, 2984, 2958, 2896, 2851, 2226 (CN), 1634, 1602, 1557, 1480, 1447, 1420, 1356, 1276, 1171, 1092, 1064, 1000, 757, 727, 685, 606, 574; ^1^H-NMR (DMSO-*d*_6_, 400 MHz) *δ* [ppm]: 3.46–3.50 (m, 2H, CH_2_), 4.00–4.04 (m, 2H, CH_2_), 7.51–7.54 (m, 1H, CH-Ar), 7.58 (s, 1H, NH), 7.62–7.66 (m, 2H, 2xCH-Ar), 7.69–7.74 (m, 2H, 2xCH-Ar), 7.85–7.87 (m, 1H, CH-Ar), 8.07–8.09 (m, 2H, 2xCH-Ar), 8.17–8.19 (m, 1H, CH-Ar), 8.38 (s, 1H, CH=N); ^13^C-NMR (DMSO-*d*_6_, 100 MHz) *δ* [ppm]: 40.1, 47.5, 110.3, 118.2, 127.4, 128.7 (two overlapping signals), 129.4 (two overlapping signals), 130.0, 133.6, 133.8, 134.5, 137.7, 138.2, 147.3, 158.1; MS (ESI, CH_3_OH:CH_3_CN containing 0.1% CH_3_COOH, 1:1, *v*/*v*): *m*/*z* = 354 [M+H]^+^, *m*/*z* = 376 [M+Na]^+^ and *m*/*z* = 417 [M+CH_3_CN]^+^. Anal. calcd for C_17_H_15_N_5_O_2_S (353.40 g/mol): C, 57.78; H, 4.28; N, 19.82; S, 9.07. Found: C, 57.52; H, 4.12; N, 19.97; S, 8.89%.

2-(((1-tosylimidazolidin-2-ylidene)hydrazono)methyl)benzonitrile (**7c**)

Starting from 0.3813 g of 4-methylbenzene-1-sulfonyl chloride, yield 0.07 g (19%), white powder, m.p. 209–214 °C; IR (KBr) *v* [cm^−1^]: 3425, 3396 (NH), 3101, 3063, 3031, 2961, 2894, 2225 (CN), 1633, 1600, 1572, 1480, 1414, 1354, 1273, 1169, 1059, 1000, 667, 592; ^1^H-NMR (DMSO-*d*_6_, 400 MHz) *δ* [ppm]: 2.39 (s, 3H, CH_3_), 3.45–3.48 (m, 2H, CH_2_), 3.97–4.00 (m, 2H, CH_2_), 7.43 (d, *J* = 8.3 Hz, 2H, 2xCH-Ar), 7.53–7.56 (m, 2H, CH-Ar+NH), 7.71–7.73 (m, 1H, CH-Ar), 7.86 (dd, *J*_1_ = 1.3 Hz, *J*_2_ = 7.8 Hz, 1H, CH-Ar), 7.95–7.97 (m, 2H, 2xCH-Ar), 8.18 (dd, *J*_1_ = 1.2 Hz, *J*_2_ = 8.1 Hz, 1H, CH-Ar), 8.39 (s, 1H, CH=N); ^13^C-NMR (DMSO-*d_6_*, 100 MHz) *δ* [ppm]: 21.5, 40.6, 47.5, 110.2, 118.3, 127.4, 128.8 (two overlapping signals), 129.9 (two overlapping signals), 130.0, 133.6, 133.8, 134.7, 138.2, 145.1, 147.2, 158.2; MS (ESI, CH_3_OH:CH_3_CN containing 0.1% CH_3_COOH, 1:1, *v*/*v*): *m*/*z* = 368 [M+H]^+^, *m*/*z* = 390 [M+Na]^+^ and *m*/*z* = 422 [M+Na+CH_3_OH]^+^. Anal. calcd for C_18_H_17_N_5_O_2_S (367.42 g/mol): C, 58.84; H, 4.66; N, 19.06; S, 8.73. Found: C, 58.76; H, 4.72; N, 19.32; S, 8.68%.

2-(((1-((3-methoxyphenyl)sulfonyl)imidazolidin-2-ylidene)hydrazono)methyl)benzonitrile (**7d**)

Starting from 0.381 g of 3-methoxybenzene-1-sulfonyl chloride, yield 0.06 g (16%), eluent: CH_2_Cl_2_:CH_3_COOC_2_H_5_, 95:5, v/v, crystallized from methanol, white powder, m.p. 199–203 °C; IR (KBr) *v* [cm^−1^]: 3393 (NH), 3106, 3006, 2966, 2923, 2832, 2220 (CN), 1635, 1601, 1576, 1478, 1419, 1354, 1279, 1255, 1167, 1096, 1064, 1002, 761, 623, 567, 531; ^1^H-NMR (DMSO-*d_6_*, 400 MHz) *δ* [ppm]: 3.48 (t, 2H, CH_2_), 3.84 (s, 3H, OCH_3_), 3.99 (t, 2H, CH_2_), 7.29–7.31 (m, 1H, CH-Ar), 7.54–7.57 (m, 2H, 2xCH-Ar), 7.61–7.63 (m, 1H, CH-Ar), 7.67 (m, 2H, CH-Ar+NH), 7.72 (td, 1H, CH-Ar), 7.87 (dd, *J*_1_ = 1.3 Hz, *J*_2_ = 7.9 Hz, 1H, CH-Ar), 8.21 (d, *J* = 7.9 Hz, 1H, CH-Ar), 8.43 (s, 1H, CH=N); ^13^C-NMR (DMSO-*d*_6_, 100 MHz) *δ* [ppm]: 40.7, 47.5, 56.1, 110.4, 113.5, 118.1, 120.6, 121.0, 127.2, 130.1, 130.6, 133.6, 133.7, 138.2, 138.6, 147.1, 158.2, 159.5; MS (ESI, CH_3_OH:CH_3_CN containing 0.1% CH_3_COOH, 1:1, *v*/*v*): *m*/*z* = 384 [M+H]^+^ and *m*/*z* = 406 [M+Na]^+^. Anal. calcd for C_18_H_17_N_5_O_3_S (383.42 g/mol): C, 56.38; H, 4.47; N, 18.27; S, 8.36. Found: C, 56.45; H, 4.56; N, 18.15; S, 8.32%.

2-(((1-((4-(tert-butyl)phenyl)sulfonyl)imidazolidin-2-ylidene)hydrazono)methyl)benzonitrile (**7e**)

Starting from 0.466 g of 4-(*tert*-butyl)benzene-1-sulfonyl chloride, yield 0.18 g (44%), crystallized from methanol, yellowish crystalline powder, m.p. 220–223 °C; IR (KBr) *v* [cm^−1^]: 3448, 3063, 2968, 2905, 2220, 1641, 1594, 1575, 1478, 1416, 1351, 1280, 1172, 1115, 1087, 1061, 1000, 769, 634, 597; ^1^H-NMR (DMSO-*d*_6_, 400 MHz) *δ* [ppm]: 1.29 (s, 9H, C(CH_3_)_3_), 3.45–3.49 (m, 2H, CH_2_), 3.96–4.00 (m, 2H, CH_2_), 7.54 (td, 1H, CH-Ar), 7.59 (s, 1H, NH), 7.64–7.66 (m, 2H, 2xCH-Ar), 7.70–7.72 (m, 1H, CH-Ar), 7.85–7.88 (m, 1H, CH-Ar), 7.99–8.02 (m, 2H, 2xCH-Ar), 8.18–8.20 (m, 1H, CH-Ar), 8.41 (s, 1H, CH=N); ^13^C-NMR (DMSO-*d*_6_, 100 MHz) *δ* [ppm]: 31.2 (three overlapping signals), 35.5, 40.7, 47.5, 110.3, 118.2, 126.4 (two overlapping signals), 127.3, 128.7 (two overlapping signals), 130.1, 133.6, 133.8, 134.8, 138.2, 147.3, 157.6, 158.2; MS (ESI, CH_3_OH:CH_3_CN containing 0.1% CH_3_COOH, 1:1, *v*/*v*): *m*/*z* = 410 [M+H]^+^, *m*/*z* = 432 [M+Na]^+^ and *m*/*z* = 473 [M+Na+CH_3_CN]^+^. Anal. calcd for C_21_H_23_N_5_O_2_S (409.50 g/mol): C, 61.59; H, 5.66; N, 17.10; S, 7.83. Found: C, 61.69; H, 5.68; N, 17.28; S, 7.65%.

2-(((1-((2-chlorophenyl)sulfonyl)imidazolidin-2-ylidene)hydrazono)methyl)benzonitrile (**7f**)

Starting from 0.422 g of 2-chlorobenzene-1-sulfonyl chloride, yield 0.05 g (13%), eluent: CHCl_3_:CH_3_COOC_2_H_5_:CH_3_COCH_3_, 8:1:1, *v*/*v*/*v*, pale yellow color crystals, m.p. 210–214 °C; IR (KBr) *v* [cm^−1^]: 3376 (NH), 3102, 3065, 2987, 2961, 2918, 2851, 2219 (CN), 1648, 1600, 1578, 1480, 1427, 1350, 1280, 1173, 1115, 1086, 1066, 1002, 753, 605; ^1^H-NMR (DMSO-*d*_6_, 400 MHz) *δ* [ppm]: 3.60 (t, 2H, CH_2_), 4.25 (t, 2H, CH_2_), 7.51 (td, 1H, CH-Ar), 7.61–7.72 (m, 5H, 4xCH-Ar+NH), 7.82 (dd, *J*_1_ = 1.3 Hz, *J*_2_ = 7.8 Hz, 1H, CH-Ar), 8.01 (s, 1H, CH=N), 8.14 (d, *J* = 8.6 Hz, 1H, CH-Ar), 8.24 (dd, *J*_1_ = 1.3 Hz, *J*_2_ = 7.8 Hz, 1H, CH-Ar); ^13^C-NMR (DMSO-*d*_6_, 100 MHz) *δ* [ppm]: 40.4, 46.8, 110.3, 118.0, 127.1, 127.8, 130.0, 131.2, 132.1, 133.5, 133.7, 133.7, 135.6, 137.0, 138.0, 147.1, 157.4; MS (ESI, CH_3_OH:CH_3_CN containing 0.1% CH_3_COOH, 1:1, *v*/*v*): *m*/*z* = 388, 390 [M+H]^+^, *m*/*z* = 410, 412 [M+Na]^+^ and *m*/*z* = 451, 453 [M+Na+CH_3_CN]^+^. Anal. calcd for C_17_H_14_ClN_5_O_2_S (387.84 g/mol): C, 52.65; H, 3.64; N, 18.06; S, 8.27. Found: C, 52.87; H, 3.71; N, 17.95; S, 8.21%.

2-(((1-((4-chlorophenyl)sulfonyl)imidazolidin-2-ylidene)hydrazono)methyl)benzonitrile (**7g**)

Starting from 0.422 g of 4-chlorobenzene-1-sulfonyl chloride, yield 0.083 g (22%), white/beige powder, m.p. 222–226 °C; IR (KBr) *v* [cm^−1^]: 3421, 3385, 3084, 2965, 2898, 2857, 2225, 1636, 1601, 1576, 1475, 1412, 1359, 1273, 1179, 1170, 1093, 1058, 997, 756, 627; ^1^H-NMR (DMSO-*d*_6_, 400 MHz) *δ* [ppm]: 3.49 (t, 2H, CH_2_), 4.02 (t, 2H, CH_2_), 7.54 (t, 1H, CH-Ar), 7.63 (s, 1H, NH), 7.70–7.74 (m, 3H, 3xCH-Ar), 7.87 (d, *J* = 7.8 Hz, 1H, CH-Ar), 8.09 (d, *J* = 8.4 Hz, 2H, 2xCH-Ar), 8.18 (d, *J* = 8.0 Hz, 1H, CH-Ar), 8.38 (s, 1H, CH=N); ^13^C-NMR (DMSO-*d*_6_, 100 MHz) *δ* [ppm]: 40.1, 47.5, 110.3, 118.2, 127.4, 129.6 (two overlapping signals), 130.1, 130.7 (two overlapping signals), 133.6, 133.8, 136.5, 138.2, 139.5, 147.5, 158.0; MS (ESI, CH_3_OH:CH_3_CN containing 0.1% CH_3_COOH, 1:1, *v*/*v*): *m*/*z* = 388, 390 [M+H]^+^, *m*/*z* = 410, 412 [M+Na]^+^ and *m*/*z* = 451, 453 [M+Na+CH_3_CN]^+^. Anal. calcd for C_17_H_14_ClN_5_O_2_S (387.84 g/mol): C, 52.65; H, 3.64; N, 18.06; S, 8.27. Found: C, 52.48; H, 3.88; N, 18.17; S, 8.16%.

2-(((1-((4-bromophenyl)sulfonyl)imidazolidin-2-ylidene)hydrazono)methyl)benzonitrile (**7h**)

Starting from 0.511 g of 4-bromobenzene-1-sulfonyl chloride, yield 0.012 g (3%), eluent: CH_2_Cl_2_:CH_3_COOC_2_H_5_, 95:5, *v*/*v*, white powder, m.p. 217–220 °C; IR (KBr) *v* [cm^−1^]: 3386 (NH), 3092, 3022, 2915, 2223 (CN), 1638, 1592, 1575, 1481, 1416, 1390, 1353, 1277, 1171, 1090, 1066, 1009, 1000, 744, 617; ^1^H-NMR (DMSO-*d*_6_, 400 MHz) *δ* [ppm]: 3.47–3.51 (m, 2H, CH_2_), 4.00–4.04 (m, 2H, CH_2_), 7.54 (td, 1H, CH-Ar), 7.62 (s, 1H, NH), 7.72 (td, 1H, CH-Ar), 7.85–7.87 (m, 3H, 3xCH-Ar), 7.99–8.02 (m, 2H, 2xCH-Ar), 8.17–8.19 (m, 1H, CH-Ar), 8.38 (s, 1H, CH=N); ^13^C-NMR (DMSO-*d*_6_, 100 MHz) *δ* [ppm]: 40.1, 47.5, 110.3, 118.2, 127.4, 128.6, 130.1, 130.7 (two overlapping signals), 132.6 (two overlapping signals), 133.6, 133.8, 136.9, 138.2, 147.5, 157.9; MS (ESI, CH_3_OH:CH_3_CN containing 0.1% CH_3_COOH, 1:1, *v*/*v*): *m*/*z* = 434, 436 [M+H]^+^ and *m*/*z* = 456, 458 [M+Na]^+^. Anal. calcd for C_17_H_14_BrN_5_O_2_S (432.29 g/mol): C, 47.23; H, 3.26; N, 16.20; S, 7.42. Found: C, 47.36; H, 3.12; N, 16.16; S, 7.56%.

2-(((1-((2,6-dichlorophenyl)sulfonyl)imidazolidin-2-ylidene)hydrazono)methyl)benzonitrile (**7i**)

Starting from 0.491 g of 2,6-dichlorobenzene-1-sulfonyl chloride, yield 0.025 g (6%), eluent: CH_2_Cl_2_:CH_3_COOC_2_H_5_, 95:5, *v*/*v*, white powder, m.p. 197–200 °C; IR (KBr) *v* [cm^−1^]: 3448 (NH), 3066, 2984, 2961, 2919, 2222 (CN), 1638, 1600, 1572, 1478, 1430, 1417, 1359, 1279, 1185, 1060, 1001, 786, 611, 591; ^1^H-NMR (DMSO-*d*_6_, 400 MHz) *δ* [ppm]: 3.62 (t, 2H, CH_2_), 4.23 (t, 2H, CH_2_), 7.52 (t, 1H, CH-Ar), 7.59–7.65 (m, 1H, CH-Ar), 7.67–7.71 (m, 3H, 3xCH-Ar), 7.75 (br.s, 1H, NH), 7.82 (d, *J* = 7.6 Hz, 1H, CH-Ar), 8.09 (s, 1H, CH=N), 8.15 (d, *J* = 8.0 Hz, 1H, CH-Ar); ^13^C-NMR (DMSO-*d*_6_, 100 MHz) *δ* [ppm]: 41.0, 46.1, 110.5, 117.8, 126.9, 130.1, 132.2 (two overlapping signals), 132.4, 133.5, 133.6, 134.7, 134.8, 135.7, 138.0, 147.4, 157.5; MS (ESI, CH_3_OH:CH_3_CN containing 0.1% CH_3_COOH, 1:1, *v*/*v*): *m*/*z* = 422, 424, and 426 [M+H]^+^, *m*/*z* = 444, 446, and 448 [M+Na]^+^. Anal. calcd for C_17_H_13_Cl_2_N_5_O_2_S (422.29 g/mol): C, 48.35; H, 3.10; N, 16.58; S, 7.59. Found: C, 48.28; H, 3.17; N, 16.29; S, 7.34%.

2-(((1-((3,4-dichlorophenyl)sulfonyl)imidazolidin-2-ylidene)hydrazono)methyl)benzonitrile (**7j**)

Starting from 0.491 g of 2,6-dichlorobenzene-1-sulfonyl chloride, yield 0.04 g (9%), eluent: CH_2_Cl_2_ and CH_2_Cl_2_:CH_3_COOC_2_H_5_, 95:5, *v*/*v*, white powder, m.p. 200–201 °C; IR (KBr) *v* [cm^−1^]: 3389 (NH), 3096, 2914, 2225 (CN), 1640, 1601, 1578, 1481, 1416, 1372, 1277, 1175, 1100, 1061, 631; ^1^H-NMR (DMSO-*d*_6_, 400 MHz) *δ* [ppm]: 3.48–3.52 (m, 2H, CH_2_), 4.04–4.07 (m, 2H, CH_2_), 7.55 (td, 1H, CH-Ar), 7.72–7.74 (m, 2H, CH-Ar+NH), 7.87 (dd, *J*_1_ = 1.3 Hz, *J*_2_ = 7.8 Hz, 1H, CH-Ar), 7.92 (d, *J* = 8.5 Hz, 1H, CH-Ar), 8.03 (dd, *J*_1_ = 2.2 Hz, *J*_2_ = 8.5 Hz, 1H, CH-Ar), 8.19–8.21 (m, 1H, CH-Ar), 8.35 (d, *J* = 2.2 Hz, 1H, CH-Ar), 8.41 (s, 1H, CH=N); ^13^C-NMR (DMSO-*d*_6_, 100 MHz) 40.3, 47.4, 110.5, 118.0, 127.2, 128.7, 130.2, 131.1, 131.8, 132.2, 133.6, 133.8, 137.6, 137.7, 138.1, 147.5, 157.7; MS (ESI, CH_3_OH:CH_3_CN containing 0.1% CH_3_COOH, 1:1, *v*/*v*): *m*/*z* = 422, 424, and 426 [M+H]^+^, *m*/*z* = 444, 446, and 448 [M+Na]^+^ and *m*/*z* = 485 [M+Na+MeCN]^+^. Anal. calcd for: C_17_H_13_Cl_2_N_5_O_2_S (422.29 g/mol): C, 48.35; H, 3.10; N, 16.58; S, 7.59. Found: C, 48.28; H, 3.19; N, 16.69; S, 7.35%.

2-(((1-((4-nitrophenyl)sulfonyl)imidazolidin-2-ylidene)hydrazono)methyl)benzonitrile (**7k**)

Starting from 0.443 g of 4-nitrobenzene-1-sulfonyl chloride, yield 0.05 g (13%), yellowish crystalline powder, m.p. 227–233 °C; IR (KBr) *v* [cm^−1^]: 3405 (NH), 3116, 3099, 3072, 3009, 2903, 2870, 2219 (CN), 1640, 1594, 1531, 1402, 1363, 1348, 1274, 1182, 1092, 1058, 993, 855, 740, 618; ^1^H-NMR (DMSO-*d_6_*, 400 MHz) *δ* [ppm]: 3.50–3.53 (m, 2H, CH_2_), 4.07–4.10 (m, 2H, CH_2_), 7.54 (td, 1H, CH-Ar), 7.69 (br.s, 1H, NH), 7.72 (td, 1H, CH-Ar), 7.87 (dd, *J*_1_ = 1.2 Hz, *J*_2_ = 7.8 Hz, 1H, CH-Ar), 8.16–8.18 (m, 1H, CH-Ar), 8.34–8.36 (m, 2H, 2xCH-Ar), 8.39 (s, 1H, CH=N), 8.42–8.45 (m, 2H, 2xCH-Ar); ^13^C-NMR (DMSO-*d*_6_, 100 MHz) *δ* [ppm]: 40.0, 47.5, 110.4, 118.2, 124.7 (two overlapping signals), 127.4, 130.2, 130.4 (two overlapping signals), 133.6, 133.8, 138.1, 142.9, 147.8, 150.9, 157.6; MS (ESI, CH_3_OH:CH_3_CN containing 0.1% CH_3_COOH, 1:1, *v*/*v*): *m*/*z* = 399 [M+H]^+^. Anal. calcd for C_17_H_14_N_6_O_4_S (398.40 g/mol): C, 51.25; H, 3.54; N, 21.09; S, 8.05. Found: C, 51.31; H, 4.08; N, 21.23; S, 7.92%.

2-(((1-(naphthalen-2-ylsulfonyl)imidazolidin-2-ylidene)hydrazono)methyl)benzonitrile (**7l**)

Starting from 0.453 g naphthalene-2-sulfonyl chloride, yield 0.087 g (22%), crystallized from methanol, white powder, m.p. 206–209 °C; IR (KBr) *v* [cm^−1^]: 3413 (NH), 3069, 3058, 2892, 2226 (CN), 1634, 1600, 1572, 1478, 1412, 1352, 1272, 1170, 1074, 1060, 999, 769, 663; 638, 581, 541; ^1^H-NMR (DMSO-*d_6_*, 400 MHz) *δ* [ppm]: 3.47–3.51 (m, 2H, CH_2_), 4.08–4.11 (m, 2H, CH_2_), 7.52 (td, 1H, CH-Ar), 7.59 (s, 1H, NH); 7,66–7,74 (m, 3H, 3xCH-Ar), 7.85 (dd, *J*_1_ = 1.3 Hz, *J*_2_ = 7.8 Hz, 1H, CH-Ar), 8.04–8.07 (m, 1H, CH-Ar), 8.10 (d, *J* = 1.9 Hz, 1H, CH-Ar), 8.13–8.16 (m, 2H, 2xCH-Ar), 8.22 (dd, *J*_1_ = 1.0 Hz, *J*_2_ = 7.8 Hz, 1H, CH-Ar), 8.42 (s, 1H, CH=N), 8.78 (s, 1H, CH-Ar); ^13^C-NMR (DMSO-*d*_6_, 100 MHz) *δ* [ppm]: 40.1, 47.6, 110.3, 118.2, 123.7, 127.3, 128.2, 128.3, 129.3, 129.9, 129.9, 130.1, 130.7, 131.9, 133.5, 133.7, 134.6, 135.3, 138.2, 147.2, 158.1; MS (ESI, CH_3_OH:CH_3_CN containing 0.1% CH_3_COOH, 1:1, *v*/*v*): *m*/*z* = 404 [M+H]^+^, *m*/*z* = 426 [M+Na]^+^ and *m*/*z* = 467 [M+Na+CH_3_CN]^+^. Anal. calcd for C_21_H_17_N_5_O_2_S (403.46 g/mol): C, 62.52; H, 4.25; N, 17.36; S, 7.95. Found: C, 62.42; H, 4.37; N, 17.43; S, 7.87%.

2-(((1-([1,1′-biphenyl]-4-ylsulfonyl)imidazolidin-2-ylidene)hydrazono)methyl)benzonitrile (**7m**)

Starting from 0.505 g [1,1′-biphenyl]-4-sulfonyl chloride, yield 0.015 g (3.5%), white powder, m.p. 212–216 °C; IR (KBr) *v* [cm^−1^]: 3399 (NH), 3082, 3015, 2961, 2923, 2851, 2220 (CN), 1634, 1571, 1554, 1477, 1424, 1361, 1278, 1170, 1070, 1006, 768, 760, 676, 610; ^1^H-NMR (DMSO-*d*_6_, 400 MHz) *δ* [ppm]: 3.50 (t, 2H, CH_2_), 4.05 (t, 2H, CH_2_), 7.44–7.53 (m, 4H, 4xCH-Ar), 7.62 (s, 1H, NH), 7,71–7,76 (m, 3H, 3xCH-Ar), 7.86 (dd, *J*_1_ = 1.2 Hz, *J*_2_ = 7.8 Hz, 1H, CH-Ar), 7.92 (d, *J* = 8.5 Hz, 2H, 2xCH-Ar), 8.15 (d, *J* = 8.5 Hz, 2H, 2xCH-Ar), 8.19 (d, *J* = 8.0 Hz, 1H, CH-Ar), 8.43 (s, 1H, CH=N); ^13^C-NMR (DMSO-*d*_6_, 100 MHz) *δ* [ppm]: 40.1, 47.5, 110.3, 118.3, 127.4, 127.6 (two overlapping signals), 127.7 (two overlapping signals), 129.2, 129.5 (two overlapping signals), 129.6 (two overlapping signals), 130.1, 133.6, 133.8, 136.4, 138.2, 138.7, 145.9, 147.3, 158.1; MS (ESI, CH_3_OH:CH_3_CN containing 0.1% CH_3_COOH, 1:1, *v*/*v*): *m*/*z* = 430 [M+H]^+^, *m*/*z* = 452 [M+Na]^+^ and *m*/*z* = 493 [M+Na+CH_3_CN]^+^. Anal. calcd for C_23_H_19_N_5_O_2_S (429.49 g/mol): C, 64.32; H, 4.46; N, 16.31; S, 7.47. Found C, 64.28; H, 4.39; N, 16.47; S, 7.35%.

### 3.2. Antioxidant Studies

#### 3.2.1. Materials

Ascorbic acid, DPPH (2,2-diphenyl-1-picrylhydrazyl), ABTS (2,2′-azino-bis(3-ethylbenzothiazoline-6-sulfonic acid) diammonium salt, potassium persulfate, TPTZ (2,4,6-Tris(2-pyridyl)-s-triazine), β-carotene, linoleic acid, DMSO (dimethyl sulfoxide) were sourced from the Sigma Chemical Co. (St. Louis, MO, USA). HCl, FeCl_3_ × 6 H_2_O, acetic acid, chloroform, Tween-40, and HPLC-grade methanol were sourced from Avantor Performance Materials Poland S.A. (formerly POCH, Gliwice, Poland).

#### 3.2.2. DPPH Assay

The DPPH radical scavenging assay of tested compounds was performed with ascorbic acid as a positive control [68,69]. Briefly, 100 μL of different concentrations of the compounds, dissolved in DMSO, were mixed with 100 μL of 0.06 mM DPPH methanolic solution and incubated at room temperature in the dark for 30 min. The change in absorbance at λ = 517 nm was analyzed with the use of a 96-well microplate reader (Epoch, BioTek System, Winooski, VT, USA). The control was composed of DPPH and DMSO. DPPH inhibition was calculated according to the following equation:

DPPH Inhibition (%) = [(Acontrol − Asample)/Acontrol] × 100%

The radical scavenging activity of the samples was expressed as the IC_50_ value (the concentration of the analyzed samples that caused a 50% decrease in the non-reduced form of the DPPH radical).

#### 3.2.3. ABTS Assay

The ABTS radical scavenging assay of samples was performed with ascorbic acid as a positive control [68,69]. Briefly: 30 μL of different concentrations of the samples, dissolved in DMSO, were mixed with 170 μL of ABTS solution (2 mM ABTS diammonium salt, 3.5 mM potassium persulfate) and completed with water to a final volume of 300 μL. ABTS solution with DMSO was used as a control. After 10 min of incubation at 30 °C in the dark, the change in absorbance was observed at λ = 750 nm by a 96-well microplate reader (Epoch, BioTek System, Santa Clara, CA, USA). ABTS inhibition was calculated according to the following equation:

ABTS Inhibition (%) = [(Acontrol − Asample)/Acontrol] × 100%

The radical scavenging activity of the samples was shown as the IC_50_ value (the concentration of the analyzed samples that caused a 50% decrease in the non-reduced form of the ABTS radical).

#### 3.2.4. β-Carotene Bleaching Test

The β-Carotene bleaching test was based on the method described by Olszowy et al. [70] with some modifications. First, 1 mg of β-Carotene was dissolved in 5 mL of chloroform. Afterwards, 25 mg/mL of linoleic acid and 200 mg of Tween 40 were added. The chloroform was evaporated, and 50 mL of oxygenated water was added. The freshly created emulsion was mixed with different concentrations of tested compounds. β-Carotene bleaching was analyzed at λ = 492 nm before (t = 0 min) and after 90 min (t = 90 min) of incubation at 50 °C in a water bath (Epoch, BioTek Instruments, Santa Clara, CA, USA). Ascorbic acid was used as a standard. The control was composed of an emulsion and DMSO. The results were calculated according to the following equation:

[(AsampleT0 − AsampleT90)/(AcontrolT0 − AcontrolT90) × 100%

The radical scavenging activity of the samples was shown as the IC_50_ value (the concentration of the analyzed samples that caused a 50% decrease in the β-Carotene relative to the control).

#### 3.2.5. FRAP Test

The reducing ability of tested compounds was determined with the FRAP test, based on the reduction of Fe^3+^ to Fe^2+^ [71]. The course of the analysis was as follows. Here, 30 μL of serial dilutions of the tested compounds and standard substance (placed in a 96-well plate) were mixed with 170 μL of the freshly prepared reaction mixture (0.3 M acetate buffer: 10 mM TPTZ (2,4,6-tri(2-pyridyl)-*s*-triazine) in 40 mM HCl: 20 mM FeCl_3_ × 6H_2_O at a ratio of 10:1:1 (*v*/*v*). The plate was incubated at room temperature for 20 min; then, the absorbance was measured at λ = 593 nm. The percentage of reduced iron ions was read from the calibration curve plotted for ascorbic acid (1–1000 µg/mL). The assay was conducted in three independent analyses, with three replicates each. The IC_50_ value—the concentration of the analyzed extract or standard substance (ascorbic acid) that reduces iron ions by 50%—was calculated using GraFit v.7.0 (Erithacus Software, East Grinstead, UK).

### 3.3. ELISA Biological Screening

The in vitro inhibitors screening of AGE2-BSA (glyceraldehyde-modified AGE)-sRAGE (soluble RAGE) interaction was performed using a 96-well AGE-BSA-coated plate (Creative BioMart^®^ ELISA kit, MBL - Medical & Biological Laboratories CO., LTD. A JSR Life Science Company, Japan), according to the manufacturer’s instructions. 100 UA/mL soluble RAGE (sRAGE) was incubated with 10 or 100 μM of the tested compounds or 0.05% DMSO as a negative control on an AGE2-BSA-coated plate at room temperature for 60 min, shaking at 300 rpm on an orbital microplate shaker. After incubation, the wells were washed four times with wash buffer and horseradish peroxidase (HRP)-labeled anti-RAGE antibody was added, and the plate was further incubated at room temperature for 60 min, shaking at 300 rpm on an orbital microplate shaker. After incubation, the wells were washed four times with wash buffer and the Substrate Reagent was added to each well. After an incubation at room temperature for 5 min, the HRP-labeled antibody sRAGE-AGE complex was then detected by measuring the absorbance at 450 nm using the microplate reader Varioskan (Thermo Fischer Scientific). These experiments were repeated two times.

## 4. Conclusions

In this work, a novel hybrid compound—2-(4,5-dihydro-1*H*-imidazol-2-yl)phthalazin-1(2*H*)-imine (**5**) was synthesized in the reaction of pseudo-base—2-(4,5-dihydro-1*H*-imidazol-2-yl)-1,2-dihydrophthalazin-1-ol with an excess of (aminooxy)sulfonic acid (HOSA). Single crystal X-ray crystallographic studies revealed that phthalazin-1(2*H*)-imine derivative **5** in crystals adopts a nearly flat structure as a result of the intramolecular resonance assisted N-H···N hydrogen bond. 

It was found that the reaction of **5** with sulfonyl chloride bearing an electron-donating group yielded di-substituted sulfonamides **6** as the main products. In contrast, the presence of an electron-withdrawing substituent in the structure of sulfonyl chloride may promote the formation of benzonitriles **7** due to the phthalazine ring opening. Spectroscopic data and X-ray diffraction analysis confirmed their chemical structure. The tautomeric equilibrium was theoretically evaluated for compound **7k** at an ab initio level using the density functional (B3LYP) method. In silico calculations revealed that the tautomeric equilibrium in the hydrazonomethylbenzonitriles **7** lies quantitatively on the side of the iminoimidazolidine form. 

The antitumor evaluation showed that none of the tested compounds exhibited antiproliferative properties against the cancer cell lines used. In turn, the antioxidant properties of compounds **5**, **6a–o**, and **7a–m** were screened with four colorimetric methods: ABTS, DPPH, FRAP, and β˗carotene bleaching test. In vitro assays revealed that a moderate ABTS antiradical effect was observed for sulfonamide containing bromine substituent **6j** (IC_50_ = 52.77 µg/mL). On the other hand, hydrazonomethylbenzonitrile **7i** bearing two chlorine atoms on a phenyl ring system showed activity in a β˗carotene bleaching test (IC_50_ = 86.21 µg/mL). The lower in vitro anti-radical activity of tested compounds was confirmed by in silico calculations. 

Finally, the interactions of AGE/RAGE in the presence of sulfonamides **6a**, **6b**, **6g**, and **6m** and hydrazonomethylbenzonitriles **7a**, **7c**–**g**, and **7i**–**k** were tested by an ELISA assay. A moderate inhibitory potency toward RAGE was observed for hydrazonomethylbenzonitrile **7d** with an electron-donating methoxy group (R = 3-CH_3_O-C_6_H_4_) and hydrazonomethylbenzonitrile derivatives bearing an electron-withdrawing substituent **7f** (R = 2-Cl-C_6_H_4_) and **7k** (R = 4-NO_2_-C_6_H_4_). In summary, a preliminary biological evaluation may be useful as a starting point for the development of novel antioxidant agents and compounds interacting in an anti-AGE/RAGE manner. In this context, the lack of cytotoxicity appears to be a beneficial feature of the tested compounds as potential medical agents.

## Figures and Tables

**Table 1:** Crystal data and details of structure refinement.

Compound Reference	5	6e	6c	7k	7g
CCDC number	2361137	2361139	2361140	2361141	2361138
Chemical formula	C_11_H_11_N_5_	C_25_H_23_N_5_O_6_S_2_	C_25_H_23_N_5_O_4_S_2_	C_17_H_14_N_6_O_4_S	C_17_H_14_ClN_5_O_2_S
Formula Mass	213.25	553.60	521.60	398.40	387.84
Crystal system	monoclinic	monoclinic	monoclinic	monoclinic	triclinic
*a*/Å	6.81358(7)	7.5517(2)	9.8874(3)	7.2342(11)	7.6338(8)
*b*/Å	12.04885(14)	17.1382(6)	10.3839(3)	14.748(3)	10.1714(8)
*c*/Å	12.51063(14)	19.2240(6)	23.6423(8)	16.643(2)	11.6609(8)
α/°	90	90	90	90	82.556(6)
β°	96.2273(10)	94.443(3)	97.633(3)	91.217(14)	88.506(7)
ɣ°	90	90	90	90	80.477(8)
Unit cell volume/Å^3^	1021.01(2)	2480.54(13)	2405.83(13)	1775.2(5)	885.41(13)
Space group	*P*2_1_/n	*P*2_1_/c	*P*2_1_/c	*P*2_1_/n	*P*1
Z	4	4	4	4	2
Radiation type	Cu *K*α	Mo *K*α	Mo *K*α	Mo *K*α	Mo *K*α
Absorption coefficient, μ/mm^−1^	0.729	1.482	0.265	0.222	0.356
No. refl. measured, 2Θ_max_/°	7762, 153.3	21,136, 52.7	25,848, 52.7	18,832, 50.1	14,683, 52.7
No. independent reflections	2111	5074	4904	4958	7192
*R*_int_	0.0178	0.0461	0.0486	0.0722	0.0364
Final *R*_1_ values (I > 2σ(I))	0.0340	0.0479	0.0469	0.0432	0.0486
Final wR(F^2^) values (I >2σ(I))	0.0988	0.0943	0.01036	0.0708	0.0769
Final *R*_1_ values (all data)	0.0374	0.0779	0.0755	0.1011	0.0790
Final wR(F^2^) values (all data)	0.1025	0.1074	0.1163	0.0790	0.0899
Goodness of fit on F^2^	1.047	1.037	1.021	0.831	0.995

**Table 2:** Antioxidant activity (ABTS, DPPH, FRAP, and β˗Carotene bleaching) of compounds **6a**–**o** and **7a**–**m** expressed as IC_50_ [µg/mL] with the standard deviation (±SD). Ascorbic acid was used as a positive control.

Compound	ABTS *	DDPH **	FRAP ***	β˗Carotene Bleaching
**6a**	889.22 ± 2.64	NR ****	219.92 ± 1.02	687.5 ± 1.41
**6b**	851.23 ± 2.09	NR	332.73 ± 1.33	191.61 ± 0.55
**6c**	338.63 ± 1.98	NR	419.93 ± 0.6	138.92 ± 0.12
**6d**	682.71 ± 1.87	NR	494.21 ± 0.69	483.1 ± 0.81
**6e**	420.7 ± 1.59	NR	221.6 ± 1.63	391.42 ± 0.32
**6f**	442.57 ± 1.25	NR	273.8 ± 0.83	141.21 ± 0.13
**6g**	558.75 ± 1.51	NR	331.59 ± 1.63	128.21 ± 0.28
**6h**	634.04 ± 0.45	NR	NR	NR
**6i**	483.24 ± 0.33	NR	NR	144.25 ± 1.02
**6j**	**52.77** ± 0.25	NR	NR	**95.24** ± 0.86
**6k**	475.53 ± 0.85	NR	NR	416.5 ± 1.55
**6l**	256.16 ± 1.05	NR	630.72 ± 0.21	413.57 ± 1.02
**6m**	898.49 ± 2.15	891.22 ± 3.67	243.75 ± 0.15	169.96 ± 0.86
**6n**	NR	NR	NR	**97.41** ± 0.28
**6o**	NR	NR	NR	**90.81** ± 1.08
**7a**	NR	NR	NR	NR
**7b**	280.44 ± 1.68	518.26 ± 0.36	246.23 ± 1.6	NR
**7c**	NR	NR	NR	296.27 ± 0.7
**7d**	NR	NR	NR	169.03 ± 0.39
**7e**	657.35 ± 1.59	834.55 ± 2.88	242.8 ± 0.49	312.68 ± 0.22
**7f**	473.01 ± 1.54	799.29 ± 2.57	736.02 ± 1.19	NR
**7g**	847.39 ± 2.58	NR	NR	685.3 ± 1.35
**7h**	588.04 ± 1.25	NR	487.5 ± 1.88	167 ± 0.25
**7i**	391.24 ± 1.25	368.5 ± 1.36	NR	**86.21** ± 0.83
**7j**	536.35 ± 2.77	688.45 ± 2.67	NR	429.01 ± 1.15
**7k**	NR	NR	NR	293.83 ± 0.56
**7l**	NR	NR	NR	NR
**7m**	166.17 ± 1.11	290.63 ± 1.9	NR	NR
Ascorbic acid	15.68 ± 0.8	12.75 ± 0.55	10.17 ± 0.51	49.76 ± 0.67

* ABTS—2,2′-azino-bis(3-ethylbenzothiazoline-6-sulfonic acid. ** DPPH—2,2-diphenyl-1-picrylhydrazyl. *** FRAP test—Ferric ion reducing antioxidant power. **** NR—the IC_50_ value was not reached.

**Table 3:** Representations of the molecular orbitals and their GAP values (energy variations between frontier orbitals HOMO and LUMO) between energy states GAP^1^, GAP^2^, GAP^3^, and GAP^4^ of compounds **6j**, **6k**, **6n**, **6o**, **7h**, and **7i**.

Compound	LUMO{+1}	LUMO	HOMO	HOMO{−1}
**6j**	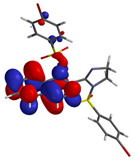	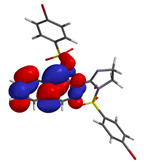	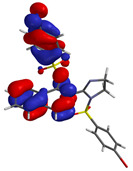	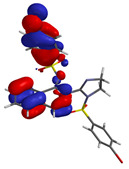
	1.9 eV	1.4 eV	−9.2 eV	−9.6 eV
	^(a)^GAP^1^ = 10.6 eV	^(b)^GAP^2^ = 11.1 eV	^(c)^GAP^3^ = 11.0 eV	^(d)^GAP^4^ = 11.5 eV
**6k**	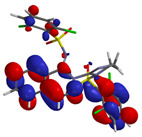	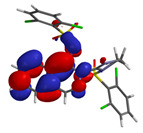	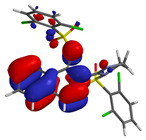	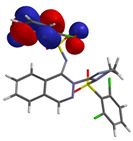
	2.1 eV	1.7 eV	−9.0 eV	−9.3 eV
	GAP^1^ = 10.7 eV	GAP^2^ = 11.1 eV	GAP^3^ = 11.0 eV	GAP^4^ = 11.4 eV
**6n**	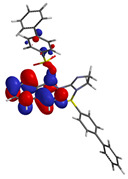	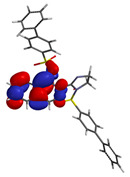	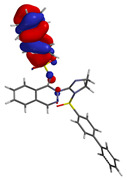	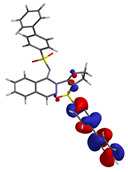
	2.1 eV	1.7eV	−8.4 eV	−8.7 eV
	GAP^1^ = 10.1 eV	GAP^2^ = 10.5 eV	GAP^3^ = 10.4 eV	GAP^4^ = 10.8 eV
**6o**	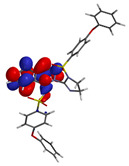	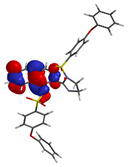	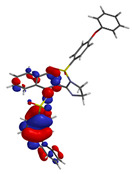	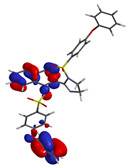
	2.2 eV	1.7 eV	−8.6 eV	−9.1 eV
	GAP^1^ = 10.3 eV	GAP^2^ = 10.8 eV	GAP^3^ = 10.8 eV	GAP^4^ = 11.3 eV
**7h**	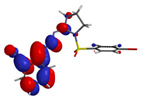	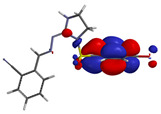	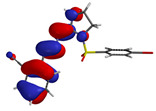	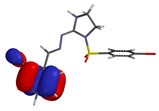
	2.3 eV	1.9 eV	−8.2 eV	−9.3 eV
	GAP^1^ = 10.1 eV	GAP^2^ = 10.5 eV	GAP^3^ = 11.2 eV	GAP^4^ = 11.6 eV
**7i**	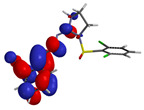	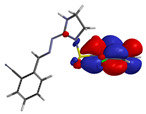	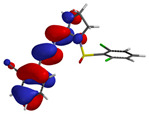	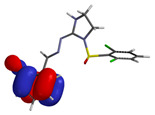
	2.3 eV	1.8 eV	−8.2 eV	−9.2 eV
	GAP^1^ = 10.0 eV	GAP^2^ = 10.5 eV	GAP^3^ = 11.0 eV	GAP^4^ = 11.5 eV

^(a)^GAP^1^ = (LUMO)-(HOMO); ^(b)^GAP^2^ = (LUMO{+1})-(HOMO); ^(c)^GAP^3^ = (LUMO)-(HOMO{−1}); ^(d)^GAP^4^ = (LUMO{+1})-(HOMO{−1}).

**Table 4:** In vitro inhibition of the AGE2-BSA/sRAGE (receptor for advanced glycation end-products) interactions and the investigated compounds **6a**, **6b**, **6g**, **6m**, **7a**, **7c**, **7d**, **7e**, **7f**, **7g**, **7i**, **7j**, and **7k** determined by ELISA assay at a 100 µM concentration. AGE2-BSA is a ligand of RAGE.

Compound		Inhibition
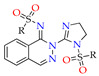	R =	
**6a**	CH_3_	NA *
**6b**	C_6_H_5_	11.0%
**6g**	4-(CH_3_)_3_C-C_6_H_4_	NA
**6m**	2-naphthyl	NA
** 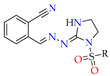 **	R =	
**7a**	CH_3_	NA
**7c**	4-CH_3_-C_6_H_4_	NA
**7d**	3-CH_3_O-C_6_H_4_	36.7%
**7e**	4-(CH_3_)_3_C-C_6_H_4_	NA
**7f**	2-Cl-C_6_H_4_	31.9%
**7g**	4-Cl-C_6_H_4_	NA
**7i**	2,6-di-Cl-C_6_H_3_	NA
**7j**	3,4-di-Cl-C_6_H_3_	NA
**7k**	4-NO_2_-C_6_H_4_	35.6%

* NA—non-active compound.

## Data Availability

Data are contained within the article and Supplementary Materials.

## Supplementary Materials

The following supporting information can be downloaded at: https://www.mdpi.com/article/10.3390/ijms252111495/s1.
