# Supplementary material for: Synthesis and Structure of Novel Hybrid Compounds Containing Phthalazin-1(2H)-imine and 4,5-Dihydro-1H-imidazole Cores and Their Sulfonyl Derivatives with Potential Biological Activities
Source: Int J Mol Sci. 2024 Oct 26;25(21):11495. doi: 10.3390/ijms252111495 (PMC11546079; doi:10.3390/ijms252111495)

SUPPLEMENTARY MATERIALS

Synthesis and Structure of Novel Hybrid Compounds Containing Phthalazin-1(2*H*)-imine and 4,5-Dihydro-1*H*-imidazole Cores and their Sulfonyl Derivatives with Potential Biological Activities

Łukasz Balewski [1,*], Maria Gdaniec [2], Anna Hering [3], Christophe Furman [4], Alina Ghinet [4,5], Jakub Kokoszka [1], Anna Ordyszewska [6], and Anita Kornicka [1,*]

[1] Department of Chemical Technology of Drugs, Faculty of Pharmacy, Medical University of Gdansk, Gen. J. Hallera 107, 80-416 Gdańsk, Poland; lukasz.balewski@gumed.edu.pl (Ł.B.); anita.kornicka@gumed.edu.pl (A.K); jakub.kokoszka@gumed.edu.pl (J.K.)
[2] Faculty of Chemistry, Adam Mickiewicz University, 61-614 Poznań, Poland; maria.gdaniec@amu.edu.pl (M.G.)
[3] Department of Biology and Pharmaceutical Botany, Faculty of Pharmacy, Medical University of Gdansk, Gen. J. Hallera 107, 80-416 Gdańsk, Poland; anna.hering@gumed.edu.pl (A.H.)
[4] Univ. Lille, Inserm, CHU Lille, Institut Pasteur de Lille, UMR 1167 – RID-AGE – Risk Factors and Molecular Determinants of Aging-Related Diseases, F-59000 Lille, France; christophe.furman@univ-lille2.fr (C.F.)
[5] Junia, Health and Environment, Laboratory of Sustainable Chemistry and Health, F-59000, Lille, France; alina.ghinet@junia.com
[6] Department of Inorganic Chemistry, Faculty of Chemistry and Advanced Materials Centers, Gdańsk University of Technology, Narutowicza 11/12, 80-233 Gdansk, Poland; anna.ordyszewska@pg.edu.pl (A.O.)

* Correspondence: lukasz.balewski@gumed.edu.pl (Ł.B.); anita.kornicka@gumed.edu.pl (A.K.)

TABLE OF CONTENTS

1. *Spectra of 2-(4,5-dihydro-1H-imidazol-2-yl)phthalazin-1(2H)-imine (5)*

   Figure S1. IR spectrum of 2-(4,5-dihydro-1*H*-imidazol-2-yl)phthalazin-1(2*H*)-imine (**5**).

   Figure S2. ^{1}H NMR (500 MHz, DMSO-d_6) of 2-(4,5-dihydro-1*H*-imidazol-2-yl)phthalazine-1(2*H*)-imine (**5**).

   Figure S3. ^{13}C NMR (125 MHz, DMSO-d_6) of 2-(4,5-dihydro-1*H*-imidazol-2-yl)phthalazin-1(2*H*)-imine (**5**).

   Figure S4. Heteronuclear single quantum coherence (HSQC) of 2-(4,5-dihydro-1*H*-imidazol-2-yl)phthalazin-1(2*H*)-imine (**5**).

   Figure S5. Heteronuclear multiple bond correlation (HMBC) spectrum of 2-(4,5-dihydro-1*H*-imidazol-2-yl)phthalazin-1(2*H*)-imine (**5**).

   Figure S6. ^{1}H-^{1}H ROESY - rotating frame Overhauser enhancement spectrum of of 2-(4,5-dihydro-1*H*-imidazol-2-yl)phthalazin-1(2*H*)-imine (**5**).

   Figure S7. MS spectrum of 2-(4,5-dihydro-1*H*-imidazol-2-yl)phthalazin-1(2*H*)-imine (**5**).

2. *Spectra of Representative Compounds 6a, 7a, and 7k*

   Figure S8. IR spectrum of *N*-(2-(1-(methylsulfonyl)-4,5-dihydro-1*H*-imidazol-2-yl)phthalazin-1(2*H*)-ylidene)methanesulfonamide (**6a**).

   Figure S9. ^{1}H NMR (400 MHz, DMSO-d_6) spectrum of *N*-(2-(1-(methylsulfonyl)-4,5-dihydro-1*H*-imidazol-2-yl)phthalazin-1(2*H*)-ylidene)methanesulfonamide (**6a**).

Figure S10. ^{13}C NMR (100 MHz, DMSO-d_6) spectrum of *N*-(2-(1-(methylsulfonyl)-4,5-dihydro-1*H*-imidazol-2-yl)phthalazin-1(2*H*)-ylidene)methanesulfonamide (**6a**).
Figure S11. MS spectrum of *N*-(2-(1-(methylsulfonyl)-4,5-dihydro-1*H*-imidazol-2-yl)phthalazin-1(2*H*)-ylidene)methanesulfonamide (**6a**).
Figure S12. IR spectrum of 2-(((1-(methylsulfonyl)imidazolidin-2-ylidene)hydrazono)methyl)benzonitrile (**7a**).
Figure S13. ^{1}H NMR (400 MHz, DMSO-d_6) spectrum of 2-(((1-(methylsulfonyl)imidazolidin-2-ylidene)hydrazono)methyl)benzonitrile (**7a**).
Figure S14. ^{13}C NMR (100 MHz, DMSO-d_6) spectrum of 2-(((1-(methylsulfonyl)imidazolidin-2-ylidene)hydrazono)methyl)benzonitrile (**7a**).
Figure S15. MS spectrum of 2-(((1-(methylsulfonyl)imidazolidin-2-ylidene)hydrazono)methyl)benzonitrile (**7a**).
Figure S16. IR spectrum of 2-(((1-((4-nitrophenyl)sulfonyl)imidazolidin-2-ylidene)hydrazono)methyl)benzonitrile (**7k**).
Figure S17. ^{1}H NMR (400 MHz, DMSO-d_6) spectrum of 2-(((1-((4-nitrophenyl)sulfonyl)imidazolidin-2-ylidene)hydrazono)methyl)benzonitrile (**7k**).
Figure S18. ^{13}C NMR (100 MHz, DMSO-d_6) spectrum of 2-(((1-((4-nitrophenyl)sulfonyl)imidazolidin-2-ylidene)hydrazono)methyl)benzonitrile (**7k**).
Figure S19. MS spectrum of 2-(((1-((4-nitrophenyl)sulfonyl)imidazolidin-2-ylidene)hydrazono)methyl)benzonitrile (**7k**).

3. *X-ray Crystallographic Studies*

Figure S20. CheckCIF/PLATON report for 2-(4,5-dihydro-1*H*-imidazol-2-yl)phthalazin-1(2*H*)-imine (**5**).
Figure S21. CheckCIF/PLATON report for 4-methyl-*N*-(2-(1-tosyl-4,5-dihydro-1*H*-imidazol-2-yl)phthalazin-1(2*H*)-ylidene)benzenesulfonamide (**6c**).
Figure S22. CheckCIF/PLATON report for 4-methoxy-*N*-(2-(1-((4-methoxyphenyl)sulfonyl)-4,5-dihydro-1*H*-imidazol-2-yl)phthalazin-1(2*H*)-ylidene)benzenesulfonamide (**6e**).
Figure S23. CheckCIF/PLATON report for 2-(((1-((4-chlorophenyl)sulfonyl)imidazolidin-2-ylidene)hydrazono)methyl)benzonitrile (**7g**).
Figure S24. CheckCIF/PLATON report for 2-(((1-((4-nitrophenyl)sulfonyl)imidazolidin-2-ylidene)hydrazono)methyl)benzonitrile (**7k**).
Figure S25. Crystal packing in **6e** and **6c**.
Figure S26. Centrosymmetric dimer via π-π stacking interactions in **6c**.
Figure S27. Crystal packing in **7g** and **7k**.

Spectra of 2-(4,5-dihydro-1H-imidazol-2-yl)phthalazin-1(2H)-imine (5)

Figure S1. IR spectrum of 2-(4,5-dihydro-1*H*-imidazol-2-yl)phthalazin-1(2*H*)-imine (**5**).

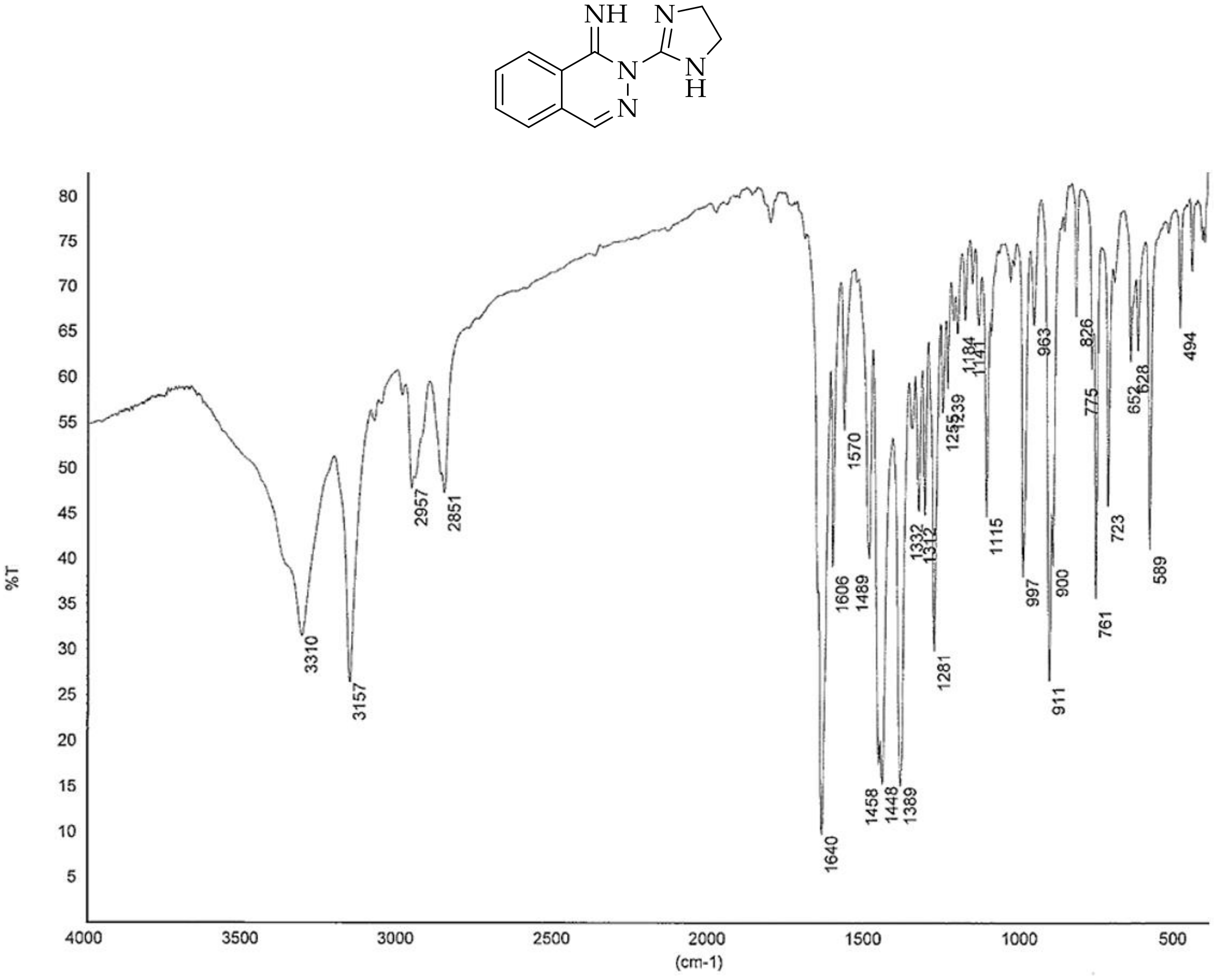

Figure S2. ^{1}H NMR (500 MHz, DMSO-d_6) of 2-(4,5-dihydro-1*H*-imidazol-2-yl)phthalazin-1(2*H*)-imine (**5**).

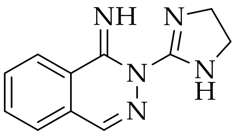

Solvent: dmso
Ambient temperature
File: TCA_172_1H
INOVA-500

Total time 15 min

INDEX	FREQUENCY	PPM	HEIGHT	INDEX	FREQUENCY	PPM	HEIGHT
1	5434.9	10.874	5.4	17	1938.6	3.879	8.2
2	4175.7	8.355	7.8	18	1680.5	3.362	49.2
3	4173.5	8.350	9.7	19	1662.4	3.326	8.1
4	4170.2	8.344	5.0	20	1248.9	2.499	5.2
5	4165.8	8.335	11.6	21	1247.2	2.495	6.4
6	4054.4	8.112	37.0	22	1245.0	2.491	4.9
7	3861.1	7.725	8.6				
8	3856.7	7.716	26.2				
9	3854.5	7.712	47.2				
10	3852.3	7.708	22.3				
11	3850.6	7.704	12.7				
12	3848.4	7.700	15.7				
13	3846.8	7.697	10.1				
14	3844.6	7.692	5.2				
15	3842.4	7.688	4.4				
16	3470.1	6.943	5.1				

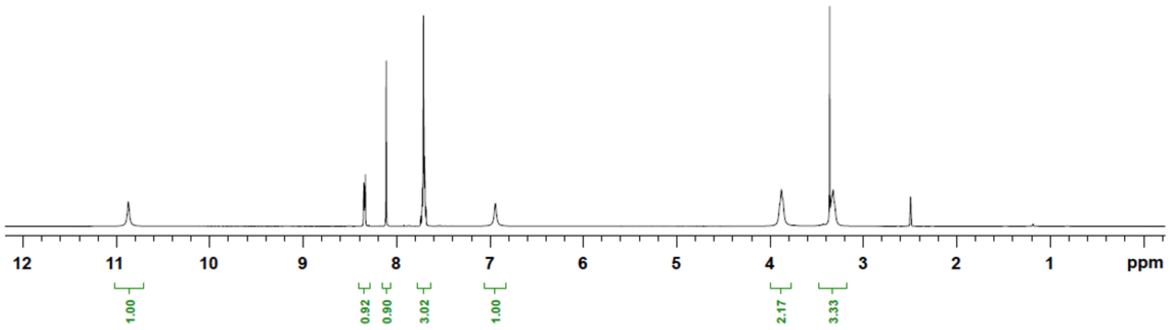

Figure S3. ^{13}C NMR (125 MHz, DMSO-d_6) of 2-(4,5-dihydro-1*H*-imidazol-2-yl)phthalazin-1(2*H*)-imine (**5**).

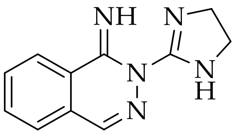

Solvent: dmso
Ambient temperature
File: TCA_172_13C
INOVA-500

Total time 15 min

INDEX	FREQUENCY	PPM	HEIGHT	INDEX	FREQUENCY	PPM	HEIGHT
1	20315.2	161.648	16.1	17	4981.2	39.635	25.5
2	18588.2	147.907	15.7	18	4960.1	39.467	8.4
3	17383.8	138.323	49.3				
4	16677.1	132.700	41.2				
5	16650.3	132.486	38.7				
6	16320.4	129.861	15.4				
7	15998.2	127.298	49.8				
8	15804.5	125.756	53.1				
9	15729.7	125.161	23.0				
10	6808.9	54.178	5.7				
11	5385.8	42.855	5.9				
12	5085.7	40.467	9.8				
13	5064.6	40.299	28.0				
14	5043.5	40.131	54.5				
15	5022.4	39.963	62.5				
16	5001.3	39.795	51.7				

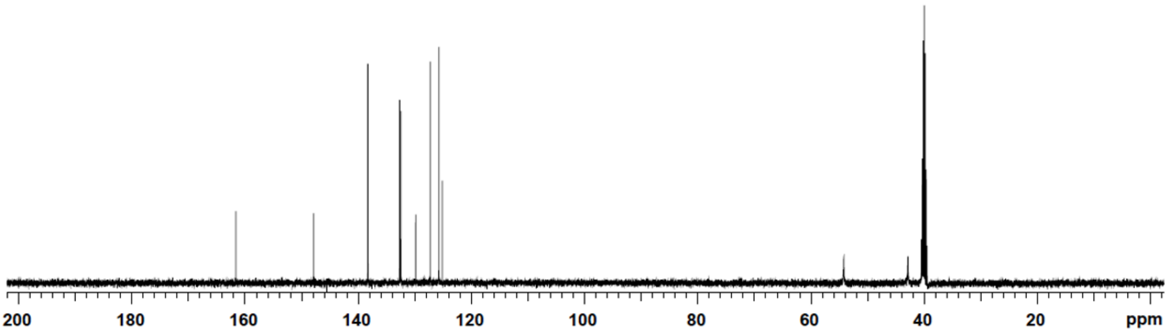

Figure S4. Heteronuclear single quantum coherence (HSQC) of 2-(4,5-dihydro-1*H*-imidazol-2-yl)phthalazin-1(2*H*)-imine (**5**).

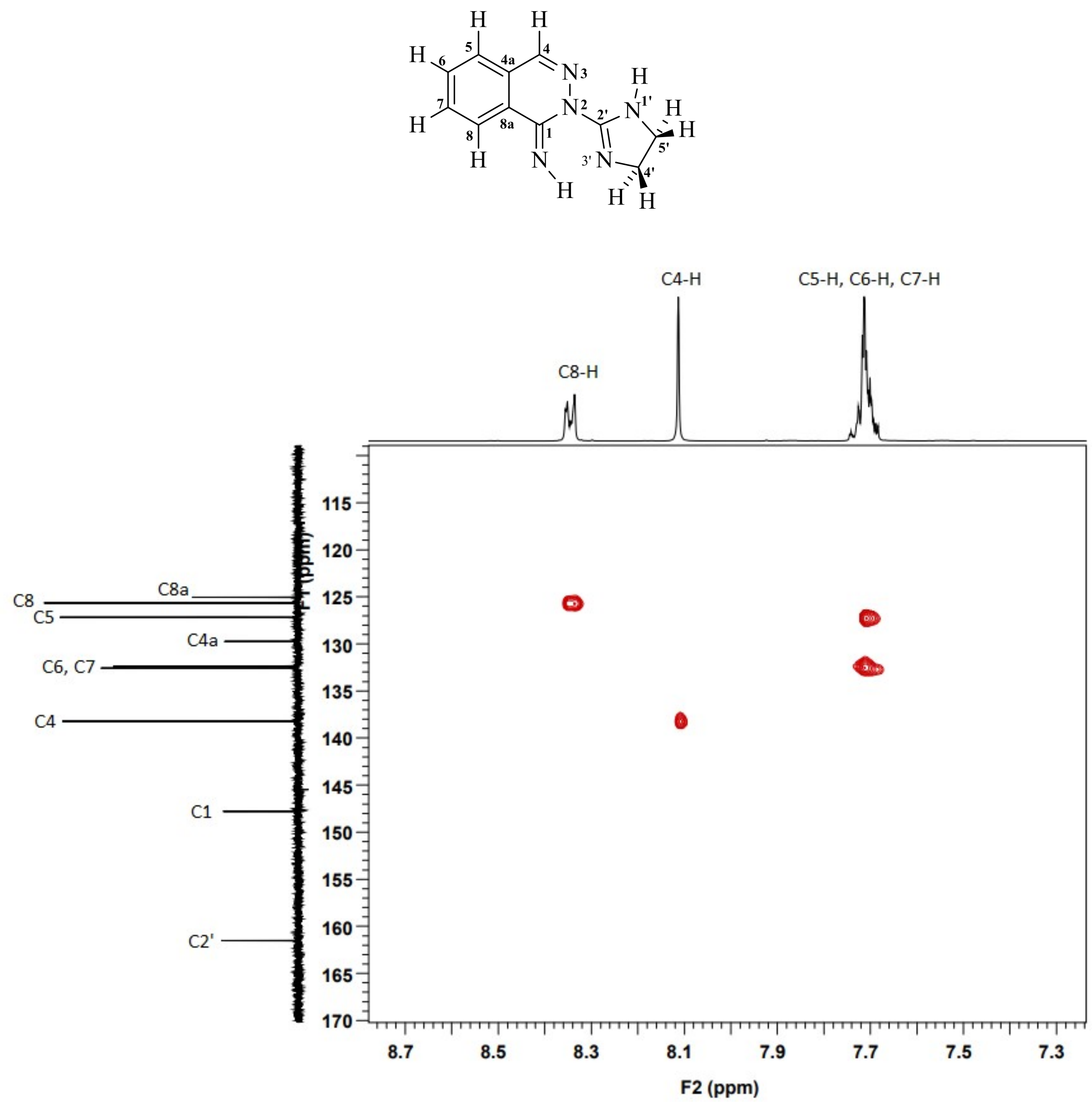

Figure S5. Heteronuclear multiple bond correlation (HMBC) spectrum of 2-(4,5-dihydro-1*H*-imidazol-2-yl)phthalazin-1(2*H*)-imine (**5**).

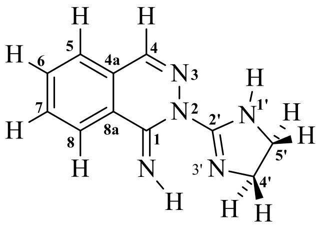

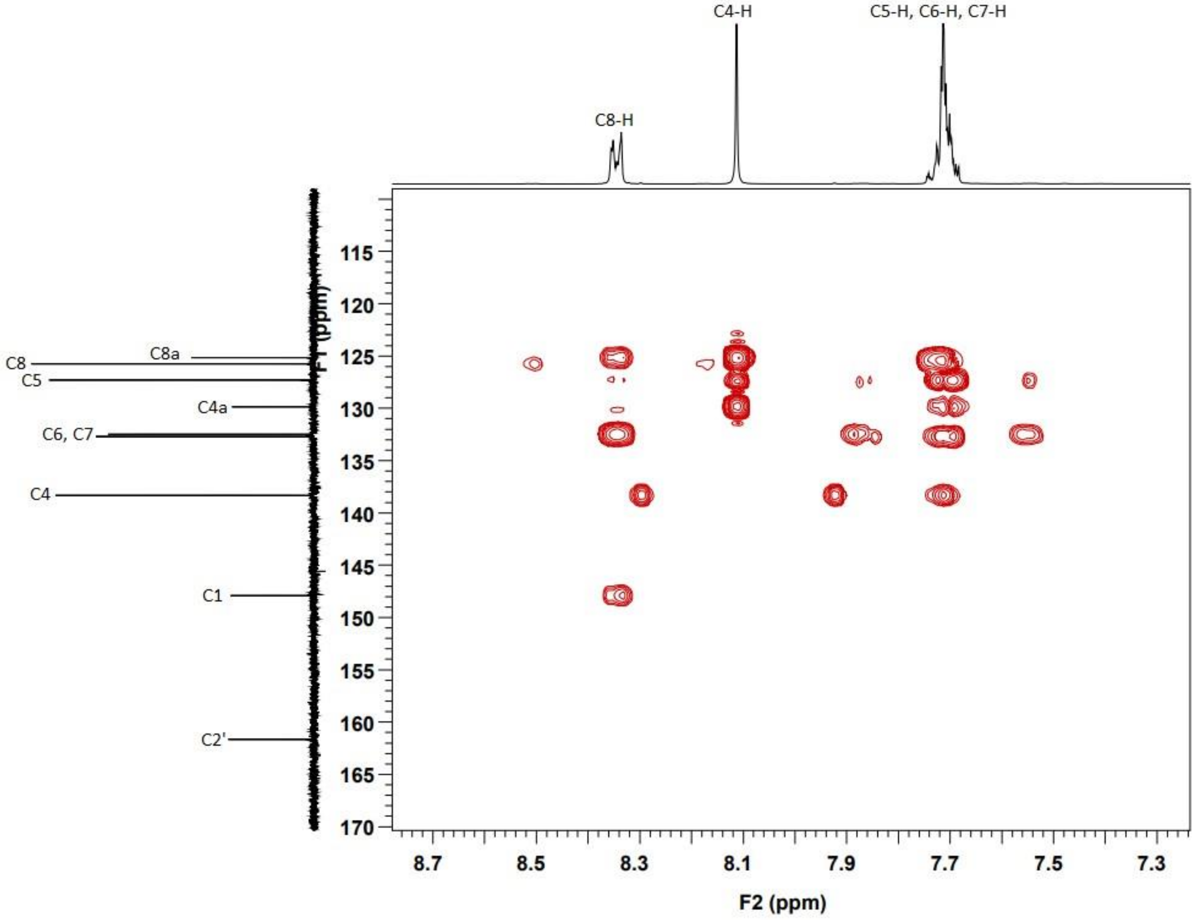

Figure S6. ^{1}H-^{1}H ROESY (Rotating-Frame Overhauser Enhancement Spectroscopy) spectrum of 2-(4,5-dihydro-1*H*-imidazol-2-yl)phthalazin-1(2*H*)-imine (**5**).

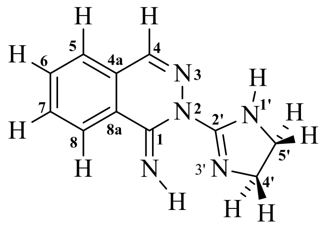

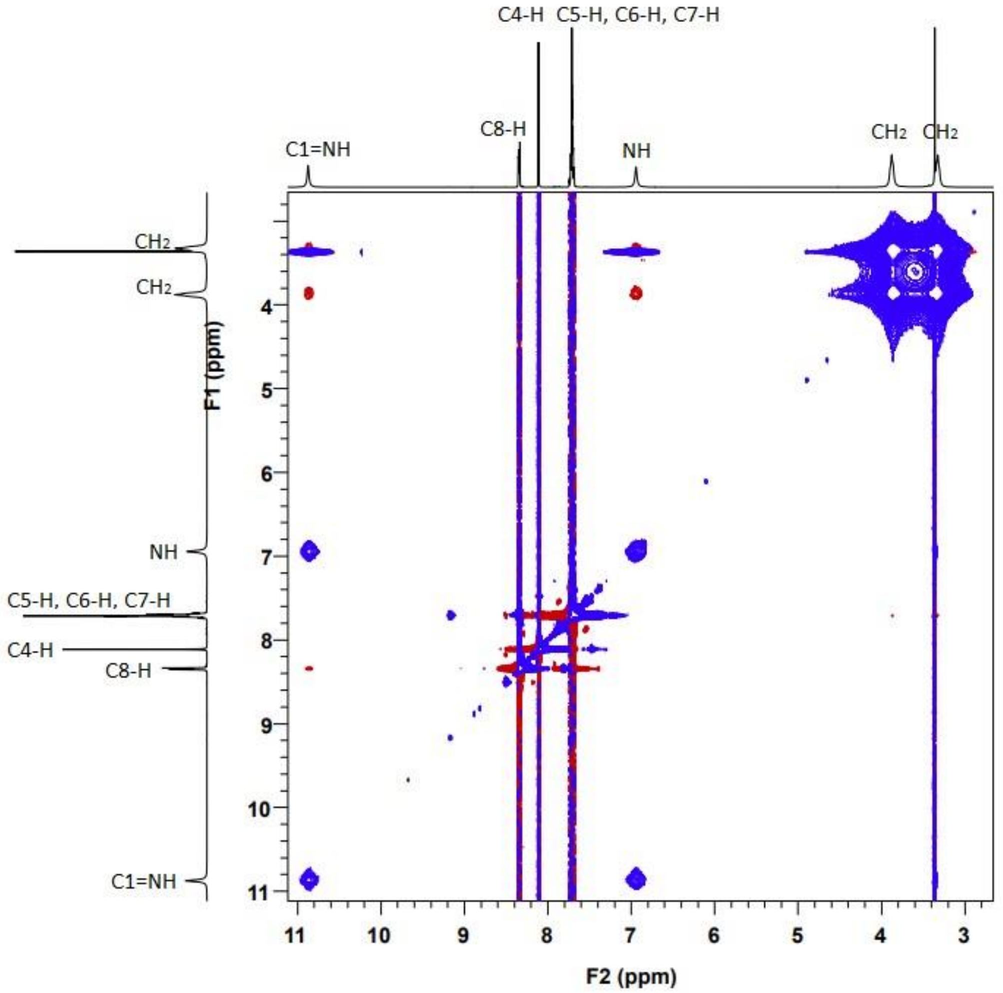

Figure S7. MS spectrum of of 2-(4,5-dihydro-1*H*-imidazol-2-yl)phthalazin-1(2*H*)-imine (**5**).

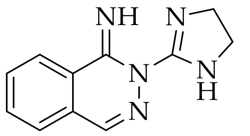

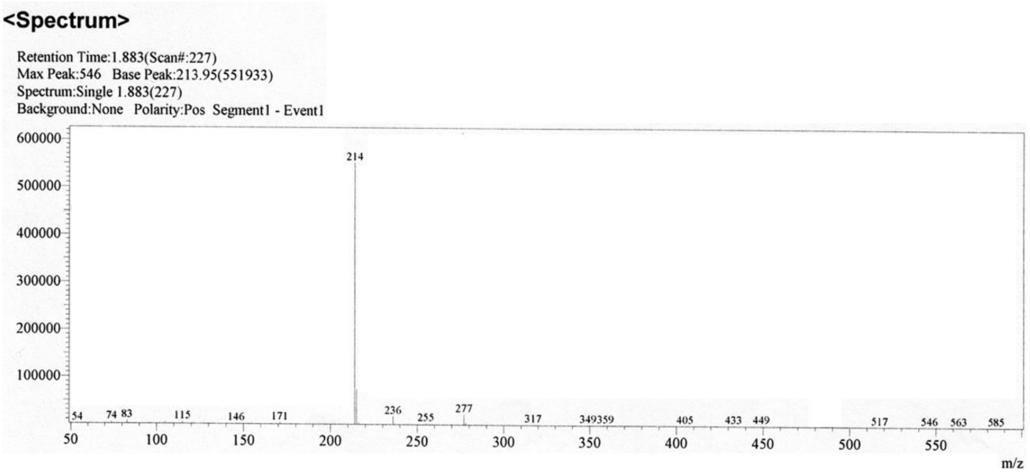

Figure S8. IR spectrum of *N*-(2-(1-(methylsulfonyl)-4,5-dihydro-1*H*-imidazol-2-yl)phthalazin-1(2*H*)-ylidene)methanesulfonamide (**6a**).

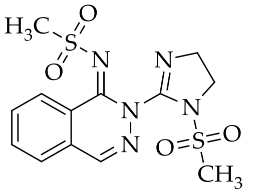

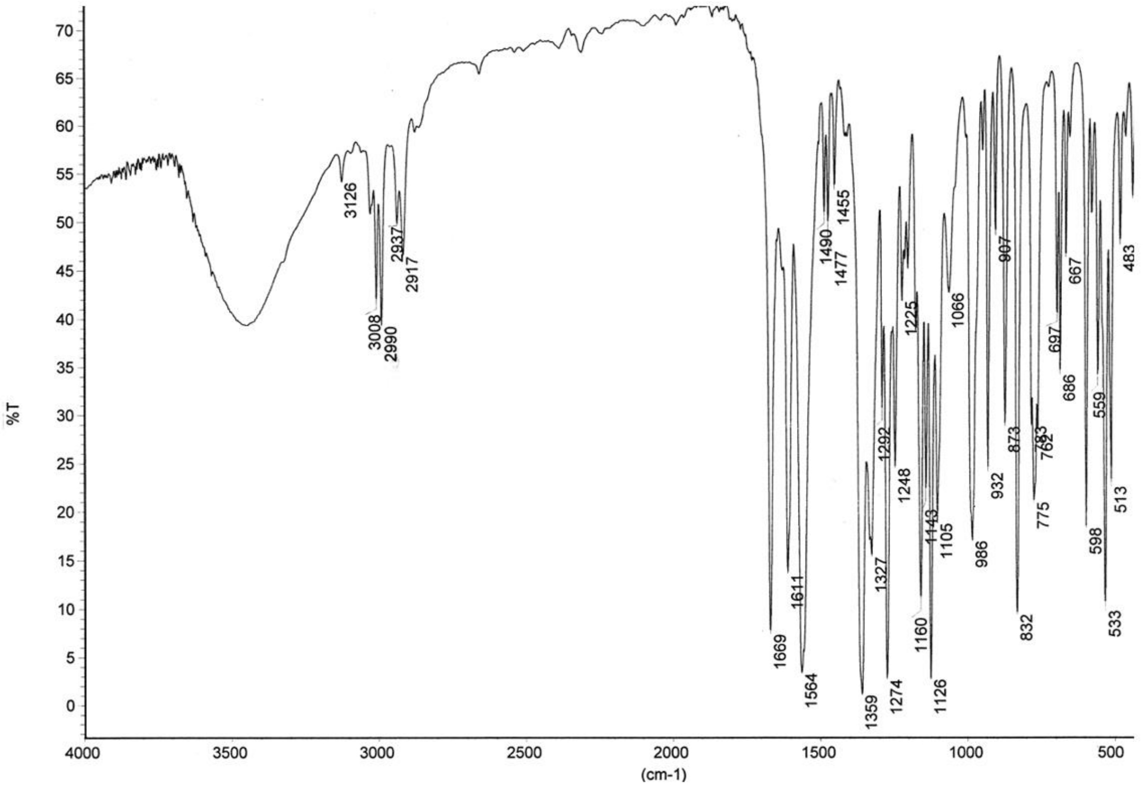

Figure S9. ^{1}H NMR (400 MHz, DMSO-d_6) spectrum of *N*-(2-(1-(methylsulfonyl)-4,5-dihydro-1*H*-imidazol-2-yl)phthalazin-1(2*H*)-ylidene)methanesulfonamide (**6a**).

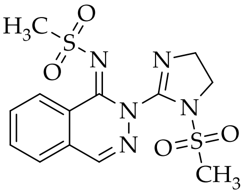

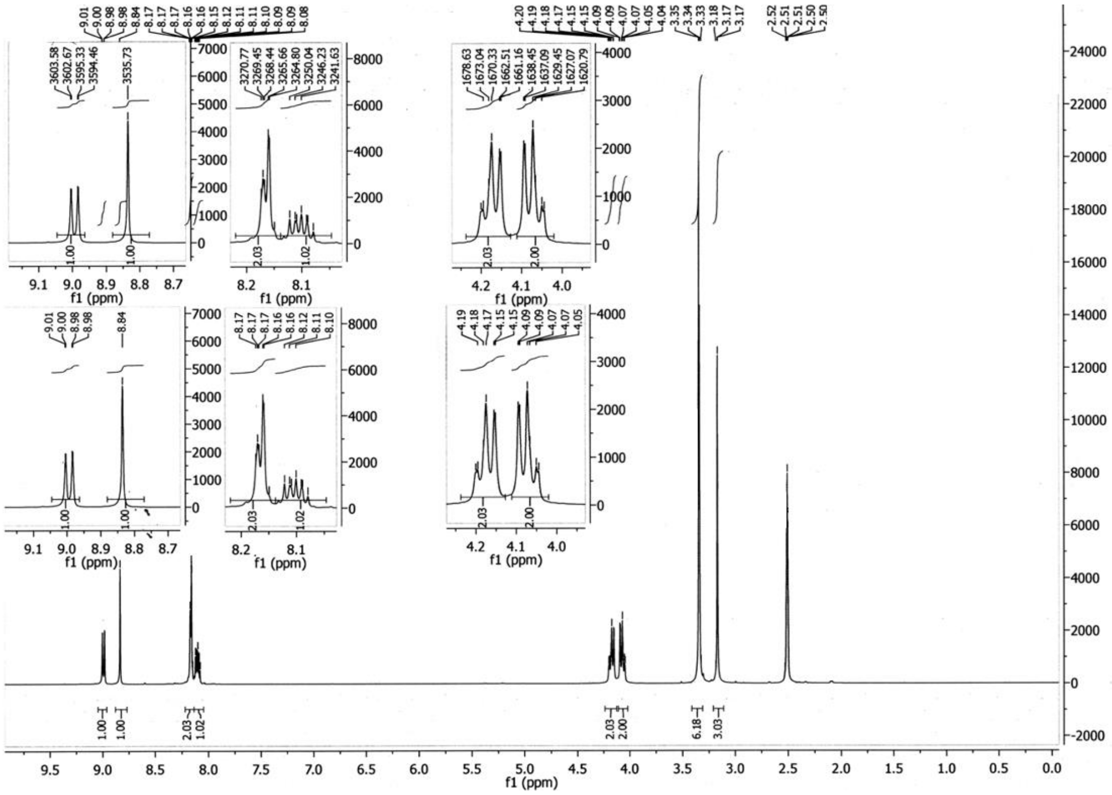

Figure S10. ^{13}C NMR (100 MHz, DMSO-d_6) spectrum of *N*-(2-(1-(methylsulfonyl)-4,5-dihydro-1*H*-imidazol-2-yl)phthalazin-1(2*H*)-ylidene)methanesulfonamide (**6a**).

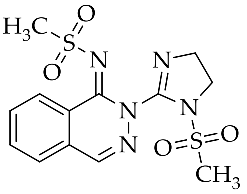

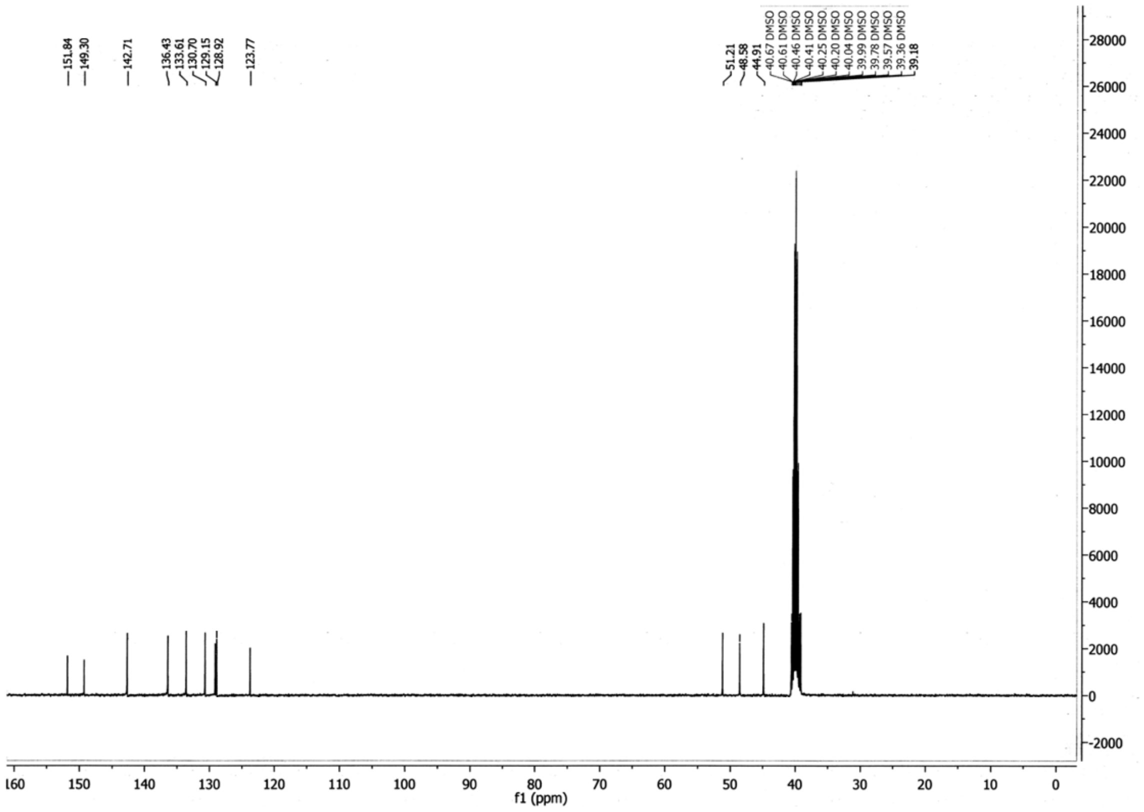

Figure S11. MS spectrum of *N*-(2-(1-(methylsulfonyl)-4,5-dihydro-1*H*-imidazol-2-yl)phthalazin-1(2*H*)-ylidene)methanesulfonamide (**6a**).

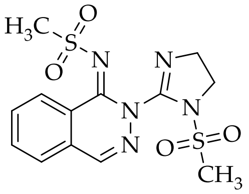

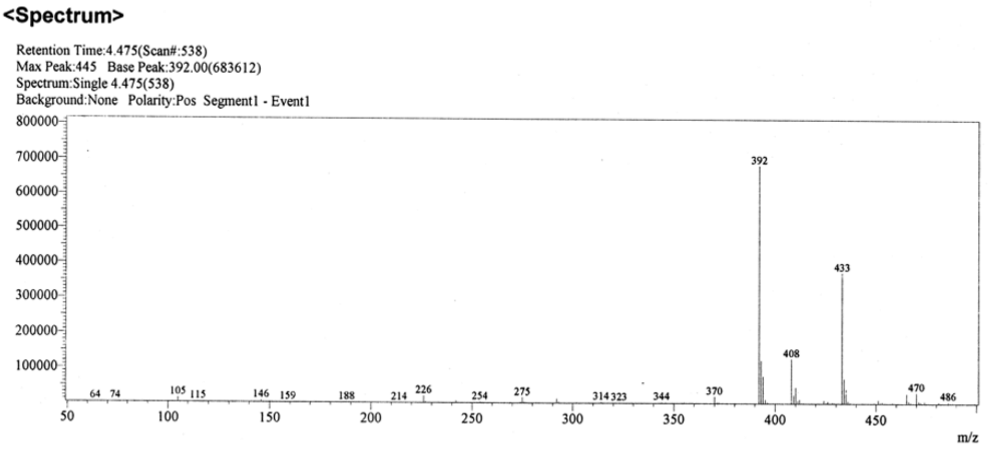

Figure S12. IR spectrum of 2-(((1-(methylsulfonyl)imidazolidin-2-ylidene)hydrazono)methyl)benzonitrile (**7a**).

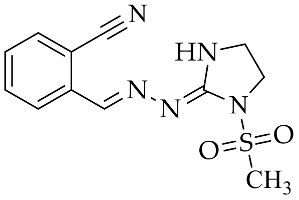

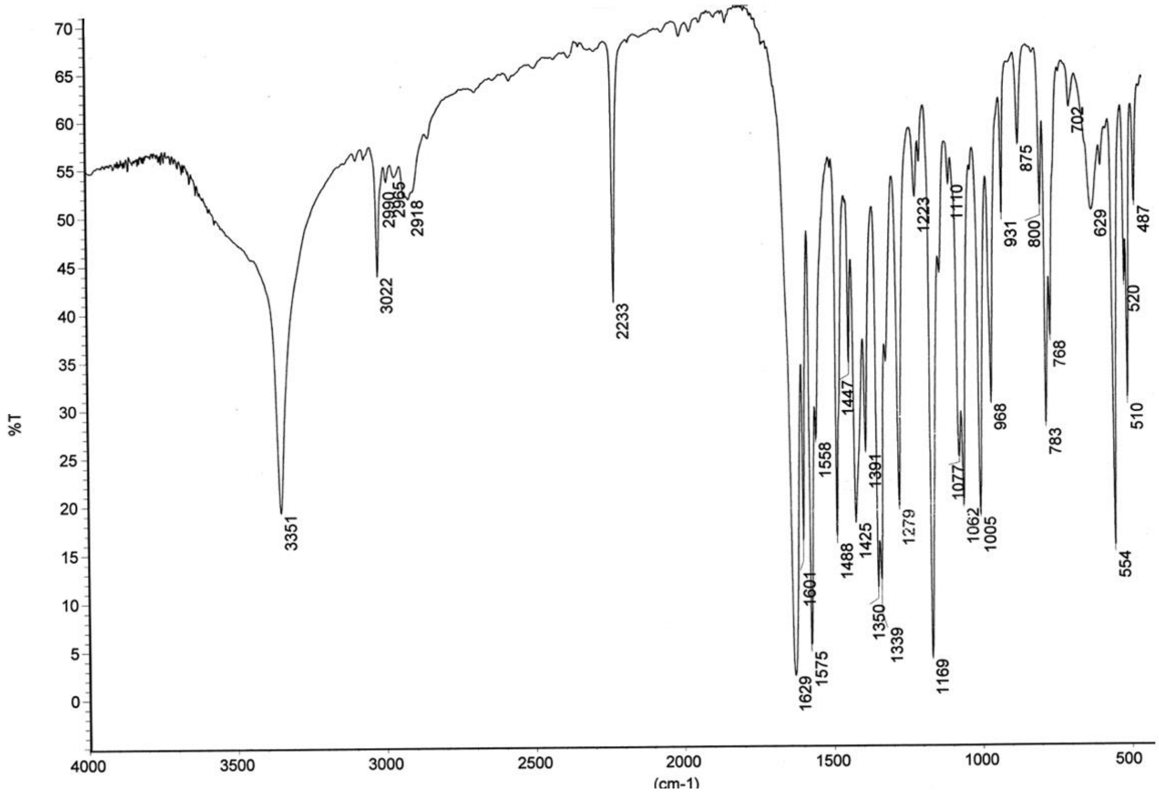

Figure S13. ^{1}H NMR (400 MHz, DMSO-*d*6) spectrum of 2-(((1-(methylsulfonyl)imidazolidin-2-ylidene)hydrazono)methyl)benzonitrile (**7a**).

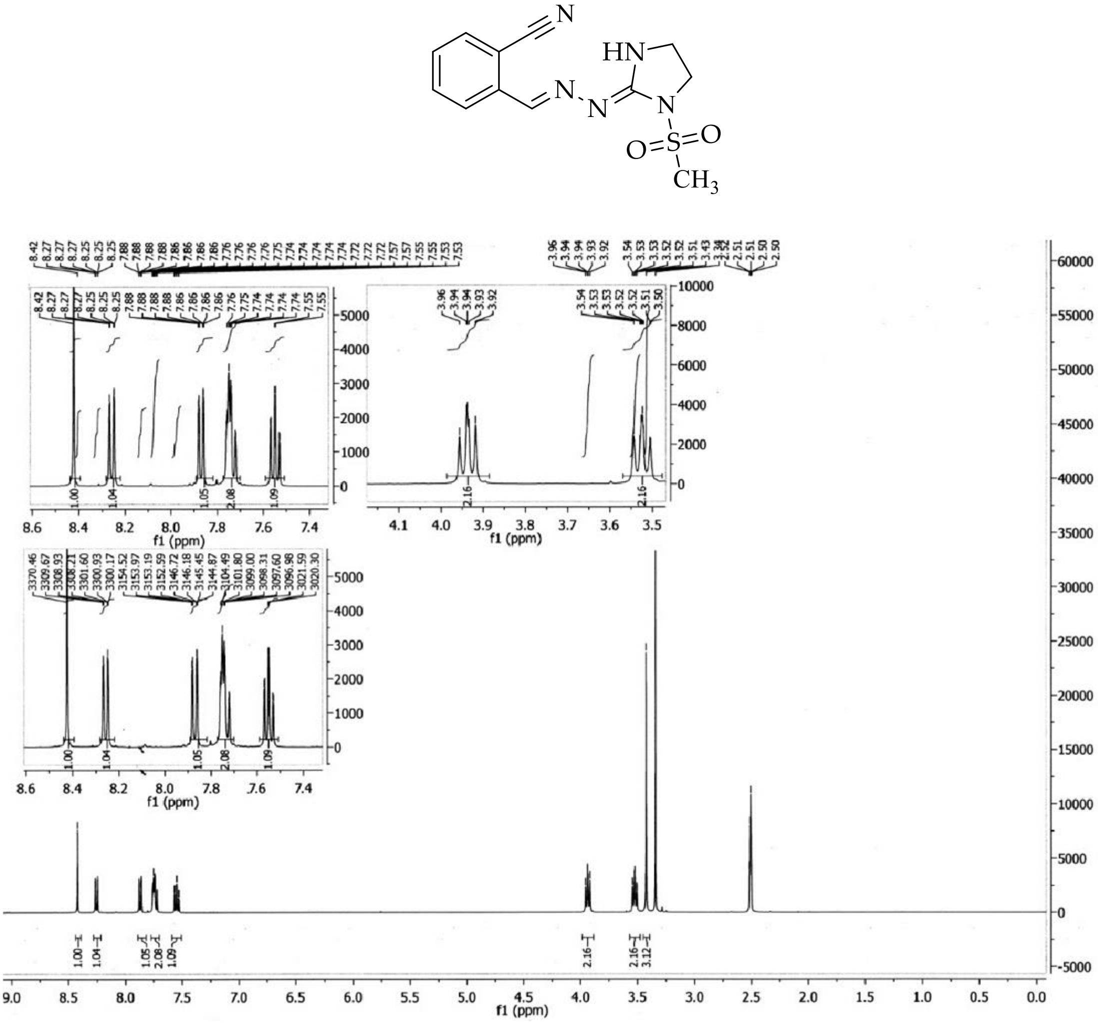

Figure S14. ^{13}C NMR (100 MHz, DMSO-d_6) spectrum of 2-(((1-(methylsulfonyl)imidazolidin-2-ylidene)hydrazono)methyl)benzonitrile (**7a**).

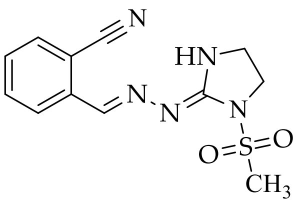

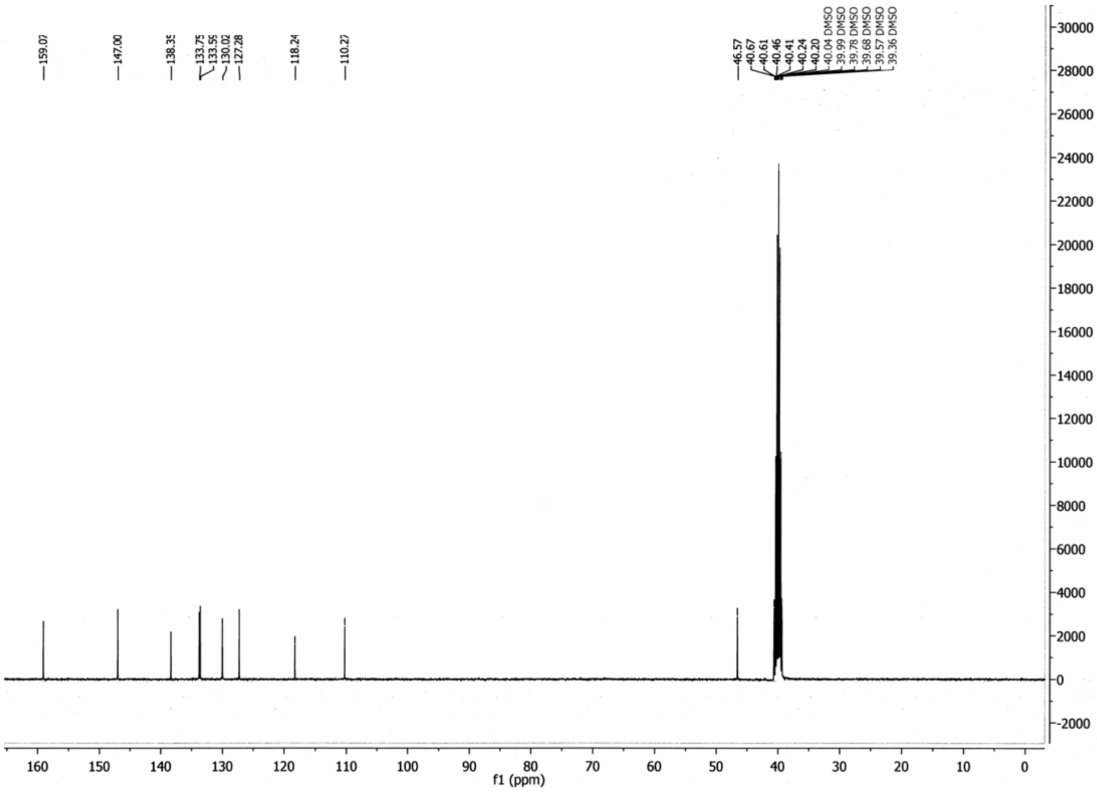

Figure S15. MS spectrum of 2-(((1-(methylsulfonyl)imidazolidin-2-ylidene)hydrazono)methyl)benzonitrile (**7a**).

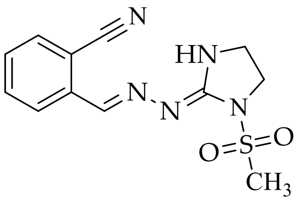

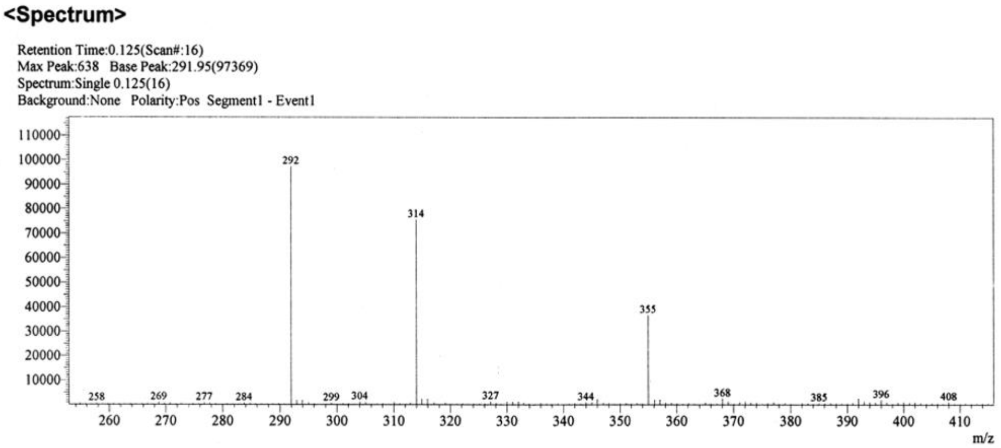

Figure S16. IR spectrum of 2-(((1-((4-nitrophenyl)sulfonyl)imidazolidin-2-ylidene)hydrazono)methyl)benzonitrile (**7k**).

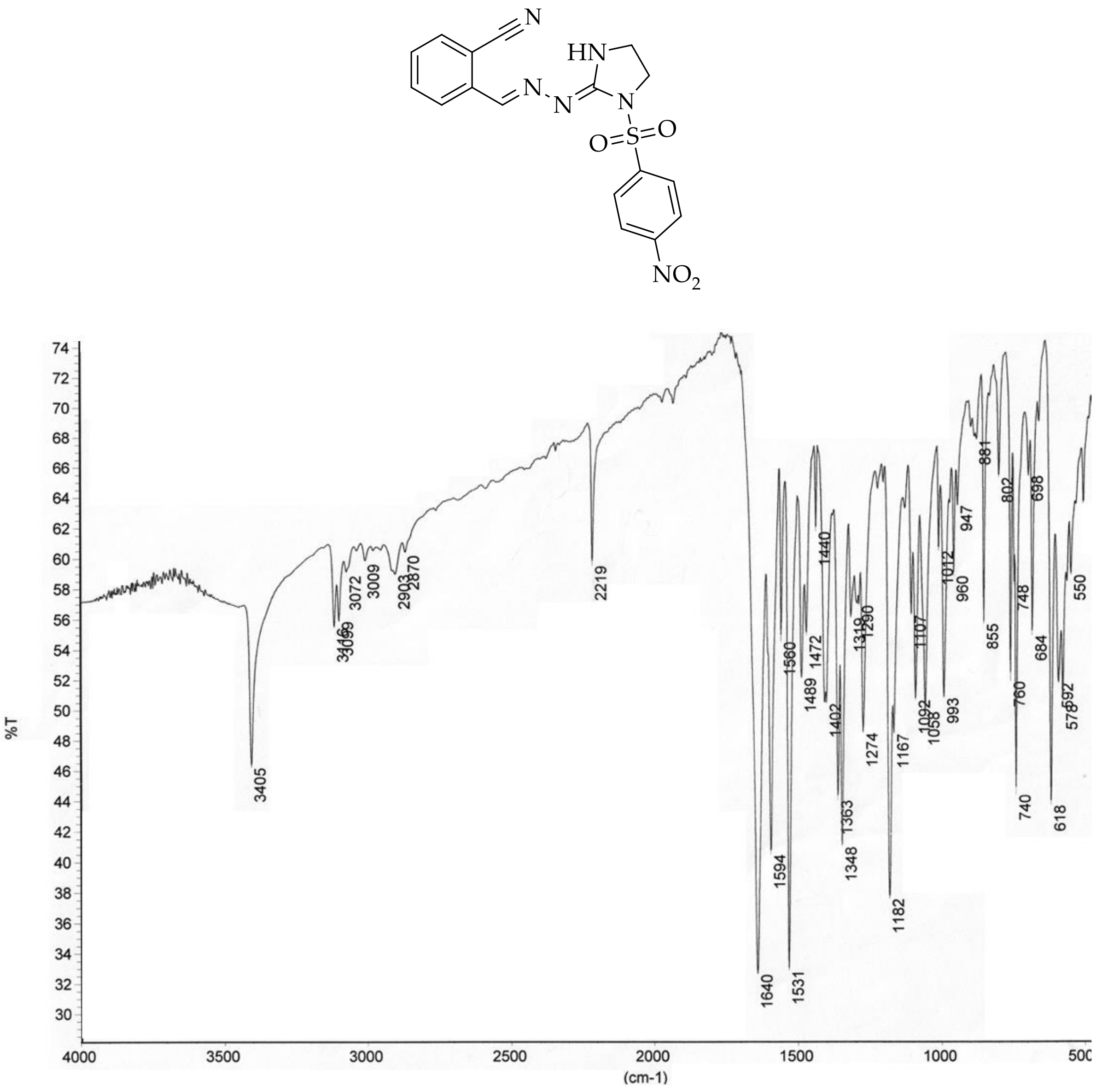

Figure S17. ^{1}H NMR (400 MHz, DMSO-d_6) spectrum of 2-(((1-((4-nitrophenyl)sulfonyl)imidazolidin-2-ylidene)hydrazono)methyl)benzonitrile (**7k**).

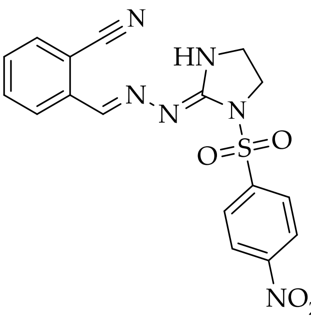

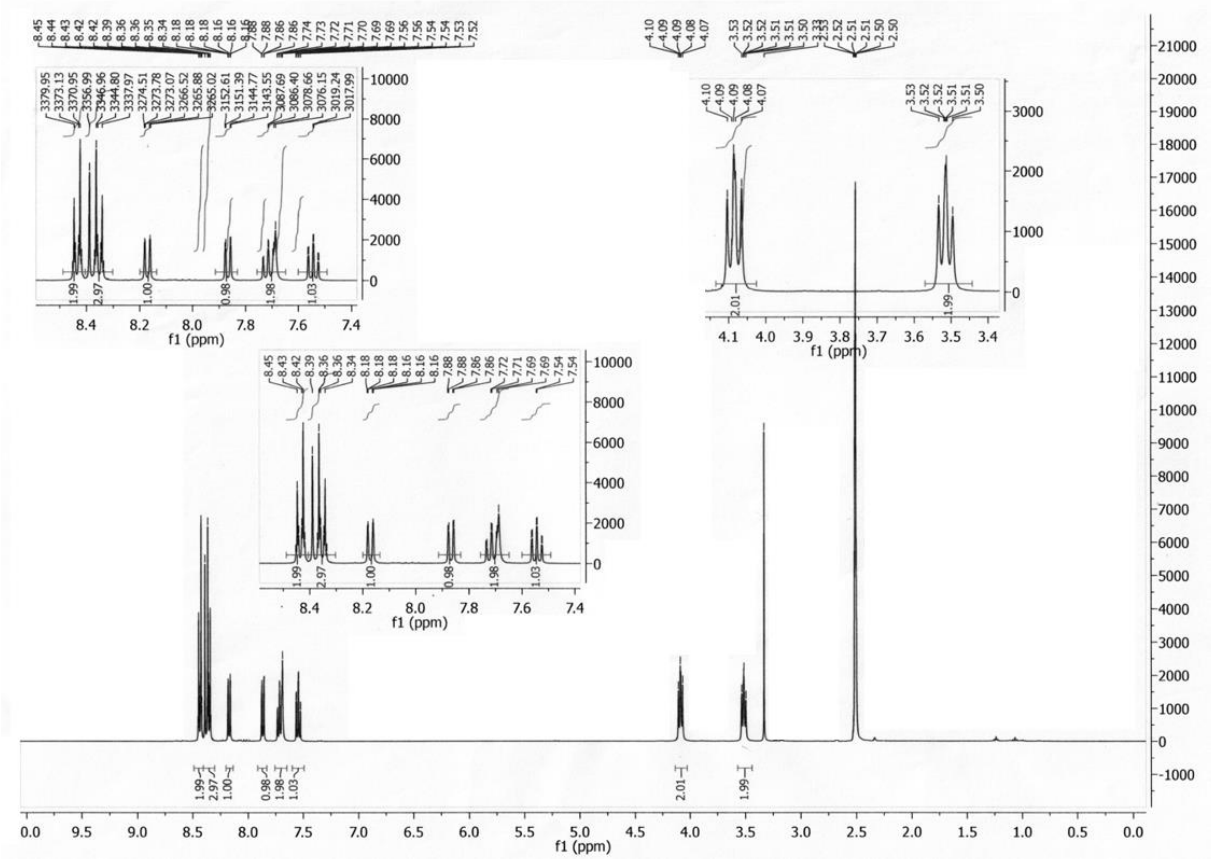

Figure S18. ^{13}C NMR (100 MHz, DMSO-d_6) spectrum of 2-(((1-((4-nitrophenyl)sulfonyl)imidazolidin-2-ylidene)hydrazono)methyl)benzonitrile (**7k**).

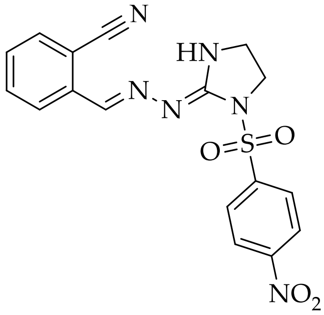

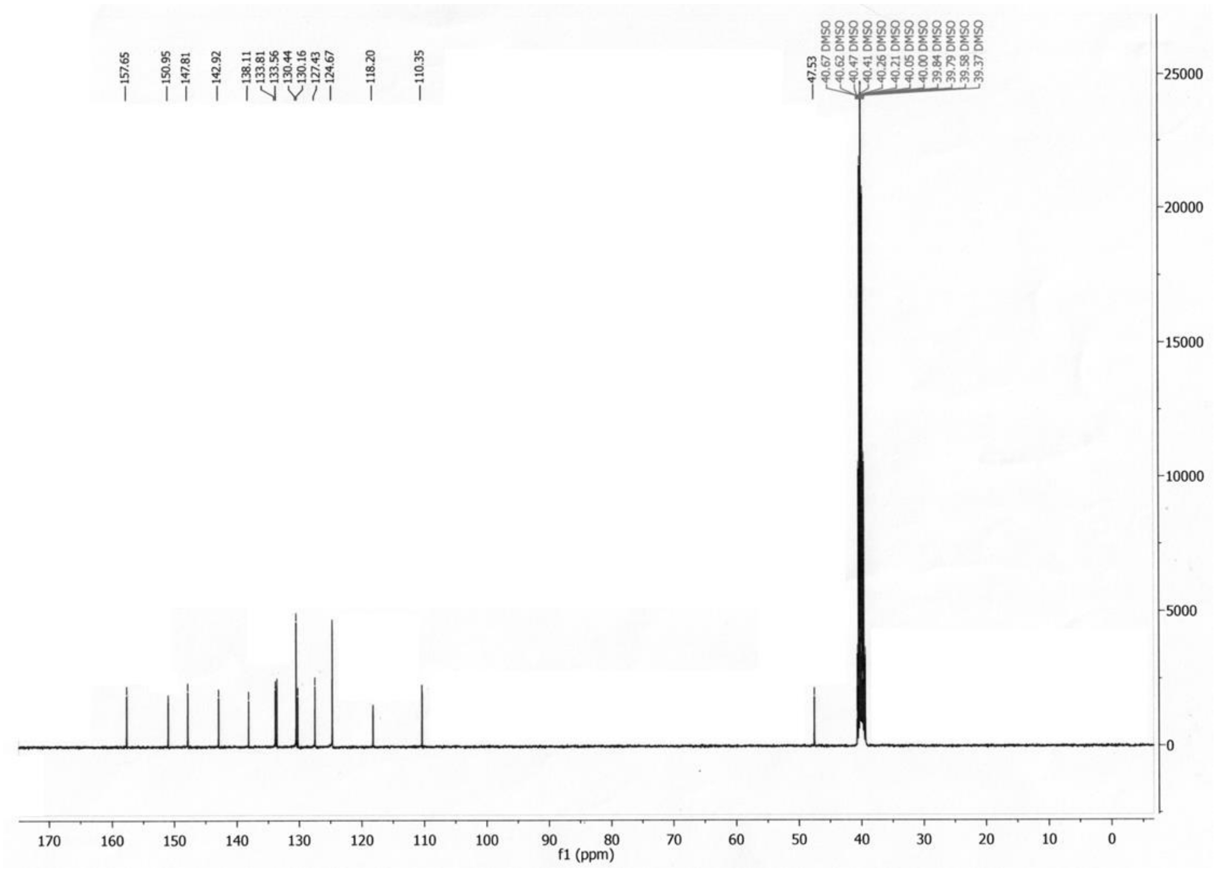

Figure S19. MS spectrum of 2-(((1-((4-nitrophenyl)sulfonyl)imidazolidin-2-ylidene)hydrazono)methyl)benzonitrile (**7k**).

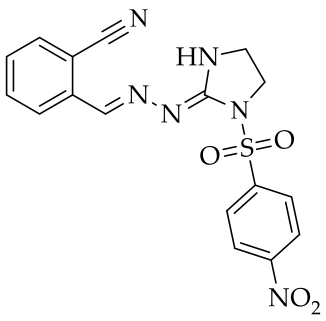

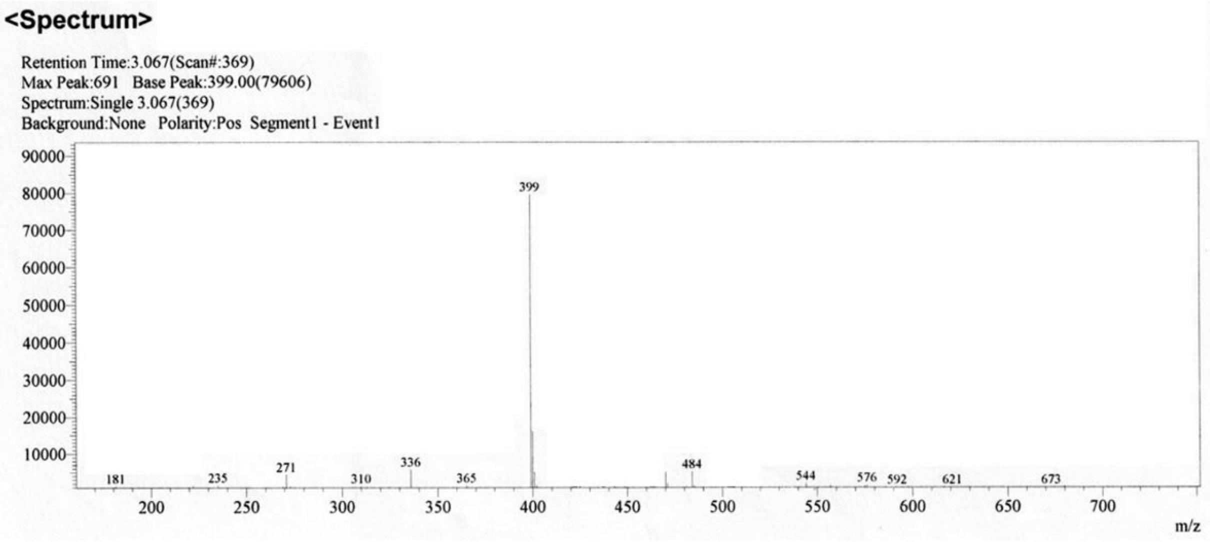

X-ray Crystallographic Studies

Figure S20. CheckCIF/PLATON report for 2-(4,5-dihydro-1*H*-imidazol-2-yl)phthalazin-1(2*H*)-imine (**5**).

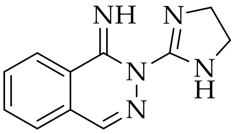


```
Bond precision:   C-C = 0.0017 A          Wavelength=1.54184

Cell:             a=6.81358(7)   b=12.04885(14)    c=12.51063(14)
                  alpha=90       beta=96.2273(10)  gamma=90
Temperature:      293 K

                  Calculated                 Reported
Volume            1021.01(2)                 1021.01(2)
Space group       P 21/n                     P 1 21/n 1
Hall group        -P 2yn                     -P 2yn
Moiety formula    C11 H11 N5                 C11 H11 N5
Sum formula       C11 H11 N5                 C11 H11 N5
Mr                213.25                     213.25
Dx,g cm-3         1.387                      1.387
Z                 4                          4
Mu (mm-1)         0.729                      0.729
F000              448.0                      448.0
F000'             449.27
h,k,lmax          8,15,15                    8,15,15
Nref              2154                       2111
Tmin,Tmax         0.804,0.896                0.889,1.000
Tmin'             0.804

Correction method= # Reported T Limits: Tmin=0.889 Tmax=1.000
AbsCorr = MULTI-SCAN

Data completeness= 0.980           Theta(max)= 76.628

                                                   wR2(reflections)=
R(reflections)= 0.0340( 1903)                      0.1025( 2111)

S = 1.047                  Npar= 153
```

Alert level C

```
PLAT911_ALERT_3_C Missing FCF Refl Between Thmin & STh/L=    0.600        3 Report
```

Alert level G

```
PLAT012_ALERT_1_G No       _shelx_res_checksum Found in CIF ......     Please Check
PLAT199_ALERT_1_G Reported _cell_measurement_temperature ..... (K)        293 Check
PLAT200_ALERT_1_G Reported   _diffrn_ambient_temperature ..... (K)        293 Check
PLAT912_ALERT_4_G Missing # of FCF Reflections Above STh/L=  0.600         41 Note
PLAT913_ALERT_3_G Missing # of Very Strong Reflections in FCF ....          3 Note
PLAT978_ALERT_2_G Number C-C Bonds with Positive Residual Density.          7 Info
```

0 **ALERT level A** = Most likely a serious problem - resolve or explain
0 **ALERT level B** = A potentially serious problem, consider carefully
1 **ALERT level C** = Check. Ensure it is not caused by an omission or oversight
6 **ALERT level G** = General information/check it is not something unexpected

3 ALERT type 1 CIF construction/syntax error, inconsistent or missing data
1 ALERT type 2 Indicator that the structure model may be wrong or deficient
2 ALERT type 3 Indicator that the structure quality may be low
1 ALERT type 4 Improvement, methodology, query or suggestion
0 ALERT type 5 Informative message, check

Figure S21. CheckCIF/PLATON report for 4-methyl-*N*-(2-(1-tosyl-4,5-dihydro-1*H*-imidazol-2-yl)phthalazin-1(2*H*)-ylidene)benzenesulfonamide (**6c**).

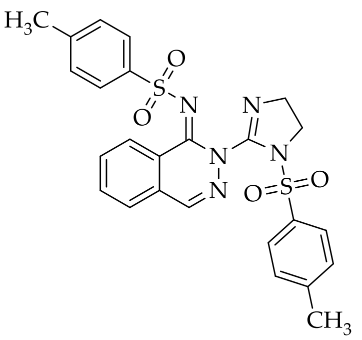

Bond precision: C-C = 0.0037 A Wavelength=0.71073

Cell:	a=9.8874(3)	b=10.3839(3)	c=23.6423(8)
	alpha=90	beta=97.633(3)	gamma=90
Temperature:	293 K		

	Calculated	Reported
Volume	2405.84(13)	2405.83(13)
Space group	P 21/c	P 21/c
Hall group	-P 2ybc	-P 2ybc
Moiety formula	C25 H23 N5 O4 S2	C25 H23 N5 O4 S2
Sum formula	C25 H23 N5 O4 S2	C25 H23 N5 O4 S2
Mr	521.60	521.60
Dx,g cm-3	1.440	1.440
Z	4	4
Mu (mm-1)	0.265	0.265
F000	1088.0	1088.0
F000'	1089.42	
h,k,lmax	12,12,29	12,12,29
Nref	4911	4904
Tmin,Tmax	0.909,0.948	0.991,1.000
Tmin'	0.899	

Correction method= # Reported T Limits: Tmin=0.991 Tmax=1.000
AbsCorr = MULTI-SCAN

Data completeness= 0.999 Theta(max)= 26.372

R(reflections)= 0.0469(3447) wR2(reflections)= 0.1163(4904)

S = 1.021 Npar= 327

Alert level B

PLAT230_ALERT_2_B Hirshfeld Test Diff for S26 --O28 . 7.3 s.u.

Alert level C

PLAT767_ALERT_4_C INS Embedded LIST 6 Instruction Should be LIST 4 Please Check
PLAT906_ALERT_3_C Large K Value in the Analysis of Variance ...... 4.668 Check
PLAT910_ALERT_3_C Missing # of FCF Reflection(s) Below Theta(Min). 6 Note

Alert level G

PLAT199_ALERT_1_G Reported _cell_measurement_temperature ..... (K) 293 Check
PLAT200_ALERT_1_G Reported _diffrn_ambient_temperature ..... (K) 293 Check
PLAT883_ALERT_1_G No Info/Value for _atom_sites_solution_primary . Please Do !
PLAT899_ALERT_4_G SHELXL2018 is Deprecated and Succeeded by SHELXL 2019/3 Note
PLAT978_ALERT_2_G Number C-C Bonds with Positive Residual Density. 1 Info

0 **ALERT level A** = Most likely a serious problem - resolve or explain
1 **ALERT level B** = A potentially serious problem, consider carefully
3 **ALERT level C** = Check. Ensure it is not caused by an omission or oversight
5 **ALERT level G** = General information/check it is not something unexpected

3 ALERT type 1 CIF construction/syntax error, inconsistent or missing data
2 ALERT type 2 Indicator that the structure model may be wrong or deficient
2 ALERT type 3 Indicator that the structure quality may be low
2 ALERT type 4 Improvement, methodology, query or suggestion
0 ALERT type 5 Informative message, check

Figure S22. CheckCIF/PLATON report for 4-methoxy-*N*-(2-(1-((4-methoxyphenyl)sulfonyl)-4,5-dihydro-1*H*-imidazol-2-yl)phthalazin-1(2*H*)-ylidene)benzenesulfonamide (**6e**).

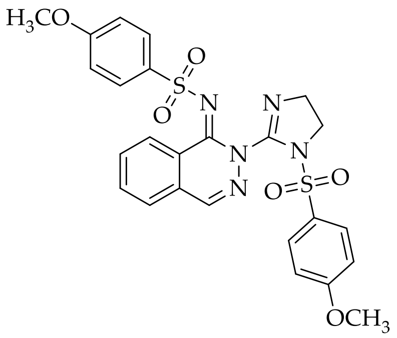


```
Bond precision:   C-C = 0.0034 A          Wavelength=0.71073

Cell:              a=7.5517(2)     b=17.1382(6)       c=19.2240(6)
                   alpha=90        beta=94.443(3)     gamma=90
Temperature:       293 K

                  Calculated                    Reported
Volume            2480.54(13)                   2480.54(13)
Space group       P 21/c                        P 1 21/c 1
Hall group        -P 2ybc                       -P 2ybc
Moiety formula    C25 H23 N5 O6 S2              C25 H23 N5 O6 S2
Sum formula       C25 H23 N5 O6 S2              C25 H23 N5 O6 S2
Mr                553.60                        553.60
Dx,g cm-3         1.482                         1.482
Z                 4                             4
Mu (mm-1)         0.267                         0.267
F000              1152.0                        1152.0
F000'             1153.50
h,k,lmax          9,21,24                       9,21,24
Nref              5080                          5074
Tmin,Tmax         0.899,0.948                   0.987,1.000
Tmin'             0.899

Correction method= # Reported T Limits: Tmin=0.987 Tmax=1.000
AbsCorr = MULTI-SCAN

Data completeness= 0.999            Theta(max)= 26.371

                                                    wR2(reflections)=
R(reflections)= 0.0479( 3545)                       0.1074( 5074)
S = 1.037                  Npar= 345
```

Alert level C

```
PLAT241_ALERT_2_C High   'MainMol' Ueq as Compared to Neighbors of        C13 Check
PLAT411_ALERT_2_C Short Inter H...H Contact  H5       ..H5       .       2.10 Ang.
                                           -x,1-y,1-z  =      3_566 Check
PLAT906_ALERT_3_C Large K Value in the Analysis of Variance ......      5.044 Check
PLAT910_ALERT_3_C Missing # of FCF Reflection(s) Below Theta(Min).          6 Note
```

Alert level G

```
PLAT012_ALERT_1_G No       _shelx_res_checksum Found in CIF ......     Please Check
PLAT199_ALERT_1_G Reported _cell_measurement_temperature ..... (K)        293 Check
PLAT200_ALERT_1_G Reported   _diffrn_ambient_temperature ..... (K)        293 Check
PLAT883_ALERT_1_G No Info/Value for _atom_sites_solution_primary .     Please Do !
PLAT978_ALERT_2_G Number C-C Bonds with Positive Residual Density.          1 Info
PLAT992_ALERT_5_G Repd & Actual _reflns_number_gt Values Differ by          2 Check
```

```
  0 ALERT level A = Most likely a serious problem - resolve or explain
  0 ALERT level B = A potentially serious problem, consider carefully
  4 ALERT level C = Check. Ensure it is not caused by an omission or oversight
  6 ALERT level G = General information/check it is not something unexpected

  4 ALERT type 1 CIF construction/syntax error, inconsistent or missing data
  3 ALERT type 2 Indicator that the structure model may be wrong or deficient
  2 ALERT type 3 Indicator that the structure quality may be low
  0 ALERT type 4 Improvement, methodology, query or suggestion
  1 ALERT type 5 Informative message, check
```

Figure S23. CheckCIF/PLATON report for 2-(((1-((4-chlorophenyl)sulfonyl)imidazolidin-2-ylidene)hydrazono)methyl)benzonitrile (**7g**).

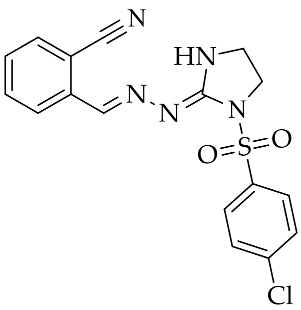

```
Bond precision:   C-C = 0.0081 A          Wavelength=0.71073

Cell:           a=7.6338(8)       b=10.1714(8)      c=11.6609(8)
                alpha=82.556(6)   beta=88.506(7)    gamma=80.477(8)
Temperature:    293 K

                Calculated                  Reported
Volume          885.41(13)                  885.41(13)
Space group     P 1                         P 1
Hall group      P 1                         P 1
Moiety formula  C17 H14 Cl N5 O2 S          C17 H14 Cl N5 O2 S
Sum formula     C17 H14 Cl N5 O2 S          C17 H14 Cl N5 O2 S
Mr              387.84                      387.84
Dx,g cm-3       1.455                       1.455
Z               2                           2
Mu (mm-1)       0.356                       0.356
F000            400.0                       400.0
F000'           400.68
h,k,lmax        9,12,14                     9,12,14
Nref            7272[ 3636]                 7192
Tmin,Tmax       0.918,0.931                 0.979,1.000
Tmin'           0.837

Correction method= # Reported T Limits: Tmin=0.979 Tmax=1.000
AbsCorr = MULTI-SCAN

Data completeness= 1.98/0.99       Theta(max)= 26.372

                                                    wR2(reflections)=
R(reflections)= 0.0486( 5086)                       0.0899( 7192)
S = 0.995                  Npar= 477
```

Alert level C

```
PLAT242_ALERT_2_C Low    'MainMol' Ueq as Compared to Neighbors of      C23A Check
PLAT242_ALERT_2_C Low    'MainMol' Ueq as Compared to Neighbors of      C23B Check
PLAT245_ALERT_2_C U(iso) H16B      Smaller than U(eq) N16B      by     0.012 Ang**2
PLAT334_ALERT_2_C Small <C-C> Benzene Dist.   C20A     -C25A     .      1.37 Ang.
PLAT334_ALERT_2_C Small <C-C> Benzene Dist.   C20B     -C25B     .      1.37 Ang.
PLAT340_ALERT_3_C Low Bond Precision on  C-C Bonds ..............    0.00807 Ang.
PLAT420_ALERT_2_C D-H Bond Without Acceptor  N16A     --H16A     .    Please Check
PLAT911_ALERT_3_C Missing FCF Refl Between Thmin & STh/L=    0.600         2 Report
```

Alert level G

```
PLAT012_ALERT_1_G No       _shelx_res_checksum Found in CIF ......    Please Check
PLAT153_ALERT_1_G The s.u.'s on the Cell Axes   are Equal ..(Note)    0.0008 Ang.
PLAT199_ALERT_1_G Reported _cell_measurement_temperature ..... (K)       293 Check
PLAT200_ALERT_1_G Reported  _diffrn_ambient_temperature ..... (K)       293 Check
PLAT230_ALERT_2_G Hirshfeld Test Diff for    C2A       --C7A     .       5.2 s.u.
PLAT910_ALERT_3_G Missing # of FCF Reflection(s) Below Theta(Min).         4 Note
PLAT978_ALERT_2_G Number C-C Bonds with Positive Residual Density.         0 Info
```

```
0 ALERT level A = Most likely a serious problem - resolve or explain
0 ALERT level B = A potentially serious problem, consider carefully
8 ALERT level C = Check. Ensure it is not caused by an omission or oversight
7 ALERT level G = General information/check it is not something unexpected

4 ALERT type 1 CIF construction/syntax error, inconsistent or missing data
8 ALERT type 2 Indicator that the structure model may be wrong or deficient
3 ALERT type 3 Indicator that the structure quality may be low
0 ALERT type 4 Improvement, methodology, query or suggestion
0 ALERT type 5 Informative message, check
```

Figure S24. CheckCIF/PLATON report for 2-(((1-((4-nitrophenyl)sulfonyl)imidazolidin-2-ylidene)hydrazono)methyl)benzonitrile (**7k**).

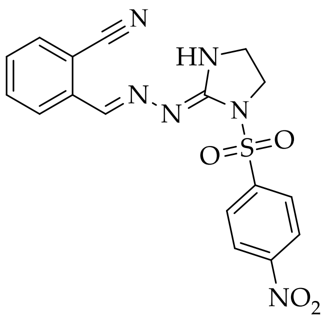

```
Bond precision:   C-C = 0.0052 A          Wavelength=0.71073

Cell:             a=7.2342(11)    b=14.748(3)        c=16.643(2)
                  alpha=90        beta=91.217(14)    gamma=90
Temperature:      293 K

                 Calculated                Reported
Volume           1775.2(5)                 1775.2(5)
Space group      P 21/c                    P 1 21/c 1
Hall group       -P 2ybc                   -P 2ybc
Moiety formula   C17 H14 N6 O4 S           C17 H14 N6 O4 S
Sum formula      C17 H14 N6 O4 S           C17 H14 N6 O4 S
Mr               398.40                    398.40
Dx,g cm-3        1.491                     1.491
Z                4                         4
Mu (mm-1)        0.222                     0.222
F000             824.0                     824.0
F000'            824.84
h,k,lmax         8,17,19                   8,17,19
Nref             3143                      4958
Tmin,Tmax        0.961,0.985               0.979,1.000
Tmin'            0.915

Correction method= # Reported T Limits: Tmin=0.979 Tmax=1.000
AbsCorr = MULTI-SCAN

Data completeness= 1.577          Theta(max)= 25.026

                                                  wR2(reflections)=
R(reflections)= 0.0432( 2608)                     0.0790( 4958)
S = 0.831                   Npar= 258
```

Alert level C

```
PLAT334_ALERT_2_C Small <C-C> Benzene Dist.   C20    -C25    .       1.37 Ang.
PLAT340_ALERT_3_C Low Bond Precision on  C-C Bonds ..............   0.0052 Ang.
PLAT906_ALERT_3_C Large K Value in the Analysis of Variance ......    4.737 Check
```

Alert level G

```
PLAT012_ALERT_1_G No      _shelx_res_checksum Found in CIF ......  Please Check
PLAT199_ALERT_1_G Reported _cell_measurement_temperature ..... (K)     293 Check
PLAT200_ALERT_1_G Reported   _diffrn_ambient_temperature ..... (K)     293 Check
PLAT870_ALERT_4_G ALERTS Related to Twinning Effects Suppressed ..       ! Info
PLAT910_ALERT_3_G Missing # of FCF Reflection(s) Below Theta(Min).       2 Note
PLAT931_ALERT_5_G CIFcalcFCF  Twin Law ( 0 0 1)        Est.d BASF     0.38 Check
PLAT931_ALERT_5_G CIFcalcFCF  Twin Law ( 1 0 0)        Est.d BASF     0.31 Check
```

```
0 ALERT level A = Most likely a serious problem - resolve or explain
0 ALERT level B = A potentially serious problem, consider carefully
3 ALERT level C = Check. Ensure it is not caused by an omission or oversight
7 ALERT level G = General information/check it is not something unexpected

3 ALERT type 1 CIF construction/syntax error, inconsistent or missing data
1 ALERT type 2 Indicator that the structure model may be wrong or deficient
3 ALERT type 3 Indicator that the structure quality may be low
1 ALERT type 4 Improvement, methodology, query or suggestion
2 ALERT type 5 Informative message, check
```

The crystal packing of molecules in **6e** and **6c** is shown in **Figure S25**. In **6e** the molecules are arranged into centrosymmetric dimers via π-π stacking interactions between phthalazinimine fragments. Those dimers are further assembled into 1D chains along [010] via stacking interactions between 4-methoxyphenyl groups of the sulfonyl substituent at the imidazolidine ring. Stacking interactions between 1-phthalazinimine fragments are absent in **6e** where 4-methyl groups are already involved in π-π intramolecular stacking. Here intermolecular stacking interactions between one of the 4-methylphenyl groups and 1-phthalazinimine fragment of inversion center related molecule result in compact dimers shown in **Figure S26**.

Figure S25. Crystal packing in **6e** (left) and **6c** (right).

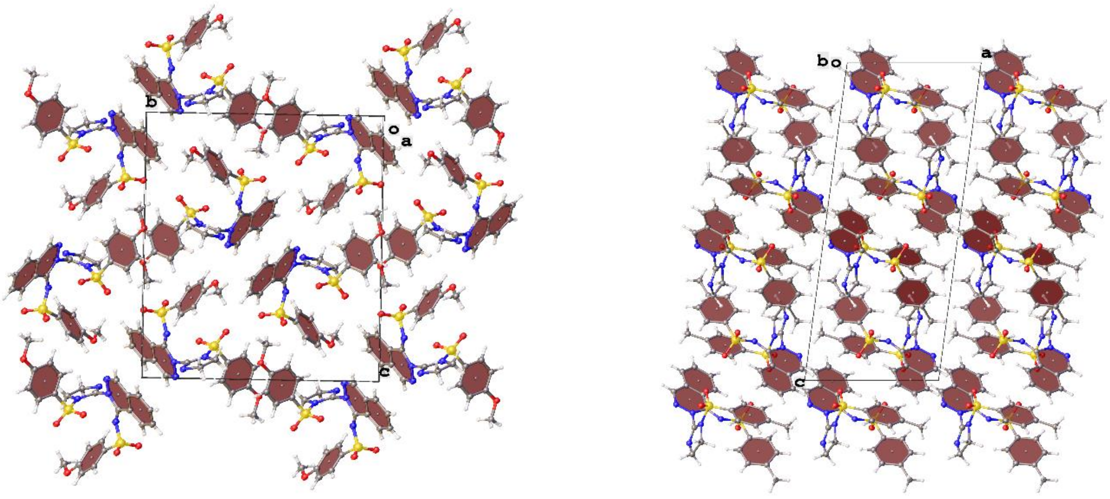

Figure S26. Centrosymmetric dimer via π-π stacking interactions in **6c**.

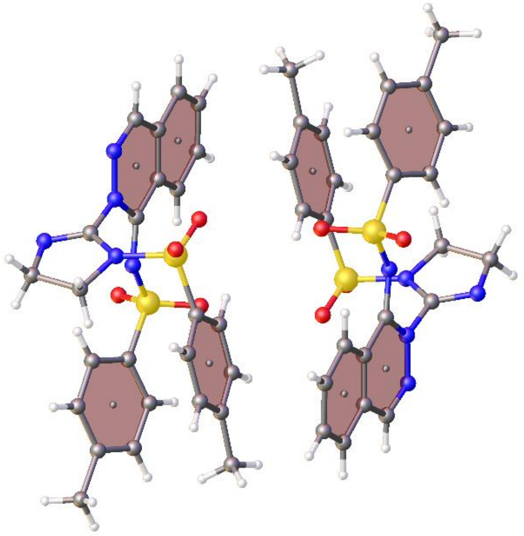

The crystal packing of molecules in **7g** and **7k** as shown in **Figure S27** is mostly driven by weak intermolecular forces. Among specific interactions the main role is played by stacking

interactions between aromatic rings, or aromatic ring and extended π-systems, as well as weak N-H···N (**7g**) or N-H···O (**7k**) hydrogen bonds.

Figure S27. Crystal packing in **7g** and **7k**. Hydrogen bonds are shown with dashed lines.

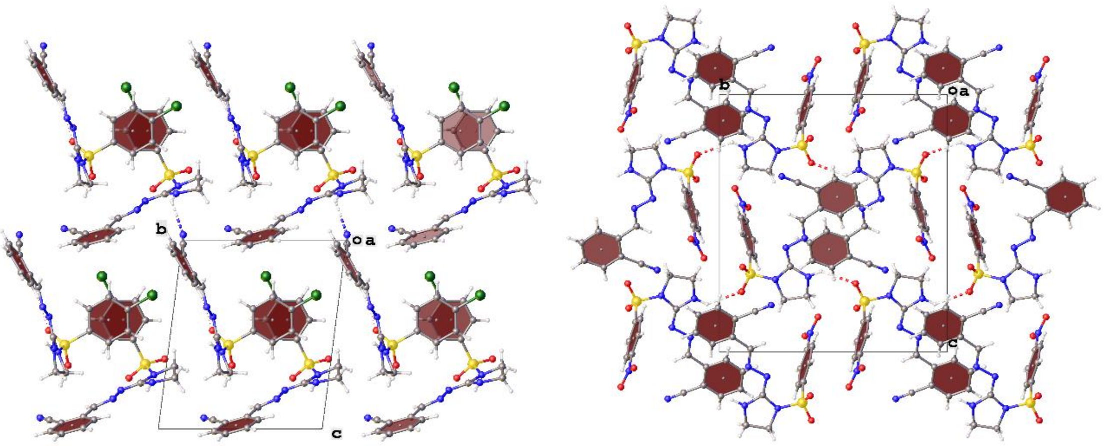

Supplement: Supplementary file 1 [file ijms-25-11495-s001.zip › ijms-3256894-supplementary.pdf]
